# Radiosynthesis and Preclinical Evaluation of *m*-[^18^F]FET and [^18^F]FET-OMe as Novel [^18^F]FET Analogs for Brain Tumor Imaging

**DOI:** 10.1021/acs.molpharmaceut.3c01215

**Published:** 2024-05-15

**Authors:** Benedikt Gröner, Chris Hoffmann, Heike Endepols, Elizaveta A. Urusova, Melanie Brugger, Felix Neumaier, Marco Timmer, Bernd Neumaier, Boris D. Zlatopolskiy

**Affiliations:** † Forschungszentrum Jülich GmbH, Nuclear Chemistry (INM-5), Institute of Neuroscience and Medicine, Wilhelm-Johnen-Straße, Jülich 52428, Germany; ‡ Faculty of Medicine and University Hospital Cologne, Institute of Radiochemistry and Experimental Molecular Imaging, University of Cologne, Kerpener Straße 62, Cologne 50937, Germany; § Faculty of Medicine and University Hospital Cologne, Department of Nuclear Medicine, University of Cologne, Kerpener Straße 62, Cologne 50937, Germany; ∥ Faculty of Medicine and University Hospital Cologne, Center for Neurosurgery, Department of General Neurosurgery, University of Cologne, Kerpener Straße 62, Cologne 50937, Germany

**Keywords:** fluorine-18, positron emission tomography, radiopharmaceuticals, [^18^F]FET, brain tumor imaging

## Abstract

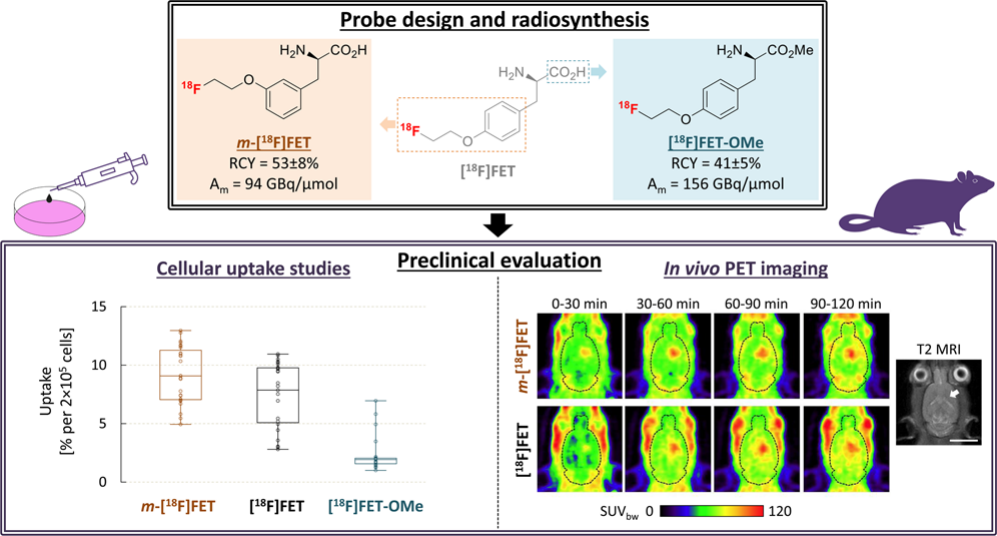

*O*-([^18^F]Fluoroethyl)-l-tyrosine ([^18^F]FET) is actively transported into the brain and cancer cells by LAT1 and possibly other amino acid transporters, which enables brain tumor imaging by positron emission tomography (PET). However, tumor delivery of this probe in the presence of competing amino acids may be limited by a relatively low affinity for LAT1. The aim of the present work was to evaluate the *meta*-substituted [^18^F]FET analog *m*-[^18^F]FET and the methyl ester [^18^F]FET-OMe, which were designed to improve tumor delivery by altering the physicochemical, pharmacokinetic, and/or transport properties. Both tracers could be prepared with good radiochemical yields of 41–56% within 66–90 min. Preclinical evaluation with [^18^F]FET as a reference tracer demonstrated reduced *in vitro* uptake of [^18^F]FET-OMe by U87 glioblastoma cells and no advantage for *in vivo* tumor imaging. In contrast, *m*-[^18^F]FET showed significantly improved *in vitro* uptake and accelerated *in vivo* tumor accumulation in an orthotopic glioblastoma model. As such, our work identifies *m*-[^18^F]FET as a promising alternative to [^18^F]FET for brain tumor imaging that deserves further evaluation with regard to its transport properties and *in vivo* biodistribution.

## 1 Introduction

**Figure 1:**
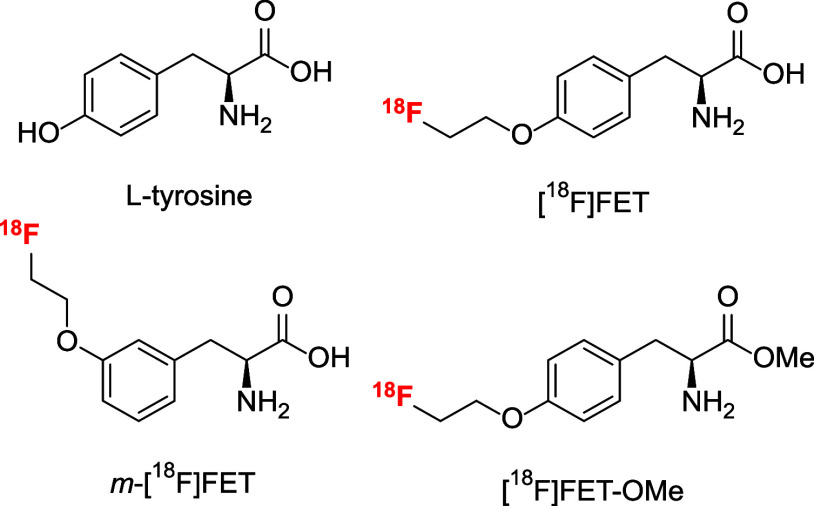
Structure of l-tyrosine and the established PET-tracer *O*-([^18^F]fluoroethyl)-l-tyrosine ([^18^F]FET) as well as of the two novel [^18^F]FET analogs, *O*-([^18^F]fluoroethyl)-l-*meta*-tyrosine (*m*-[^18^F]FET) and *O*-([^18^F]fluoroethyl)-l-tyrosine methyl ester ([^18^F]FET-OMe), described in the present study.

Positron emission tomography (PET) imaging with probes capable to visualize tumor-associated proliferation markers is widely used in the diagnosis and treatment of neoplastic diseases. While detection of increased tumoral glucose metabolism with 2-[^18^F]fluoro-2-deoxy-d-glucose ([^18^F]FDG) has become the gold standard for PET imaging of peripheral tumors, the high physiological uptake of [^18^F]FDG by healthy brain tissue hampers the use of this tracer for visualizing brain tumors like gliomas.^1,2^ In contrast, tracers derived from amino acids like *O*-([^18^F]fluoroethyl)-l-tyrosine ([^18^F]FET, Figure 1) often exhibit high tumor accumulation but low uptake by normal brain tissue, enabling the detection and characterization of cerebral tumors with greater sensitivity than [^18^F]FDG.^1,2^ As such, PET imaging with [^18^F]FET has proven to be a versatile tool for the management of brain tumor patients that facilitates the delineation, differential diagnosis, grading, and treatment of gliomas or other intracranial tumors.^3−5^ Although [^18^F]FET does not significantly participate in specific metabolic pathways, it is actively transported across the blood-brain-barrier (BBB) and into cells by dedicated amino acid transport systems,^6−9^ which are upregulated in most cancer cells.^10−12^ The majority of [^18^F]FET transport is generally thought to be mediated by LAT1 and possibly other members of system L,^6−9,13,14^ which possess a large maximal transport capacity (i.e., high *V*_max_) and a relatively high affinity (i.e., low *K*_*m*_) for large neutral amino acids like l-tyrosine and l-phenylalanine.^15^ In addition to endogenous substrates, LAT1 has been demonstrated to transport a broad range of amino acid derivatives and prodrugs with an amino acid promoiety.^16−18^ Although substitution of the aromatic ring in tyrosine and phenylalanine derivatives with small, moderately lipophilic groups is typically well tolerated by LAT1,^19−22^ [^18^F]FET has been reported to be a substrate with relatively low affinity.^13,23^ This could limit *in vivo* transport of the probe in the presence of competing neutral amino acids. Interestingly, *meta*-substituted amino acids have consistently been found to exhibit enhanced transport capacity and/or binding affinity relative to the corresponding *para*- or *ortho*-substituted derivatives,^19−22,24^ suggesting that [^18^F]FET analogs containing the [^18^F]fluoroethoxy group in the *meta* instead of *para* position could display improved transport properties. Other aspects of the structure–activity relationship (SAR) for substrates of LAT1, such as the role of amino and carboxylic acid functions, remain controversial. Thus, while most previous studies concluded that the presence of both free amino and carboxyl groups is essential for substrate recognition,^16,18,25,26^ some recent findings indicate that carboxylic esters and hydroxamic acid derivatives of amino acids may also be transported by LAT1.^24,27,28^ Accordingly, esterification or replacement of the carboxylic acid function in [^18^F]FET could potentially be used to modify the physicochemical and pharmacokinetic profile of the probe while retaining tumor uptake via LAT1. Alternatively, or in addition, [^18^F]FET esters could serve as prodrugs that improve brain tumor delivery by altering factors not related to active transport across the BBB (e.g., passive transfer across diffusion barriers like surrounding normal brain tissue) and are cleaved into unmodified [^18^F]FET by brain esterases.

In the present work, we report the synthesis of *O*-([^18^F]fluoroethyl)-l-*meta*-tyrosine (*m*-[^18^F]FET) and *O*-([^18^F]fluoroethyl)-l-tyrosine methyl ester ([^18^F]FET-OMe) (Figure 1), two novel analogs of [^18^F]FET designed based on the SAR considerations summarized above. In addition, we describe the results of a preclinical evaluation of both probes *in vitro* and *in vivo*, using [^18^F]FET as a reference tracer.

## 2 Materials and Methods

### 2.1 Organic Chemistry

#### 2.1.1 General

Unless noted otherwise, all reagents and solvents were purchased from Sigma-Aldrich (Steinheim, Germany), Acros (Fisher Scientific GmbH, Nidderrau, Germany), Alfa Aesar [Thermo Fisher (Kandel) GmbH, Kandel, Germany], BLDPharm (Kaiserslautern, Germany), or Key Organics (Camelford, UK) and used without further purification. Unless otherwise stated, all reactions were carried out with magnetic stirring and, if air- or moisture-sensitive substrates and/or reagents were used, in flame-dried glassware under argon. Organic extracts were dried over anhydrous Na_2_SO_4_ or MgSO_4_. Solutions were concentrated under reduced pressure (1–900 mbar) at 40–50 °C using a rotary evaporator. **1** was prepared according to the literature.^29^ Proton, carbon, and fluorine nuclear magnetic resonance (^1^H-, ^13^C-, and ^19^F-NMR) spectra were recorded at ambient temperature in deuterium oxide (D_2_O), deuterochloroform (CDCl_3_), or dimethyl sulfoxide-d_6_ [(CD_3_)_2_SO] (as indicated) using a Bruker Avance Neo (400 MHz) spectrometer. The measured chemical shifts are reported in parts per million (ppm) relative to residual peaks of deuterated solvents. The observed signal multiplicities are characterized as follows: s = singlet, d = doublet, *t* = triplet, m = multiplet, br = broad, br d = broad doublet, dd = doublet of doublets, ddd = doublet of doublets of doublets, dt = doublet of triplets, td = triplet of doublets, and qd = quartet of doublets. Coupling constants (*J*) are reported in hertz (Hz). The *ortho*- and *meta*-carbons of the phenyl substituent are not equivalent owing to hindered rotation. Low-resolution mass spectrometry (LRMS) was performed with an MSQ PlusTM mass spectrometer (Thermo Electron Corporation, San Jose, USA). High-resolution mass spectrometry (HRMS) was performed with an LTQ Orbitrap XL spectrometer (Thermo Fischer Scintific Inc., Bremen, Germany). The enantiomeric excess (*ee*) of the nonradioactive reference compounds HCl·(*RS*)-*m*-FET, *m*-FET, (*RS*)-FET, and FET was determined by chiral HPLC [column: Astec Chirobiotic T, 5 μm, 250 × 4.6 mm; eluent: 55% MeOH (0.02% HCO_2_H); flow rate: 1.0 mL/min].

#### 2.1.2 Preparation of Ni-Complex **2**

A solution of (*S*)-*N*-(2-benzoyl-4-chlorophenyl)-1-(3,4-dichlorobenzyl)pyrrolidine-2-carboxamide (**1**)^30^ (8.1 g, 16.6 mmol), (*RS*)-*m*-Tyr (5.89 g, 32.5 mmol), Ni(OAc)_2_·4 H_2_O (8.09 g, 32.5 mmol), and K_2_CO_3_ (20.4 g, 147.75 mmol) in anhydrous MeOH (400 mL) was stirred at 60 °C for 24 h and at ambient temperature for 72 h. The reaction mixture was poured into an ice-cold solution of AcOH (50 mL) in H_2_O (3 L), and the resulting suspension was allowed to stand at ambient temperature for 24 h, after which a fine red precipitate had formed. The precipitate was collected by filtration, washed with H_2_O (3 × 100 mL), air-dried, and dissolved in EtOAc (200 mL). The resulting solution was washed with H_2_O (3 × 50 mL) and brine (2 × 50 mL), dried, and concentrated under reduced pressure. The residue was triturated with Et_2_O to give a red precipitate, which was recrystallized from EtOAc/hexane to afford a first crop of the title compound. The combined mother liquors (from trituration with Et_2_O and recrystallization) were concentrated under reduced pressure, and the residue was purified by column chromatography (CHCl_3_:acetone = 5:1) followed by recrystallization from EtOAc/hexane to afford a second crop of the title compound (total yield: 10.35 g, 88%) as a red solid.^31^*R*_*f*_ = 0.25 (broad spot; CHCl_3_:acetone = 5:1). ^1^H NMR (400 MHz, CDCl_3_) δ 8.90 (s, 1H), 8.30 (br, 1H), 8.09 (d, *J* = 8.9 Hz, 1H), 7.81–7.41 (m, 4H), 7.40–7.20 (m, 3H), 7.10 (d, *J* = 8.5 Hz, 1H), 6.91 (dd, *J* = 22.3, 6.9 Hz, 2H), 6.80–6.70 (m, 1H), 6.69–6.53 (m, 2H), 4.29 (s, 1H), 4.13 (d, *J* = 12.4 Hz, 1H), 3.33–3.16 (m, 1H), 3.09 (d, *J* = 11.6 Hz, 2H), 2.96 (d, *J* = 12.4 Hz, 1H), 2.67 (d, *J* = 8.8 Hz, 1H), 2.45–2.19 (m, 3H), 2.17–1.83 (m, 2H). ^13^C NMR (101 MHz, CDCl**_3_**) δ 180.18, 179.11, 171.01, 157.69, 140.92, 136.76, 135.03, 133.81, 133.44, 133.36, 133.30, 132.59, 132.51, 131.06, 130.47, 130.17, 129.91, 129.61, 129.37, 127.82, 127.44, 127.23, 125.95, 123.95, 122.23, 117.65, 115.27, 71.66, 71.59, 63.46, 58.77, 39.17, 30.93, 23.12. MS (ESI) *m*/*z*: [M + H]^+^ calculated for C_34_H_29_Cl_3_N_3_NiO_4_^+^: 708.06; found: 707.98. MS (ESI) *m*/*z*: [M–H]^−^ calculated for C_34_H_27_Cl_3_N_3_NiO_4_^–^: 706.04; found: 705.93. Correct isotopic pattern.

#### 2.1.3 Preparation of Tosylate **3**

Cs_2_CO_3_ (0.46 g, 1.41 mmol) was added to a solution of **2** (1 g, 1.41 mmol) in anhydrous MeCN (30 mL), and the reaction mixture was stirred at 70 °C for 1 h. After cooling to ambient temperature, (CH_2_)_2_(OTs)_2_ (0.7 g, 1.89 mmol) was added, and the mixture was stirred at 50 °C for another 48 h. The resulting suspension was allowed to cool to ambient temperature, the precipitate was removed by filtration, and the filtrate was concentrated under reduced pressure. The residue was purified by column chromatography (CHCl_3_:acetone = 5:1) to afford the title compound (0.6 g, 47%) as a red foam. ^1^H NMR (400 MHz, CDCl_3_) δ 8.91 (s, 1H), 8.13 (d, *J* = 9.3 Hz, 1H), 7.79 (d, *J* = 7.9 Hz, 2H), 7.63 (d, *J* = 7.6 Hz, 1H), 7.60–7.51 (m, 2H), 7.45–7.40 (m, 1H), 7.34 (d, *J* = 7.9 Hz, 2H), 7.32–7.28 (m, 2H), 7.26 (d, *J* = 4.2 Hz, 1H), 7.08 (d, *J* = 7.3 Hz, 1H), 6.86 (d, *J* = 7.3 Hz, 1H), 6.72 (t, *J* = 7.3 Hz, 2H), 6.62–6.54 (m, 2H), 5.29 (s, 1H), 4.35–4.20 (m, 3H), 4.16 (d, *J* = 12.4 Hz, 1H), 4.06–3.90 (m, 2H), 3.29–3.13 (m, 2H), 3.09 (d, *J* = 12.8 Hz, 2H), 2.78 (dd, *J* = 13.6, 5.1 Hz, 1H), 2.44 (s, 3H), 2.30–2.21 (m, 1H), 2.02–1.88 (m, 1H), 1.87–1.64 (m, 2H). ^13^C NMR (101 MHz, CDCl_3_) δ 179.94, 178.27, 170.85, 158.70, 145.10, 141.07, 137.20, 135.07, 133.81, 133.44, 133.37, 133.26, 132.92, 132.65, 132.52, 131.07, 130.40, 130.03 (×2), 129.84, 129.54, 129.30, 128.14, 127.80, 127.33, 127.16, 125.79, 123.87, 123.66, 115.64, 114.82, 71.71, 71.55, 68.30, 65.54, 63.42, 58.69, 40.01, 30.95, 23.19, 21.78. MS (ESI) *m*/*z*: [M + H]^+^ calculated for C_43_H_39_C_l3_N_3_NiO_7_S^+^: 906.09; found: 906.44. Correct isotopic pattern.

#### 2.1.4 Preparation of Ni-Complex **4**

**4** (1.0 g, 94%, red solid) was prepared from **2** (1 g, 1.41 mmol), 1-bromo-2-fluoroethane (0.32 mL, 0.55 g, 4.30 mmol), and Cs_2_CO_3_ (0.92 g, 2.82 mmol) in anhydrous MeCN (30 mL) using the same procedure as described for preparation of **3**, except that the reaction time at 50 °C after addition of the alkylating agent was reduced to 5 h. The crude product was purified by trituration with Et_2_O. ^1^H NMR (400 MHz, CDCl**_3_**) δ 8.91 (d, *J* = 2.0 Hz, 1H), 8.15 (d, *J* = 9.3 Hz, 1H), 7.64 (dd, *J* = 8.2, 2.1 Hz, 1H), 7.60–7.50 (m, 2H), 7.45–7.39 (m, 1H), 7.34–7.27 (m, 3H), 7.10 (dd, *J* = 9.3, 2.6 Hz, 1H), 7.02–6.95 (m, 1H), 6.77–6.73 (m, 1H), 6.72–6.70 (m, 1H), 6.70–6.67 (m, 1H), 6.59 (d, *J* = 2.6 Hz, 1H), 4.80–4.53 (m, 2H), 4.26 (t, *J* = 5.1 Hz, 1H), 4.17 (d, *J* = 12.5 Hz, 1H), 4.13–3.94 (m, 2H), 3.25–3.08 (m, 4H), 2.80 (dd, *J* = 13.8, 5.4 Hz, 1H), 2.56–2.26 (m, 3H), 1.98–1.89 (m, 1H), 1.86–1.76 (m, 1H). ^13^C NMR (101 MHz, CDCl**_3_**) δ 179.90, 178.30, 170.84, 159.03, 141.10, 137.18, 135.04, 133.81, 133.45, 133.39, 133.29, 132.65, 132.52, 131.07, 130.36, 130.10, 129.83, 129.52, 129.28, 127.81, 127.34, 127.17, 125.78, 123.87, 123.53, 115.60, 115.03, 81.97 (d, *J* = 170.8 Hz), 71.75, 71.57, 67.12 (d, *J* = 20.2 Hz), 63.41, 58.66, 40.07, 30.95, 23.17. ^19^F-NMR (376 MHz, CDCl**_3_**) δ −223.51. MS (ESI) *m*/*z*: [M + H]^+^ calculated for C_36_H_32_Cl_3_FN_3_NiO_4_^+^: 754.08; found: 754.40. Correct isotopic pattern.

#### 2.1.5 Preparation of (*S*)-2-Amino-3-[3-(2-fluoroethoxy)phenyl]propanoic Acid (*m*-FET) from **4**

6 n HCl (10 mL) was added dropwise at 65 °C to a stirred solution of **4** (0.5 g, 0.66 mmol) in MeOH (30 mL), and the reaction mixture was stirred at 65 °C until the color had changed from red to light green (approximately 40 min). The mixture was allowed to cool to ambient temperature and concentrated under reduced pressure. The residue was taken up into H_2_O (30 mL), and the pH value was adjusted by the dropwise addition of 3% NH_3_ to approximately 7.5. The resulting suspension was washed with CH_2_Cl_2_ (3 × 20 mL), which was dried and concentrated under reduced pressure to recycle ligand **1**. The remaining aqueous fraction was concentrated under reduced pressure, and the residue was triturated with H_2_O. The resulting precipitate was collected by filtration, washed with acetone and Et_2_O, and dried to afford the title compound (0.14 g, 93%) as a colorless solid. ^1^H NMR [400 MHz, 10% TFA in (CD_3_)_2_SO] δ 8.30 (d, *J* = 16.9 Hz, 1H), 7.19 (t, *J* = 6.8 Hz, 1H), 6.87–6.80 (m, *J* = 10.8 Hz, 2H), 4.68 (d, *J* = 47.7 Hz, 2H), 4.20 (s, 1H), 4.15 (d, *J* = 15.2 Hz, 2H), 3.12–2.93 (m, 2H). ^13^C NMR [101 MHz, 10% TFA in (CD_3_)_2_SO] δ 170.54, 158.54, 136.54, 129.98, 122.32, 116.02, 113.57, 82.31 (d, *J* = 166.8 Hz), 67.14 (d, *J* = 19.0 Hz), 53.19, 35.92. ^19^F-NMR [376 MHz, 10% TFA in (CD_3_)_2_SO] δ −222.51. MS (ESI) *m*/*z*: [M + H]^+^ calculated for C_11_H_15_FNO_3_^+^: 228.11; found: 228.24. HR-MS (ESI) *m*/*z*: [M + H]^+^ calculated for C_11_H_15_FNO_3_^+^: 228.10305; found: 228.10328.

#### 2.1.6 Preparation of (*RS*)-2-Amino-3-[3-(2-fluoroethoxy)phenyl]propanoate Hydrochloride [HCl·(*RS*)-*m*-FET]

##### 2.1.6.1 Boc-(*RS*)-*m*-Tyr-OMe^32^

SOCl_2_ (2 mL, 3.28 g, 27.57 mmol) was added dropwise to a vigorously stirred, ice-cold suspension of (*RS*)-*m*-Tyr (1 g, 5.52 mmol) in anhydrous MeOH (30 mL). After stirring for 30 min, the cooling bath was removed and the reaction mixture was stirred for another 16 h. That followed, all volatiles were removed under reduced pressure, and the residue was dried at 2 mbar and 50 °C for 1 h. The intermediate HCl·(*RS*)-*m*-Tyr-OMe thus obtained was dissolved in MeOH (40 mL) and used for the next step without any further purification and characterization. An aqueous solution of NaHCO_3_ (1.4 g in 25 mL H_2_O), followed by Boc_2_O (2.4 g), and, if required to obtain a homogeneous solution, additional H_2_O and/or MeOH were then added, and the resulting mixture was stirred for 16 h. The MeOH was removed under reduced pressure, and the remaining emulsion was extracted with EtOAc (2 × 50 mL). The organic fraction was washed with H_2_O (3 × 30 mL), 1 n NaHSO_4_ (3 × 30 mL), 10% NaHCO_3_ (3 × 30 mL), and brine (2 × 30 mL), dried, and concentrated under reduced pressure. The residue was triturated with *n*-hexane to afford the title compound (1.06 g, 65% over two steps) as a beige solid. The spectral data of the substance were in accordance with the literature.^33^

##### 2.1.6.2 Boc-(*RS*)-*m*-FET-OMe

The title compound (0.9 g, 78%; colorless solid) was prepared from Boc-(*RS*)-Tyr-OMe (1 g, 3.40 mmol), 1-fluoro-2-iodoethane (1 mL, 2.16 g, 12.42 mmol), and Cs_2_CO_3_ (1.87 g, 5.74 mmol) in anhydrous MeCN (20 mL) using the same procedure as described for the preparation of **3**, except that the reaction time at 50 °C after addition of the alkylating agent amounted to 16 h. The crude product was purified by recrystallization from EtOAc/pentane. ^1^H NMR (400 MHz, CDCl_3_) δ 7.21 (t, *J* = 7.8 Hz, 1H), 6.81 (dd, *J* = 8.2, 2.1 Hz, 1H), 6.74 (d, *J* = 7.8 Hz, 1H), 6.70 (s, 1H), 4.98 (d, *J* = 7.8 Hz, 1H), 4.80 (dd, *J* = 4.8, 3.6 Hz, 1H), 4.72–4.65 (m, 1H), 4.22 (dd, *J* = 4.8, 3.6 Hz, 1H), 4.19–4.14 (m, 1H), 3.72 (s, 3H), 3.05 (qd, *J* = 13.8, 6.0 Hz, 2H), 1.42 (s, 9H). ^13^C NMR (101 MHz, CDCl_3_) δ 172.42, 158.66, 155.21, 137.83, 129.76, 122.37, 115.88, 113.27, 82.02 (d, *J* = 170.7 Hz), 80.10, 67.17 (d, *J* = 20.6 Hz), 54.44, 52.38, 38.45, 28.42. ^19^F-NMR (376 MHz, CDCl_3_) δ −223.87. MS (ESI) *m*/*z*: [M + Na]^+^ calculated for C_17_H_24_FNO_5_Na^+^: 364.15; found: 364.10; [M + H]^+^ calculated for C_17_H_25_FNO_5_^+^: 342.17; found: 342.16.

##### 2.1.6.3 Boc-(*RS*)-*m*-FET-OH

1 n NaOH (7.1 mL) was added to a solution of Boc-(*RS*)-*m*-FET-OMe (0.81 g, 2.37 mmol) in THF (50 mL), and the reaction mixture was stirred at ambient temperature for 1 h and at 50 °C for 20 min, after which TLC analysis indicated complete hydrolysis of the starting material. THF was removed under reduced pressure and the residue was partitioned between EtOAc and 1 n NaHSO_4_ (80 mL of each). The organic fraction was separated, washed with 1 n NaHSO_4_ (3 × 30 mL), H_2_O (3 × 30 mL), and brine (2 × 30 mL), dried, and concentrated under reduced pressure. The residue was purified by trituration with Et_2_O to afford the title compound (0.6 g, 77%) as a colorless solid. The NMR spectra showed the presence of two rotamers. Only data for the major rotamer are reported. ^1^H NMR [400 MHz, CD_3_OD] δ 7.20 (dd, *J* = 9.9, 6.1 Hz, 1H), 6.91–6.75 (m, 3H), 4.80–4.71 (m, 1H), 4.68–4.59 (m, 1H), 4.40–4.29 (m, 1H), 4.26–4.21 (m, 1H), 4.15 (ddd, *J* = 12.8, 6.5, 5.1 Hz, 1H), 3.14 (dd, *J* = 13.8, 5.0 Hz, 1H), 2.86 (dt, *J* = 22.8, 11.4 Hz, 1H), 1.37 (s, 9H). ^13^C NMR [101 MHz, CD_3_OD] δ 160.04, 157.78, 140.25, 130.44, 123.12, 116.63, 113.96, 83.15 (d, *J* = 168.8 Hz), 80.53, 68.48 (d, *J* = 19.9 Hz), 56.18, 38.72, 28.66. ^19^F-NMR [376 MHz, CD_3_OD] δ −225.38. MS (ESI) *m*/*z*: [M + Na]^+^ calculated for C_16_H_22_FNO_5_Na^+^: 350.14; found: 350.10; [M–H]^−^ calculated for C_16_H_21_FNO_5_^–^: 326.14; found: 326.11.

##### 2.1.6.4 HCl·(*RS*)-*m*-FET

AcCl (6.5 mL, 7.15 g, 91.09 mmol) was added dropwise to an ice-cold solution of anhydrous MeOH (3.9 mL, 3.09 g, 96.40 mmol) in EtOAc (25 mL), and the resulting solution was stirred for 15 min and added to a solution of Boc-(*RS*)-*m*-FET-OH (0.5 g, 1.53 mmol) in EtOAc (3 mL). After incubation for 1 h at ambient temperature, the resulting suspension was concentrated under reduced pressure, and the residue was purified by trituration with Et_2_O to give the title compound (0.26 g, 65%; total yield of 25% over five steps) as a colorless solid. ^1^H NMR (400 MHz, D_2_O) δ 7.29 (t, *J* = 7.9 Hz, 1H), 6.95–6.83 (m, 3H), 4.78 (dd, *J* = 4.6, 3.2 Hz, 1H), 4.66 (dd, *J* = 4.7, 3.2 Hz, 1H), 4.26 (dd, *J* = 4.7, 3.2 Hz, 1H), 4.22 (dd, *J* = 7.6, 5.6 Hz, 1H), 4.19 (dd, *J* = 4.7, 3.2 Hz, 1H), 3.24 (dd, *J* = 14.5, 5.6 Hz, 1H), 3.10 (dd, *J* = 14.6, 7.7 Hz, 1H). ^13^C NMR (101 MHz, D_2_O) δ 171.57, 158.20, 135.90, 130.56, 122.66, 115.80, 114.22, 82.74 (d, *J* = 164.1 Hz), 67.57 (d, *J* = 18.4 Hz), 54.16, 35.58. ^19^F-NMR (376 MHz, D_2_O) δ −223.26. MS (ESI) *m*/*z*: [M + H]^+^ calculated for C_11_H_15_FNO_3_^+^: 228.10; found: 228.10; [M+Cl]^−^ calculated for C_11_H_14_FNO_3_Cl^–^: 262.06; found: 262.14.

#### 2.1.7 Preparation of (*S*)-2-Amino-3-[3-(2-fluoroethoxy)phenyl]propanoic Acid (*m*-FET) from HCl·(*RS*)-*m*-FET

##### 2.1.7.1 Ni-Complex **5**

HCl·(*RS*)-*m*-FET (0.237 g, 0.9 mmol) was added to a vigorously stirred suspension of Ni(OAc)_2_·4 H_2_O (0.224 g, 0.9 mmol) in a solution of (*S*)-2-[*N*-(*N*′-benzylprolyl)amino]-benzophenone [(*S*)-BPB]^34^ (0.314 g, 0.82 mmol) and K_2_CO_3_ (0.831 g, 6.01 mmol) in MeOH (14 mL). The mixture was stirred at 50 °C until TLC indicated virtually complete consumption of the (*S*)-BPB (approximately 5 h) and then poured into aqueous AcOH (0.35 mL, 0.36 g, 6.38 mmol in 100 mL H_2_O). The resulting red precipitate was collected by filtration, washed with H_2_O (60 mL), air dried, and washed with pentane (60 mL). The crude product was recrystallized from CH_2_Cl_2_/Et_2_O to afford **5** (0.486 g, 91%) as a red solid. ^1^H NMR (400 MHz, CDCl_3_) δ 8.23 (d, *J* = 8.6 Hz, 1H), 8.00 (d, *J* = 7.1 Hz, 2H), 7.51 (qd, *J* = 7.4, 3.8 Hz, 2H), 7.40 (td, *J* = 7.5, 1.3 Hz, 1H), 7.33–7.27 (m, 4H), 7.19–7.09 (m, 2H), 6.97 (dd, *J* = 8.0, 2.1 Hz, 1H), 6.78 (d, *J* = 7.5 Hz, 1H), 6.77–6.72 (m, 1H), 6.70 (d, *J* = 9.9 Hz, 1H), 6.69–6.59 (m, 2H), 4.77–4.64 (m, 1H), 4.65–4.51 (m, 1H), 4.26 (dd, *J* = 13.3, 8.8 Hz, 2H), 4.13–4.04 (m, 1H), 4.04–3.95 (m, 1H), 3.52–3.43 (m, 1H), 3.31 (dd, *J* = 9.7, 7.4 Hz, 1H), 3.14 (ddd, *J* = 14.1, 7.3, 4.2 Hz, 2H), 2.86 (dd, *J* = 13.7, 5.5 Hz, 1H), 2.59–2.43 (m, 1H), 2.42–2.26 (m, 2H), 1.97 (td, *J* = 10.4, 6.5 Hz, 1H), 1.82–1.72 (m, 1H). ^13^C NMR (101 MHz, CDCl_3_) δ 180.38, 178.67, 171.24, 158.96, 142.96, 137.45, 134.22, 133.69, 133.37, 132.53, 131.62, 130.01, 129.88, 129.16, 128.98, 128.91 (×2), 128.07, 127.30, 126.18, 123.54, 123.44, 120.70, 115.57, 114.98, 82.01 (d, *J* = 170.7 Hz), 71.56, 70.48, 67.12 (d, *J* = 20.2 Hz), 63.46, 57.35, 40.21, 30.79, 23.34. ^19^F-NMR (376 MHz, CDCl_3_) δ −223.59. MS (ESI) *m*/*z*: [M + Na]^+^ calculated for C_36_H_34_FN_3_NiO_4_Na^+^: 672.18; found: 672.15; [M + H]^+^ calculated for C_36_H_35_FN_3_NiO_4_^+^: 650.20; found: 650.17. Correct isotopic pattern.

##### 2.1.7.2 (*S*)-2-Amino-3-[3-(2-fluoroethoxy)phenyl]propanoic Acid (*m*-FET)

6 n HCl (3 mL) was added dropwise at 65 °C to a stirred suspension of **5** (0.466 g, 7.07 mmol) in MeOH (10 mL), and the reaction mixture was stirred at 65 °C until the color had changed from red to light green (approximately 15 min). The mixture was allowed to cool to ambient temperature, concentrated under reduced pressure, and dried at 50 °C and 2 mbar for 1 h. The residue was taken up into H_2_O (10 mL), and the pH value was carefully adjusted to approximately 6.0–7.0 by the dropwise addition of 3% NH_3_. The resulting precipitate was collected by filtration and washed with H_2_O (3 × 5 mL), followed by acetone (3 × 10 mL) to afford *m*-FET (112 mg, 69%; total yield of 63% over two steps) as an off-white solid. If the pH adjustment resulted in partial reformation of **5** (as indicated by a red coloration of the acetone washes and confirmed by TLC), the acetone was concentrated under reduced pressure and the residue was taken up into MeOH. The resulting solution was heated to 65 °C, treated with 6 n HCl, and stirred at 65 °C until the color changed from red to light green, as described above. The mixture was then concentrated under reduced pressure, the residue was taken up into H_2_O, and the pH of the solution was adjusted to approximately 6.0–7.0 by the dropwise addition of 3% NH_3_. The resulting precipitate was collected by filtration and washed with H_2_O, followed by acetone, to give a second crop of the title compound.

#### 2.1.8 Preparation of (*S*)-2-Amino-3-(3-hydroxyphenyl)propanoic Acid (*m*-Tyr)

6 n HCl (30 mL) was added dropwise at 65 °C to a stirred solution of **2** (5 g, 7.07 mmol) in MeOH (60 mL), and the reaction mixture was stirred at 65 °C until the color had changed from red to light green (approximately 40 min). The mixture was allowed to cool to ambient temperature and concentrated under reduced pressure. The residue was taken up into H_2_O (30 mL), and the pH value was adjusted to approximately 7.5–8 by the dropwise addition of 3% NH_3_. The resulting suspension was washed with CH_2_Cl_2_ (3 × 20 mL), which was dried and concentrated under reduced pressure to recycle ligand **1**. The remaining aqueous fraction was concentrated under reduced pressure and the residue was taken up into aqueous MeOH (300 mL). Preswollen Amberlite IR-120 in the H^+^ form (150 mL) was then added, and the mixture was stirred for 16 h. The ion-exchange resin was recovered by filtration, washed with H_2_O until the pH of the filtrate reached 5, and treated with 30% NH_3_:MeOH 2:1 (3 × 300 mL). The combined filtrates were concentrated under reduced pressure, the residue was triturated with acetone and Et_2_O, and the resulting precipitate was collected by filtration to afford the title compound (0.59 g, 46%) as a colorless solid. The spectral data of *m*-Tyr were in accordance with the literature.^35^

#### 2.1.9 Preparation of *tert*-Butyl (*S*)-2-[(*tert*-butoxycarbonyl)amino]-3-(3-hydroxyphenyl)propanoate (Boc-*m*-Tyr-O*t*Bu, **6**)

##### 2.1.9.1 (*S*)-2-[(*tert*-Butoxycarbonyl)amino]-3-(3-hydroxyphenyl)propanoic Acid (Boc-*m*-Tyr-OH)^36^

Boc_2_O (3.1 g, 14.20 mmol) was added to a vigorously stirred solution of *m*-Tyr (2 g, 11.04 mmol) in 1 n NaOH (11 mL) and 10% NaHCO_3_ (20 mL). MeOH (and, if necessary, H_2_O) was added until a homogeneous solution was obtained, and the reaction mixture was stirred at ambient temperature for 16 h. The MeOH was removed under reduced pressure, and the resulting solution was diluted with H_2_O (50 mL) and washed with pentane (3 × 30 mL). The aqueous fraction was acidified with solid NaHSO_4_ to pH 2 and extracted with Et_2_O (2 × 50 mL). The combined ethereal fractions were washed with H_2_O (3 × 30 mL) and brine (2 × 30 mL), dried, and concentrated under reduced pressure to afford the title compound (2.82 g, 91%) as a colorless oil. The spectral data of Boc-*m*-Tyr-OH were in accordance with the literature.

##### 2.1.9.2 Boc-*m*-Tyr-O*t*Bu (**6**)^37^

*N*,*N*-Dimethylformamide dineopentylacetal (8.4 mL, 6.96 g, 30.08 mmol) was added dropwise under reflux to a stirred solution of Boc-*m*-Tyr-OH (2.82 g, 10.03 mmol) and anhydrous *tert*-BuOH (13 mL, 10.14 g, 136.81 mmol) in anhydrous toluene (100 mL), and the mixture was stirred under reflux for 16 h. After cooling to ambient temperature, the reaction mixture was washed with 10% NaHCO_3_ (3 × 30 mL), H_2_O (3 × 30 mL), and brine (2 × 30 mL), dried, and concentrated under reduced pressure. The resulting crude product was purified by column chromatography (EtOAc:hexane 1:3) to afford the title compound (2.16 g, 64%, 58% over two steps) as a colorless solid. NMR spectra showed the presence of two rotamers in an approximately 4:1 ratio. Only data for the major rotamer are reported. ^1^H NMR (400 MHz, CDCl_3_) δ 7.13 (t, *J* = 7.9 Hz, 1H), 6.76–6.72 (m, *J* = 2.6 Hz, 1H), 6.70 (d, *J* = 7.3 Hz, 2H), 6.22 (s, 1H), 5.07 (d, *J* = 8.1 Hz, 1H), 4.43 (dd, *J* = 14.1, 6.5 Hz, 1H), 3.07–2.91 (m, 2H), 1.42 (s, 9H), 1.40 (s, 9H). ^13^C NMR (101 MHz, CDCl_3_) δ 171.29, 156.22, 155.51, 138.06, 129.63, 121.66, 116.58, 114.11, 82.38, 80.15, 54.97, 38.55, 28.45, 28.06. MS (ESI) *m*/*z*: [M + H]^+^ calculated for C_18_H_28_NO_5_^+^: 338.20; found: 338.25. HR-MS (EI) *m*/*z*: [M]^•+^ calculated for C_18_H_27_NO_5_^•+^: 337.1884; found: 337.1881.

#### 2.1.10 Preparation of *tert*-Butyl (*S*)-2-[(*tert*-butoxycarbonyl)amino]-3-{3-[2-(tosyloxy)ethoxy]phenyl}propanoate (**7**)

**7** was prepared from **6** (1.0 g, 2.96 mmol), (CH_2_)_2_(OTs)_2_ (1.43 g, 3.85 mmol), and Cs_2_CO_3_ (0.97 g, 2.96 mmol) in anhydrous MeCN (20 mL) using the same procedure as described for preparation of **3**, except that the reaction time at 50 °C after addition of the alkylating agent amounted to 24 h. The crude product was purified by column chromatography (EtOAc:hexane = 1:3) and the fractions containing the pure product were collected into a 500 mL flask. The collected product fractions were then concentrated under reduced pressure, the residue was taken up into *n*-pentane and transferred into a 25 mL pear shaped flask. Finally, all volatiles were removed under reduced pressure to afford the title compound (0.9 g, 56%; contained 15 mol % *n*-pentane according to the ^1^H NMR spectrum) as a colorless oil. ^1^H NMR (400 MHz, CDCl_3_) δ 7.82 (d, *J* = 8.3 Hz, 2H), 7.35 (d, *J* = 8.3 Hz, 2H), 7.16 (t, *J* = 7.8 Hz, 1H), 6.77 (d, *J* = 7.8 Hz, 1H), 6.65 (dd, *J* = 7.8, 1.8 Hz, 1H), 6.62–6.59 (m, 1H), 4.97 (d, *J* = 7.2 Hz, 1H), 4.41 (dd, *J* = 11.9, 7.2 Hz, 1H), 4.37–4.31 (m, 2H), 4.15–4.09 (m, 2H), 3.00 (t, *J* = 11.9 Hz, 2H), 2.45 (s, 3H), 1.41 (s, 9H), 1.39 (s, 9H). ^13^C NMR (101 MHz, CDCl_3_) δ 171.00, 158.13, 155.20, 145.07, 138.22, 133.05, 130.00, 129.47, 128.16, 122.75, 116.03, 112.95, 82.22, 79.85, 68.21, 65.47, 54.87, 38.60, 28.45, 28.08, 21.79. MS (ESI) *m*/*z*: [M + H]^+^ calculated for C_27_H_38_NO_8_S^+^: 536.23; found: 536.22.

#### 2.1.11 Preparation of (*RS*)- and (*S*)-2-Amino-3-{3-[2-(tosyloxy)ethoxy]phenyl}propanoic Acid [(*RS*)-**8** and **8**]

##### 2.1.11.1 (*S*)-2-Amino-3-{3-[2-(tosyloxy)ethoxy]phenyl}propanoic Acid (**8**)

A solution of **7** (0.19 g) in TFA/TIS/H_2_O (95:2.5:2.5; 10 mL) was incubated at ambient temperature for 4 h and concentrated under reduced pressure. The residue was sonicated with Et_2_O (4 × 20 mL) and pentane (2 × 20 mL), followed by drying under reduced pressure to afford the title compound (85 mg, 61%; contained 17 mol % pentane according to the ^1^H NMR spectrum) as a colorless hygroscopic semisolid. Note that the title compound was obtained as a free base instead of the trifluoroacetate salt, as indicated by an extremely low solubility in common polar solvents like H_2_O, MeOH, or DMSO (which is characteristic for phenylalanine analogs as free bases) and confirmed by the absence of signals corresponding to trifluoroacetate in the ESI-MS spectra. ^1^H NMR [400 MHz, 10% TFA in (CD_3_)_2_SO] δ 7.74 (br d, *J* = 16.1 Hz, 1H), 7.24 (d, *J* = 7.3 Hz, 2H), 6.91 (d, *J* = 7.3 Hz, 2H), 6.66 (t, *J* = 7.3 Hz, 1H), 6.47–5.94 (m, 3H), 4.00–3.33 (m, 5H), 2.09–1.90 (m, 2H), 1.85 (s, 3H). ^13^C NMR [101 MHz, 10% TFA (CD_3_)_2_SO] δ 170.59, 158.25, 145.34, 136.55, 132.61, 130.40, 129.98, 127.97, 122.46, 116.06, 113.52, 69.39, 65.57, 53.15 (d, *J* = 6.9 Hz), 35.94, 21.23. MS (ESI) *m*/*z*: [M + H]^+^ calculated for C_18_H_22_NO_6_S^+^: 380.12; found: 380.06.

##### 2.1.11.2 (*RS*)-2-Amino-3-{3-[2-(tosyloxy)ethoxy]phenyl}propanoic Acid [(*RS*)*-***8**]

For the preparation of (*RS*)-**8**, (*RS*)-**7** was prepared from (*RS*)-*m*-Tyr using the same procedures as described for the preparation of **7** and converted to (*RS*)-**8** using the same procedure as described for the synthesis of **8**. The spectroscopic data for (*RS*)-**8** were identical to those for **8**.

#### 2.1.12 Preparation of Methyl (*S*)-2-[(*tert*-butoxycarbonyl)amino]-3-{4-[2-(tosyloxy)ethoxy]phenyl}propanoate (**9**)

Cs_2_CO_3_ (1.21 g, 3.71 mmol) was added to a solution of Boc-Tyr-OMe (1 g, 3.37 mmol) in anhydrous MeCN (100 mL), and the reaction mixture was stirred at 80 °C for 30 min. After cooling to ambient temperature, (CH_2_)_2_(OTs)_2_ (2.0 g, 5.40 mmol) was added, and the mixture was stirred at 80 °C for another 30 min. The resulting suspension was allowed to cool to ambient temperature and the precipitate was removed by filtration. The filtrate was concentrated under reduced pressure and the residue was purified by column chromatography (EtOAc:hexane = 1:2.1; dry loading) followed by RP chromatography on a C_18_ phase [80 g Chromabond C_18_ ec f (Macherey-Nagel, Düren, Germany), 40% MeCN, 2 L, followed by 45% MeCN, 2 L, 100 mL fractions; dry loading] to remove remaining impurities that were not separated by the initial column chromatography. The fractions containing the pure product were collected into a 500 mL flask and concentrated under reduced pressure. The residue was dried at 2 mbar and 40 °C for 4 h, taken up into Et_2_O (10 mL), and transferred into a 50 mL pear shaped flask. Finally, all volatiles were removed under reduced pressure to afford the title compound (1.03 g, 55%; contained 28 mol % Et_2_O according to the ^1^H NMR spectrum) as a yellow oil, which gradually solidified into a colorless solid. ^1^H NMR (400 MHz, CDCl_3_) δ 7.81 (d, *J* = 8.2 Hz, 2H), 7.34 (d, *J* = 8.2 Hz, 2H), 7.00 (d, *J* = 8.6 Hz, 2H), 6.75–6.65 (m, 2H), 4.95 (d, *J* = 7.8 Hz, 1H), 4.59–4.46 (m, 1H), 4.34 (dd, *J* = 13.7, 6.3 Hz, 2H), 4.11 (dd, *J* = 6.3, 4.0 Hz, 2H), 3.70 (s, 3H), 3.00 (qd, *J* = 13.9, 5.9 Hz, 2H), 2.45 (s, 3H), 1.41 (s, 9H). ^13^C NMR (101 MHz, CDCl_3_) δ 172.47, 157.24, 155.20, 145.08, 133.03, 130.45, 129.99, 128.95, 128.15, 114.77, 80.05, 68.23, 65.58, 54.63, 52.32, 37.60, 28.42, 21.78. MS (ESI) *m*/*z*: [M + H]^+^ calculated for C_24_H_32_NO_8_S^+^: 494.18; found: 494.28.

#### 2.1.13 Preparation of Methyl (*S*)-2-amino-3-[4-(2-fluoroethoxy)phenyl]propanoate Hydrochloride (HCl·FET-OMe)

##### 2.1.13.1 Methyl (*S*)-2-[(*tert*-butoxycarbonyl)amino]-3-[4-(2-fluoroethoxy)phenyl]propanoate (**10**)

**10** was prepared from Boc-Tyr-OMe (1 g, 3.37 mmol), 1-bromo-2-fluoroethane (1.0 mL, 1.82 g, 14.41 mmol), and Cs_2_CO_3_ (1.9 g, 5.83 mmol) in anhydrous MeCN (20 mL) using the same procedure as described for preparation of **3**, except that the reaction time at 50 °C after addition of the alkylating agent amounted to 16 h. The crude product was purified by recrystallization from EtOAc/hexane to afford a first crop of the title compound (0.64 g, 56%) as a colorless solid. The mother liquor was concentrated under reduced pressure, and the residue was purified by column chromatography (EtOAc:hexane = 1:2.1) to afford a second crop of the title compound (0.3 g, total yield of 82%). ^1^H NMR (400 MHz, CDCl_3_) δ 7.08–6.99 (m, *J* = 8.5 Hz, 2H), 6.89–6.80 (m, 2H), 4.96 (d, *J* = 6.8 Hz, 1H), 4.83–4.76 (m, 1H), 4.72–4.65 (m, 1H), 4.54 (dd, *J* = 13.6, 6.8 Hz, 1H), 4.26–4.19 (m, 1H), 4.18–4.12 (m, 1H), 3.70 (s, 3H), 3.02 (qd, *J* = 13.6, 5.9 Hz, 2H), 1.41 (s, 9H). ^13^C NMR (101 MHz, CDCl_3_) δ 172.52, 157.65, 155.21, 130.51, 128.80, 114.85, 82.05 (d, *J* = 170.7 Hz), 80.03, 67.24 (d, *J* = 20.7 Hz), 54.65, 52.31, 37.62, 28.42. ^19^F-NMR (376 MHz, CDCl_3_) δ −223.87. MS (ESI) *m*/*z*: [M + H]^+^ calculated for C_17_H_25_FNO_5_^+^: 342.17; found: 342.27.

##### 2.1.13.2 HCl·FET-OMe

**10** (0.54 g, 1.58 mmol) was taken up into 4.8 m HCl in EtOAc (20 mL) prepared according to Nudelman et al.^38,39^ and the mixture was incubated at ambient temperature for 1 h. All volatiles were then removed under reduced pressure, the residue was triturated with Et_2_O, and the solid was collected by filtration to afford the title compound (0.45 g, 100%; 82% over two steps) as a colorless solid. ^1^H NMR [400 MHz, (CD_3_)_2_SO] δ 8.76 (s, 3H), 7.22–7.11 (m, 2H), 6.96–6.84 (m, 2H), 4.82–4.75 (m, 1H), 4.66 (dt, *J* = 15.9, 7.3 Hz, 1H), 4.26–4.22 (m, 1H), 4.20–4.12 (m, 2H), 3.66 (s, 3H), 3.11 (ddd, *J* = 21.4, 14.1, 6.5 Hz, 2H). ^13^C NMR [101 MHz, (CD_3_)_2_SO] δ 169.37, 157.39, 130.60, 126.86, 114.59, 82.16 (d, *J* = 166.5 Hz), 67.01 (d, *J* = 18.9 Hz), 53.35, 52.51, 34.93. ^19^F-NMR [376 MHz, (CD_3_)_2_SO] δ −222.07. MS (ESI) *m*/*z*: [M + H]^+^ calculated for C_12_H_17_FNO_3_^+^: 242.12; found: 242.24.

#### 2.1.14 Preparation of Methyl (*RS*)-2-amino-3-[4-(2-fluoroethoxy)phenyl]propanoate Hydrochloride [HCl·(*RS*)-FET-OMe]

##### 2.1.14.1 Methyl (*RS*)-2-[(*tert*-butoxycarbonyl)amino]-3-[4-(2-fluoroethoxy)phenyl]propanoate [(*RS*)-**10**]

(*RS*)-**10** was prepared from Boc-(*RS*)-Tyr-OMe using the same procedure as described for the preparation of **10**. The spectroscopic data for (*RS*)**-10** were identical to those for **10**.

##### 2.1.14.2 HCl·(*RS*)-FET-OMe

HCl·(*RS*)-FET-OMe was prepared from (*RS*)**-10** using the same procedure as described for the preparation of HCl·FET-OMe. The spectroscopic data for HCl·(*RS*)-FET-OMe were identical to those for HCl·FET-OMe.

#### 2.1.15 Preparation of (*RS*)- and (*S*)-2-Amino-3-[4-(2-fluoroethoxy)phenyl]propanoic Acid [(*RS*)-FET and FET]

##### 2.1.15.1 (*RS*)-2-Amino-3-[4-(2-fluoroethoxy)phenyl]propanoic Acid [(*RS*)-FET]

HCl·(*RS*)-FET-OMe (0.2 g, 0.72 mmol) was taken up into 12 m HCl (30 mL), and the reaction mixture was stirred at 130 °C for 3.5 h before it was concentrated under reduced pressure. The residue was taken up into acetone (30 mL), and the resulting solution was concentrated under reduced pressure (×3). The crude product was purified by RP chromatography on a C_18_ phase [20% MeCN (0.1% TFA); dry loading], and the fractions containing the pure product were concentrated under reduced pressure. The residue was again taken up into acetone (30 mL) and concentrated under reduced pressure (×3). Finally, recrystallization from MeOH/Et_2_O afforded the title compound (0.12 g, 73%) as a colorless solid. Note that the title compound was obtained as a free base instead of the trifluoroacetate salt, as indicated by an extremely low solubility in common polar solvents like H_2_O, MeOH, or DMSO (which is characteristic for phenylalanine analogs as free bases) and confirmed by the absence of signals corresponding to trifluoroacetate in the ESI-MS spectra. ^1^H NMR [400 MHz, 10% TFA in (CD_3_)_2_SO] δ 8.29 (br d, *J* = 14.9 Hz, 1H), 7.18 (d, *J* = 8.4 Hz, 2H), 6.92 (d, *J* = 8.5 Hz, 2H), 4.88–4.58 (m, 2H), 4.30–4.07 (m, 3H), 2.51 (s, 2H). ^13^C NMR [101 MHz, 10% TFA in (CD_3_)_2_SO] δ 170.72 (d, *J* = 3.6 Hz), 158.13, 131.05, 127.28, 115.01, 82.43 (d, *J* = 166.9 Hz), 67.39 (d, *J* = 19.2 Hz), 53.54 (d, *J* = 6.9 Hz), 35.23 (d, *J* = 4.7 Hz). ^19^F-NMR [376 MHz, 10% TFA in (CD_3_)_2_SO] δ −222.46. MS (ESI) *m*/*z*: [M + H]^+^ calculated for C_11_H_15_FNO_3_^+^: 228.11; found: 228.17.

##### 2.1.15.2 (*S*)-2-Amino-3-[4-(2-fluoroethoxy)phenyl]propanoic Acid (FET).^40^

FET was prepared from HCl·FET-OMe using the same procedure as described for the preparation of (*RS*)-FET. The spectroscopic data for (*RS*)**-**FET and FET were identical.

### 2.2 Radiochemistry.

#### 2.2.1 General

No-carrier-added aqueous [^18^F]fluoride ([^18^F]F^–^) was produced via the ^18^O(p,n)^18^F nuclear reaction by bombardment of enriched [^18^O]H_2_O with 16.5 MeV protons using a BC1710 cyclotron (The Japan Steel Works Ltd., Shinagawa, Japan) at the INM-5 (Forschungszentrum Jülich). All radiosyntheses of *m*-[^18^F]FET and [^18^F]FET-OMe were carried out manually in 5 mL Wheaton V-Vials equipped with PTFE-coated wing stir bars. Anhydrous solvents (MeCN, MeOH) were purchased from Sigma-Aldrich (Steinheim, Germany). Anion exchange cartridges (Sep-Pak Accell Plus QMA carbonate plus light cartridges with 46 mg sorbent per cartridge) were obtained from Waters GmbH (Eschborn, Germany), and polymeric-based StrataX cartridges (60 mg) were obtained from Phenomenex (Aschaffenburg, Germany). [^18^F]FET was prepared according to a known procedure^41^ in an AllInOne automated synthesis module (Trasis, Ans, Belgium), which afforded the probe in activity yields of 46 ± 3% within 53 ± 1 min (calculated based on five representative radiosyntheses).

HPLC analyses were carried out on a Dionex Ultimate 3000 HPLC system equipped with a Multokrom C18 AQ 100–5, 250 × 4.6 mm column (CS-Chromatographie Service GmbH, Langerwehe, Germany) and a DAD UV-detector coupled in series with a Berthold NaI detector, giving a time delay of 0.1–0.3 min between the corresponding responses (depending on the flow rate). The identity of radiolabeled products was confirmed by the coinjection of the corresponding nonradiolabeled reference compounds. Isolated yields of the purified radiolabeled products are reported in terms of decay-corrected radiochemical yields (RCYs), as determined from the initial activity on the QMA cartridge and the activity of the radiolabeled product. The system used for the purification of crude products by semipreparative HPLC consisted of a Knauer pump 40P, a Knauer K-2500 UV detector, a Rheodyne 6-port injection valve, a custom-made Geiger-Müller counter, and a Hydro-RP, 250 × 10 mm, 80 Å, 10 μm column (Synergi; Phenomenex LTD, Aschaffenburg, Germany). To determine the enantiomeric purity of *m*-[^18^F]FET and [^18^F]FET, aliquots of the respective isolated fractions from the preparative HPLC were directly analyzed by chiral HPLC [column: Astec Chirobiotic T, 5 μm, 250 × 4.6 mm; eluent: 55% MeOH (0.02% HCO_2_H); flow rate: 1.0 mL/min]. In the case of [^18^F]FET-OMe, the *ee* was estimated based on chiral HPLC after hydrolysis to [^18^F]FET. To this end, the isolated fraction of [^18^F]FET-OMe from the preparative HPLC was diluted with sat. NaHCO_3_ (9 fold volume) and loaded onto a StrataX cartridge (60 mg). The cartridge was washed with H_2_O (10 mL), the [^18^F]FET-OMe was eluted with EtOH (600 μL), and EtOH was removed at 50 °C under reduced pressure in a stream of argon. The residue was taken up into 6 m HCl (500 μL) and stirred at 120 °C for 10 min to afford [^18^F]FET. After neutralization with sat. NaHCO_3_ (pH 6–7) at ambient temperature, an aliquot of the resulting [^18^F]FET solution was analyzed by chiral HPLC [column: Astec Chirobiotic T, 5 μm, 250 × 4.6 mm; eluent: 55% MeOH (0.02% HCO_2_H); flow rate: 1.0 mL/min].

#### 2.2.2 Preparation of *m*-[^18^F]FET

##### 2.2.2.1 From Precursor **3.**

Aqueous [^18^F]F^–^ (0.1–2.0 GBq) was loaded onto a QMA cartridge, the cartridge was washed with anhydrous MeCN (1 mL), and the [^18^F]F^–^ was eluted with a solution of Bu_4_NOTs (4.0 mg, 9.7 μmol) in MeOH (0.5 mL). The solvent was removed at 85 °C for 5 min under reduced pressure in a stream of argon and a solution of radiolabeling precursor **3** (2 mg, 2.2 μmol) in anhydrous MeCN (0.5 mL) was added. The reaction mixture was stirred at 100 °C for 5 min, and the solvent was removed at 100 °C for 5 min under reduced pressure in a stream of argon. 0.5 m HCl (0.5 mL) and EtOH (0.1 mL) were then added, and the resulting mixture was stirred at 125 °C for 10 min to decompose the radiolabeled intermediate [^18^F]**4**. Following the addition of 2 m NaOH (0.4 mL) and EtOH (0.2 mL), the product was purified by semipreparative HPLC [eluent: 10% EtOH (300 mg/L NH_4_OAc); flow rate: 4.5 mL/min; *t*_*R*_ = 9.0–10.5 min]. The RCY of *m*-[^18^F]FET thus obtained amounted to 29 ± 6% (*n* = 3) within a synthesis time of 94 min. The radiochemical purity amounted to 97%.

##### 2.2.2.2 From Precursor **7**

Aqueous [^18^F]F^–^ (0.1–2.0 GBq) was loaded onto a QMA cartridge, the cartridge was washed with MeOH (0.5 mL) and the [^18^F]F^–^ was eluted with a solution of Bu_4_NOTs (4.0 mg, 9.7 μmol) in MeOH (0.5 mL). The solvent was removed at 60 °C for 5 min under reduced pressure in a stream of argon, and a solution of radiolabeling precursor **7** (5.0 mg, 9.3 μmol) in anhydrous MeCN (0.5 mL) was added. The reaction mixture was stirred at 85 °C for 5 min, and the solvent was removed at 85 °C for 5 min under reduced pressure in a stream of argon. 2 m HCl (0.5 mL) and EtOH (0.1 mL) were then added and the resulting mixture was stirred at 100 °C for 10 min to deprotect the radiolabeled intermediate [^18^F]**11**. Following addition of 2 m NaOH (0.4 mL) and EtOH (0.2 mL), the product was purified by semipreparative HPLC [eluent: 10% EtOH (300 mg/L NH_4_OAc); flow rate: 4.5 mL/min; *t*_*R*_ = 9.0–10.5 min] and formulated by dilution of the eluate with 0.9% NaCl. The RCY of *m*-[^18^F]FET thus obtained amounted to 53 ± 8% (*n* = 8) within a synthesis time of 66 min. The radiochemical purity amounted to >98% and the molar activity to 94 GBq/μmol (for 200 MBq *m*-[^18^F]FET).

#### 2.2.3 Preparation of [^18^F]FET-OMe

Aqueous [^18^F]F^–^ (0.1–2.0 GBq) was loaded onto a QMA cartridge, the cartridge was washed with anhydrous MeCN (1 mL), and the [^18^F]F^–^ was eluted with a solution of Bu_4_NOH·30 H_2_O (25 mg, 31 μmol) in MeCN (0.5 mL). The solvent was removed at 85 °C for 5 min under reduced pressure in a stream of argon, and a solution of radiolabeling precursor **9** (4.9 mg, 9.9 μmol) in anhydrous MeCN (0.5 mL) was added. The reaction mixture was stirred at 85 °C for 10 min, and the solvent was removed at 85 °C for 5 min under reduced pressure in a stream of argon. Trifluoroacetic acid (TFA, 50 μL) was then added, and the resulting mixture was incubated at room temperature for 1 min to deprotect the radiolabeled intermediate [^18^F]**10**. Following addition of MeCN (100 μL) and H_2_O (1 mL), the product was purified by semipreparative HPLC [eluent: 15% MeCN (0.1% TFA); flow rate: 4.5 mL/min; t_R_ = 13.5–15.0 min]. The product fraction was diluted with saturated NaHCO_3_ (9 fold volume) and loaded onto a SPE cartridge (Strata-X RP, 60 mg). The cartridge was washed with H_2_O (10 mL), and the product was eluted with EtOH (1 mL). The solvent was removed under reduced pressure in a stream of argon at 85 °C, and the product was dissolved in 0.9% NaCl (500 μL). The RCY of [^18^F]FET-OMe thus obtained amounted to 41 ± 5% (*n* = 8) within a synthesis time of 90 min. The radiochemical purity amounted to >98% and the molar activity to 156 GBq/μmol (for 710 MBq [^18^F]FET-OMe).

#### 2.2.4 pH Studies with Different Elution Salts

To estimate how [^18^F]F^–^ elution and subsequent heating during the radiofluorination reactions affected the pH value, solutions of Bu_4_NOH·30 H_2_O (25 mg) or Bu_4_NOTs (4 mg) in H_2_O (500 μL) were passed (from the female to the male side) through QMA carbonate cartridges that had either been preconditioned with H_2_O (2 mL) only or with 0.05 m NaHCO_3_ (10 mL) followed by H_2_O (10 mL). The resulting solutions were collected in V-vials, stirred at 85 °C for 10 min, and then cooled to ambient temperature. All pH measurements were performed before and after elution of the QMA cartridges and after the heating step (*n* = 3 for each experimental condition).

### 2.3 Biological Evaluation.

#### 2.3.1 Cell Culture

Human U87 MG glioblastoma cells were purchased from the American Type Culture Collection (ATCC) and cultured under normal growth conditions (37 °C and 5% CO_2_) in minimum essential medium GlutaMAX (MEM, Gibco 41090 028, Fisher Scientific GmbH, Schwerte, Germany) supplemented with 10% fetal bovine serum (FBS, Sigma-Aldrich F2442, Merck KGaA, Darmstadt, Germany), 1% penicillin/streptomycin (Gibco 115140 122), 1% nonessential amino acids (NEAA, Gibco 11140 050), 1% human recombinant insulin (Sigma-Aldrich 91077C), and 1% sodium pyruvate (ThermoFisher 11360 070, Fisher Scientific GmbH, Schwerte, Germany). The cells were grown in cell-culture dishes (ThermoFisher 150350, F 100 mm) with 9 mL culture medium and routinely passaged every 4–5 days when they had reached 80–90% confluency. For the cellular uptake and inhibition studies, cells were seeded into 12-well plates (2 × 10^5^ cells in 1 mL medium/well) 48 h before the start of the experiments.

#### 2.3.2 Cellular Uptake Experiments

Two hours before the start of the experiments, the culture medium was carefully aspirated, the cells were washed with phosphate-buffered saline (PBS, 1 mL, Gibco 10 010 023), and a dye exclusion test with trypan blue (Sigma-Aldrich T 8154) was performed to determine cell viability and the exact cell count (cell viability was always >95%). The tracer solutions were prepared in FBS- and amino acid-free Earle's balanced salt solution (EBSS) at a concentration of 150 kBq/mL. PBS was removed from the wells, and the tracer solution was added (1 mL/well). The cells were then incubated at 37 °C for 60 min, washed twice with ice-cold PBS (1 mL), trypsinized, and harvested. The accumulated radioactivity was measured on an automatic gamma counter (Hidex AMG version 1.4.4, Turku, Finland). Each experiment was conducted at least in triplicate.

#### 2.3.3 Protein Incorporation Studies

To determine the degree of protein incorporation, U87 MG cells were incubated with *m*-[^18^F]FET at 37 °C for 60 min, the tracer solution was removed, and the cells were trypsinized and harvested as described in Section 2.3.2. After centrifugation for 5 min at 2500 × g and 4 °C, the cell pellet was resuspended in 1 mL of TRIS-EDTA buffer (Sigma-Aldrich 93302) and homogenized with a disperser (Ultra-Turrax, Proxxon, Wecker, Luxembourg) at the highest level for 1 min at 4 °C. The resulting homogenate was centrifuged for 30 min at 18400 × g and 4 °C, and the supernatant was loaded onto a PD 10 cartridge (VWR International GmbH, Darmstadt, Germany; preconditioned with 25 mL EBSS). The cartridge was then eluted with 20 mL of EBSS and consecutive 1 mL fractions were collected and measured with a gamma counter to determine the amount of radioactivity in the high- and low-molecular-weight fractions.

#### 2.3.4 Cellular Inhibition Experiments

For the inhibition experiments, U87 MG cells were used and cultured as described above. The following inhibitors, obtained from Sigma-Aldrich, were used: 2-(methylamino)-2-methylpropionic acid (MeAIB) for system A, l-serine for system ASC, and 2-aminobicyclo[2,2,1]heptane-2-carboxylic acid (BCH) for system L. The inhibitors were diluted with EBSS to give the desired final concentrations (1.5 mM, 15 mM, or 150 mM) and added together with the tracer. After incubation for 60 min at 37 °C, the cells were processed, and the uptake of radioactivity measured, as described in Section 2.3.2.

#### 2.3.5 Experimental Animals

All animal studies were conducted in accordance with the EU directive 2010/63/EU for animal experiments and the German Animal Welfare Act (TierSchG, 2006) and were approved by the regional authorities (LANUV, NRW; 84–02.04.2017.A288). In total, 6 immunodeficient male Rowett nude rats (Crl:NIH-*Foxn1*^rnu^, Charles River; 197–359 g body weight) were used for the orthotopic glioblastoma model. The animals were housed in groups of up to 5 animals in individually ventilated cages (NexGen Ecoflo, Rat1800; Allentown Inc., Allentown, NJ, USA) under controlled ambient conditions (22 ± 1 °C and 55 ± 5% relative humidity) and on a 12 h light/dark schedule (lights on from 9:00 p.m. to 9:00 a.m.). Food (Altromin 1324 maintenance diet for rats and mice) and water were provided *ad libitum*. The health status of the animals was monitored daily and showed no changes throughout the experiments.

#### 2.3.6 Orthotopic U87 MG Glioblastoma Xenograft Rat Model

For induction of the orthotopic glioblastoma model, rats were anesthetized with isoflurane (5% for induction and 3–4% for maintenance) in O_2_/air (3:7), and 10^5^ U87 MG cells in 3 μL Matrigel were stereotactically implanted into the brain. To this end, the skin was incised, the periosteum was removed, and a small trepanation (one mm in diameter) was drilled 0.5 mm anterior and 2.5 mm lateral from Bregma. The tumor cells were then injected at a depth of 4.5 mm with a 10 μL Hamilton syringe equipped with a 28G needle, which was left *in situ* for at least 10 min before and 10 min after application to prevent the liquid from escaping along the puncture channel. Following slow (1 mm/min) retraction of the syringe, the drill hole was closed with bone wax, the wound was treated with povidone-iodine (Betaisodona), and the skin was closed by sutures. Before wound closure, intraoperative infiltration analgesia was performed by infiltrating the skin and periosteum with a mixture (50:50) of 0.5% lidocaine and 0.25% bupivacaine in isotonic saline. In addition, the rats were given subcutaneous injections of carprofen (5 mg/kg in 0.1 mL/kg isotonic saline) 30 min prior to the start of the implantation (for preemptive analgesia) and on the following 3 days (for postoperative analgesia). To monitor tumor growth and determine the size and exact location of the intracranial tumors, MRI scans were performed 1, 2, and 3 weeks after tumor cell implantation. The measurements were performed under inhalation anesthesia with isoflurane as described above using a 3T Achieva MRI scanner (Philips Healthcare, Best, The Netherlands) in combination with an 8-channel volumetric rat array (Rapid Biomedical GmbH, Rimpar, Germany). Three-dimensional T2-weighted MR images were acquired using a turbo-spin echo sequence with repetition time = 14 s, echo time = 30 ms, field of view = 60 × 60 × 60 mm^3^, and voxel size = 0.5 × 0.5 × 0.5 mm^3^.

#### 2.3.7 PET Measurements

For comparison of the new tracers with [^18^F]FET, the tumor-bearing rats were divided into two groups (*n* = 3 per group), and each group was measured with [^18^F]FET and either *m*-[^18^F]FET or [^18^F]FET-OMe. Prior to the PET measurements, rats were anesthetized with isoflurane (5% for induction and 1.5–2.5% for maintenance) in O_2_/air (3:7), placed in an animal holder (Minerve, Esternay, France), and fixed with a tooth bar in a respiratory mask. The respiratory rate was monitored with a pressure sensor placed under the animals and maintained at around 40–60 breaths per minute by adjusting the isoflurane concentration. Core body temperature was maintained at about 37 °C by warm air flow through the animal bed. Dynamic PET scans in list mode were performed with a Focus 220 micro-PET scanner (CTI-Siemens, Knoxville, TN, USA) with a resolution at the center of the field of view of 1.4 mm. Data acquisition started with tracer injection (49–65 MBq in 0.5 mL i.v.), proceeded for 120 min, and was followed by a 10 min transmission scan with a ^57^Co point source. After full 3D rebinning, data were reconstructed using an iterative OSEM3D/MAP procedure with attenuation and decay correction^42^ in two different ways: (1) 28 frames (2 × 1 min, 2 × 2 min, 6 × 4 min, 18 × 5 min) for the compilation of regional time activity curves (TACs), and (2) 4 frames (4 × 30 min) for visual display. The resulting voxel sizes were 0.38 mm × 0.38 mm × 0.80 mm. Data analysis was performed using the software VINCI.^43^ Standardized uptake values normalized by body weight (SUV_bw_) were determined by dividing each image by the injected dose and multiplying the result by body weight times 100. To obtain TACs, an elliptical volume of interest (VOI) was placed over the tumor, and the mean SUV_bw_ values were extracted from each of the 28 frames and then plotted over time.

## 3 Results

### 3.1 Synthesis of Radiolabeling Precursors and Nonradioactive Reference Compounds

**Scheme 1:**
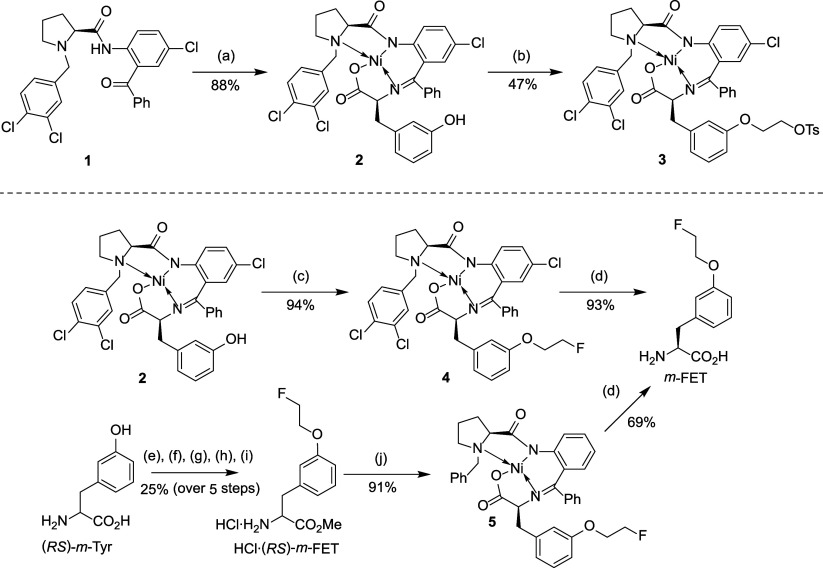
Preparation of Radiolabeling Precursor **3** for *m*-[^18^F]FET (Top) and Reference Compounds *m*-FET and HCl·(*RS*)-*m*-FET (Bottom) Conditions: (a) (*RS*)-*m*-Tyr, Ni(OAc)_2_·4 H_2_O, K_2_CO_3_, MeOH, 60 °C, 72 h; (b) (I) Cs_2_CO_3_, MeCN, 70 °C, 1 h; (II) (CH_2_)_2_(OTs)_2_, 50 °C, 48 h; (c) (I) Cs_2_CO_3_, MeCN, 70 °C, 1 h; (II) 1-bromo-2-fluoroethane, 50 °C, 5 h; (d) HCl, aq. MeOH, 65 °C, 40 min; (e) SOCl_2_, MeOH, 0 → RT, 18 h; (f) Boc_2_O, NaHCO_3_, MeOH, 16 h; (g) (I) Cs_2_CO_3_, MeCN, 70 °C, 1 h; (II) 1-fluoro-2-iodoethane, 50 °C, 16 h; (h) 1 m NaOH, MeOH, 16 h; (i) HCl in EtOAc; (j) (*S*)-BPB, Ni(OAc)_2_·4 H_2_O, K_2_CO_3_, MeOH, 50 °C, 6 h.

Initially, we intended to produce *m*-[^18^F]FET by the application of radiolabeling precursor **3** with a Ni-BPB moiety as a double protecting group (for selected examples of this strategy, see^44−47^). To this end, racemic *m*-Tyr was used for the preparation of the enantiomerically and diastereomerically pure Ni-complex **2** according to the protocol of Nian et al.^29^ (Scheme 1). **2** was then alkylated with (CH_2_)_2_(OTs)_2_ using Cs_2_CO_3_ as a base to afford the desired radiolabeling precursor **3** in 41% yield over two steps.

To prepare the reference compound *m*-FET, **2** was alkylated with 1-bromo-2-fluoroethane, and the resulting intermediate **4** was decomposed using HCl in aqueous MeOH, which furnished *m*-FET in 87% yield over two steps (Scheme 1). Alternatively, esterification of (*RS*)-*m*-Tyr to (*RS*)-*m*-Tyr-OMe followed by *N*-Boc protection,^33^ alkylation with 1-fluoro-2-iodoethane, and two-step deprotection were used to prepare racemic HCl·(*RS*)-*m*-FET in 25% yield over five steps. Subsequent retro-racemization of HCl·(*RS*)-*m*-FET according to the protocol of Nagaoka et al.^48^ via Ni-BPB complex **5** afforded *m*-FET in 60% yield over two steps (Scheme 1).

**Scheme 2:**
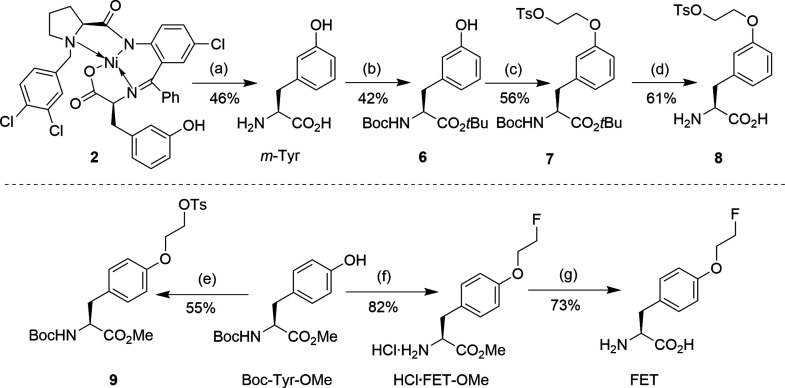
Preparation of Radiolabeling Precursor **7** for *m*-[^18^F]FET (Top), Radiolabeling Precursor **9** for [^18^F]FET-OMe (Bottom Right) and Reference Compound HCl·FET-OMe (Bottom Left) Conditions: (a) (I) HCl, aq. MeOH, 65 °C, 40 min; (II) IRA-120; (b) (I) Boc_2_O, NaHCO_3_, MeOH, 16 h; (II) *N*,*N*-dimethyl-1,1-bis(neopentyloxy)methanamine, *t*BuOH, toluene, 110 °C, 16 h; (c) (I) Cs_2_CO_3_, MeCN, 70 °C, 1 h; (II) (CH_2_)_2_(OTs)_2_, 50 °C, 48 h; (d) TFA:TIS:H_2_O (95:2.5:2.5), 4 h; (e) (I) Cs_2_CO_3_, MeCN, 80 °C, 30 min; (II) (CH_2_)_2_(OTs)_2_, 80 °C, 30 min; (f) (I) Cs_2_CO_3_, MeCN, 70 °C, 1 h; (II) 1-bromo-2-fluoroethane, 50 °C, 16 h; (III) HCl in EtOAc; (g) 12 m HCl, 130 °C, 3 h.

Due to the limited stability of precursor **3** (see Section 3.2.1), the alternative radiolabeling precursor **7** for *m*-[^18^F]FET was also prepared from **2** as follows (Scheme 2). Acidolytic decomposition of **2** into *m*-Tyr was followed by *N*-Boc and CO_2_-*t*Bu protection to give Boc-*m*-Tyr-O*t*Bu (**6**). The latter was then alkylated with (CH_2_)_2_(OTs)_2_ to afford radiolabeling precursor **7** in 14% yield over four steps. To determine the enantiomeric purity of the radiolabeling precursor (see Section 3.2.3), **7** was deprotected with TFA to furnish the corresponding amino acid **8** in 63% yield. Racemic (*RS*)-**8** was prepared in an analogous manner, starting from commercially available (*RS*)-*m*-Tyr.

The radiolabeling precursor **9** for [^18^F]FET-OMe was prepared by alkylation of commercially available Boc-Tyr-OMe with ethylene ditosylate in 55% yield (Scheme 2). For preparation of the corresponding reference compound, Boc-Tyr-OMe was instead alkylated with 1-bromo-2-fluoroethane, followed by deprotection of the resulting intermediate with HCl in EtOAc to obtain HCl·FET-OMe^49^ in 82% yield over two steps (Scheme 2). Racemic HCl·(*RS*)-FET-OMe was prepared in an analogous manner starting from commercially available Boc-(*RS*)-Tyr-OMe. FET^40^ and (*RS*)-FET for determination of the enantiomeric purity of [^18^F]FET-OMe (see Section 3.2.3) were prepared by hydrolysis of HCl·FET-OMe or HCl·(*RS*)-FET-OMe with 12 m HCl at 130 °C for 3 h, which afforded the desired amino acids in 67% and 70% yield, respectively.

### 3.2 Radiotracer Production.

#### 3.2.1 Radiosynthesis of *m*-[^18^F]FET

**Scheme 3:**
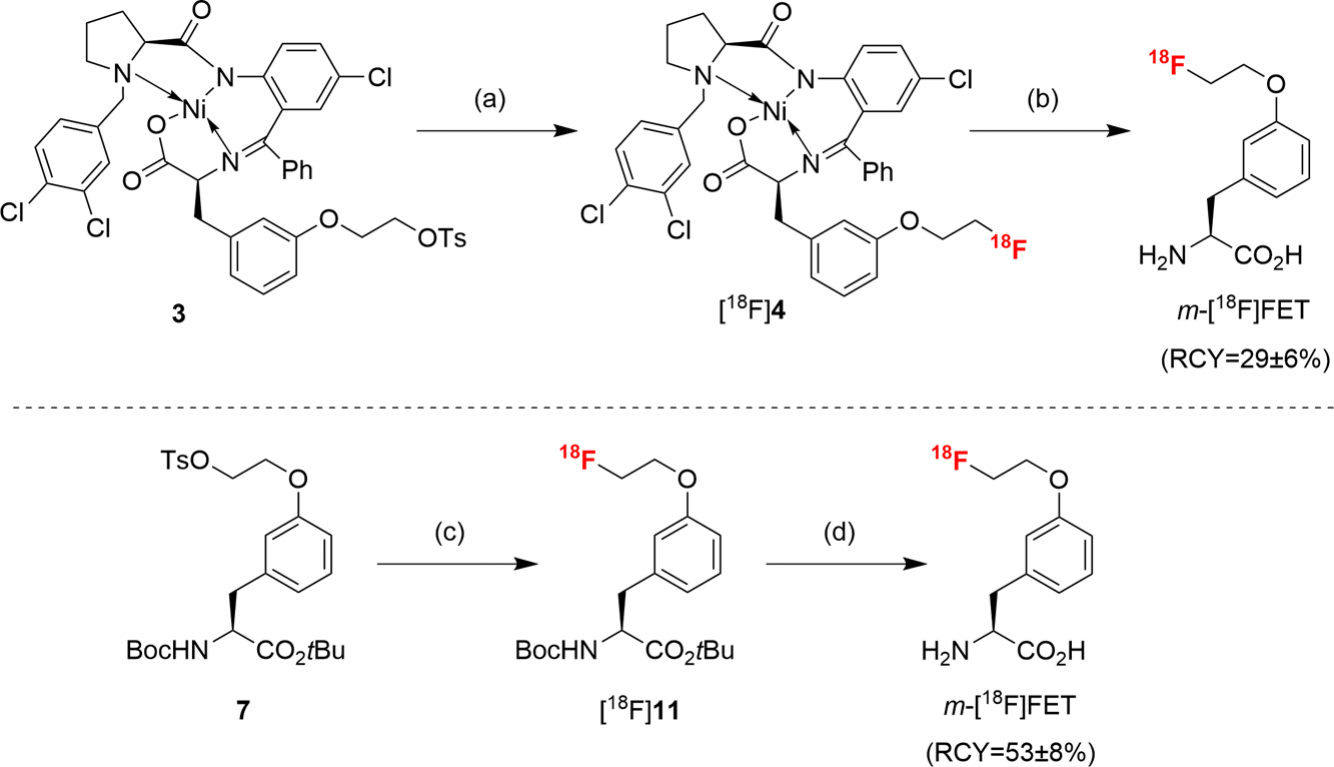
Radiosynthesis of *m*-[^18^F]FET from Ni-BPB Complex **3** (Top) or Precursor **7** (Bottom) Conditions: (a) Bu_4_NOTs, [^18^F]F^–^, MeCN, 100 °C, 10 min; (b) 0.5 m HCl, 125 °C, 10 min; (c) Bu_4_NOTs, [^18^F]F^–^, MeCN, 85 °C, 5 min; (d) 0.83 m HCl in 20% EtOH, 100 °C, 10 min.

The radiosynthesis of *m*-[^18^F]FET was initially accomplished by a one-pot, 2-step procedure using precursor **3** (Scheme 3). To this end, aqueous [^18^F]fluoride ([^18^F]F^–^) was trapped on a QMA anion exchange cartridge and eluted with Bu_4_NOTs (4.0 mg, 9.6 μmol) in MeOH. MeOH was removed under reduced pressure in a stream of argon, and a solution of **3** (2.0 mg, 2.2 μmol) in MeCN was added to the residue. The reaction mixture was then heated at 100 °C for 5 min to afford the radiolabeled complex [^18^F]**4**, which was decomposed with 0.5 m HCl at 125 °C for 10 min. After HPLC purification and formulation, this protocol afforded *m*-[^18^F]FET in radiochemical yields (RCYs) of 29 ± 6% (*n* = 3) and with a radiochemical purity (RCP) of 97% within 94 min. Unfortunately, compound **3** exhibited limited stability under ambient conditions, and the decomposition products adversely affected the efficiency of ^18^F-incorporation. Accordingly, *m*-[^18^F]FET was also prepared from the more stable radiolabeling precursor **7** as follows: Aqueous [^18^F]F^–^ was trapped on an anion exchange cartridge and eluted with a solution of Bu_4_NOTs (4.0 mg, 9.7 μmol) in MeOH (0.5 mL) (Scheme 3). The solvent was removed at 85 °C under reduced pressure in a stream of argon, and the residue was taken up into a solution of **7** (5.0 mg, 9.3 μmol) in anhydrous MeCN. The reaction mixture was then heated at 85 °C for 5 min to afford the radiolabeled intermediate [^18^F]**11**, which was deprotected with 0.83 m HCl in 20% EtOH. Subsequent purification by semipreparative HPLC and formulation by dilution of the eluate with isotonic saline afforded *m*-[^18^F]FET in RCYs of 53 ± 8% (*n* = 8) and an RCP of >98% within 66 min. The molar activity amounted to 94 GBq/μmol (for 200 MBq *m*-[^18^F]FET).

#### 3.2.2 Radiosynthesis of [^18^F]FET-OMe

**Scheme 4:**
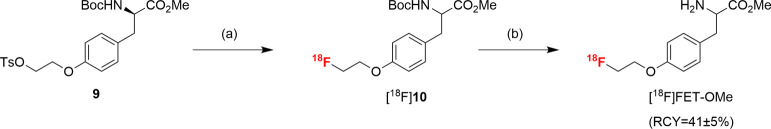
Radiosynthesis of [^18^F]FET-OMe from Precursor **9** Conditions: (a) Bu_4_NOH·30 H_2_O, [^18^F]F^–^, MeCN, 85 °C, 10 min; (b) TFA, 1 min.

For preparation of [^18^F]FET-OMe, **9** was radiofluorinated using the same procedure as described above for **7**, except that [^18^F]F^–^ was eluted with a solution of Bu_4_NOH·30 H_2_O (25 mg, 31 μmol) in MeCN (0.5 mL) (Scheme 4). The resulting intermediate Boc-[^18^F]FET-OMe ([^18^F]**10**) was then deprotected with trifluoroacetic acid (TFA) for 1 min at ambient temperature (Scheme 4). The crude product was purified by semipreparative HPLC and subsequent solid-phase extraction with a Strata-X reversed-phase cartridge. After elution of the product with EtOH, the solvent was removed and the residue was formulated in isotonic saline to afford [^18^F]FET-OMe in RCYs of 41 ± 5% (*n* = 8) and an RCP of 98% within 90 ± 5 min. The molar activity amounted to 156 GBq/μmol (for 710 MBq [^18^F]FET-OMe).

#### 3.2.3 Determination of Enantiomeric Purity

The enantiomeric purity of *m*-[^18^F]FET and [^18^F]FET was determined by HPLC analysis with Astec Chirobiotic T columns, which demonstrated that radiofluorination of **7** using Bu_4_NOTs for [^18^F]F^–^ elution (as described in Section 3.2.1) afforded *m*-[^18^F]FET in an enantiomeric excess (*ee*) of 81% [90.5% (*S*)-isomer]. If basic Bu_4_NOH instead of near-neutral Bu_4_NOTs was applied for the elution of [^18^F]F^–^, *m*-[^18^F]FET was obtained in a lower *ee* of 70% [85% (*S*)-isomer]. In contrast, the *ee* of [^18^F]FET produced from the commercially available *N*-Trt, *O*-*t*Bu-protected tosylate precursor using Bu_4_NOH exceeded 99%. To exclude that the lower *ee* of *m*-[^18^F]FET was related to partial epimerization of the radiolabeling precursor, the enantiomeric purity of **8** (obtained by acidolytic deprotection of precursor **7** as described in Section 3.1) was also determined, which revealed an *ee* of 93% [96.5% (S)-isomer].

Since the enantiomers of HCl·(*RS*)-FET-OMe were inseparable by HPLC with Astec Chirobiotic T or Chirex 3126 d-penicillamine columns, the enantiomeric purity of [^18^F]FET-OMe was determined after its hydrolysis to [^18^F]FET with 6 m HCl at 120 °C for 10 min. If [^18^F]FET-OMe was prepared using Bu_4_NOH for [^18^F]F^–^ elution (as described in Section 3.2.2), the *ee* amounted to 13% [56.5% (*S*)-isomer]. Application of Bu_4_NOTs instead of Bu_4_NOH slightly improved the *ee* to 20% [60% (*S*)-isomer], but led to lower and more inconsistent RCYs.

Direct or indirect determination of the *ee* of radiolabeling precursor **9** proved to be challenging, since neither the enantiomers of **9** nor the enantiomers of the corresponding amino ester methyl *O*-(2-tosylethyl)tyrosinate (prepared by *N*-Boc deprotection of the precursor) could be separated with Astec Chirobiotic T or Chirex 3126 d-penicillamine columns. Furthermore, acidic or basic hydrolysis of methyl *O*-(2-tosylethyl)tyrosinate afforded complex mixtures. Accordingly, the enantiomeric purity of **9** could only be estimated based on the *ee* of FET prepared by the two-step acidolysis of Boc-FET-OMe. The latter was produced analogously to the radiolabeling precursors **7** and **9** by alkylating the cesium phenolate of the appropriate *N*-Boc-protected tyrosine ester. The *ee* of FET thus prepared amounted to 88% [93% (*S*)-isomer], indicating that the precursor synthesis was not associated with extensive epimerization.

To investigate whether anion exchange during [^18^F]F^–^ elution from the anion exchange resin in the CO_3_^2–^/HCO_3_^–^ form with the neutral salt Bu_4_NOTs could increase the basicity of the eluate and, consequently, result in base-induced racemization, the pH-value of aqueous solutions of Bu_4_NOH or Bu_4_NOTs was determined before and after elution of QMA cartridges (Figure S3). The results demonstrated that elution of the QMA cartridges strongly increased the pH value of Bu_4_NOTs solutions from 5.4 ± 0.1 to 8.8 ± 0.1 (*p* < 0.001), while it moderately reduced the pH value of Bu_4_NOH solutions from 12.7 ± 0.0 to 11.4 ± 0.2 (*p* < 0.05). In addition, elution of cartridges that were preconditioned with NaHCO_3_ solution instead of H_2_O according to the protocol of Orlovskaya et al.^50^ resulted in an even more pronounced increase of the pH value of BuNOTs solutions from 5.1 ± 0.1 to 9.5 ± 0.0 (*p* = 0.001). In contrast, heating of the resulting solutions at 85 °C for 5 min had no significant effect on the pH value.

### 3.3 Biological Evaluation.

#### 3.3.1 Cellular Uptake and Protein Incorporation Studies

**Figure 2:**
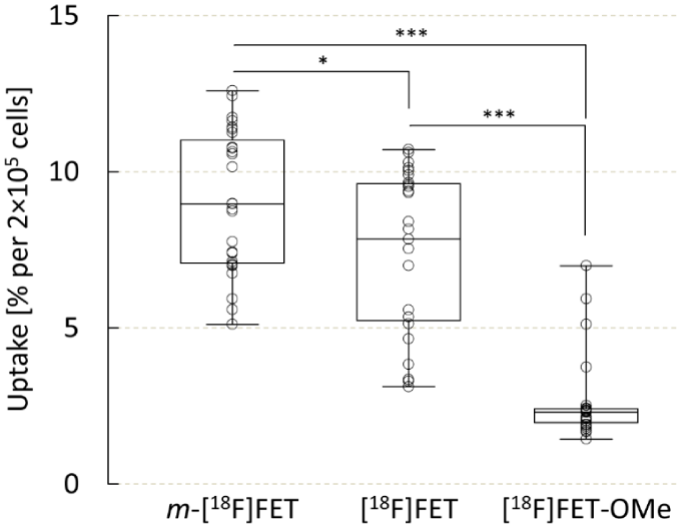
Uptake of *m*-[^18^F]FET, [^18^F]FET, and [^18^F]FET-OMe by human U87 MG glioblastoma cells. Uptake was quantified after incubation of the cells with 150 kBq of the different probes for 1 h and is expressed as the percentage of total activity added per 2 × 10^5^ cells. Boxplots indicate median, 25th and 75th percentiles (box), minimum and maximum values (whiskers), and individual data points (circles). Statistically significant differences between cellular uptake of the probes were identified by Welch’s ANOVA with a Games-Howell posthoc test and are indicated by asterisks (*: *p* < 0.05, ***: *p* < 0.001).

To assess the cellular uptake of the two novel radiolabeled probes, their accumulation in human U87 MG glioblastoma cells was compared with that of the established tracer [^18^F]FET (Figure 2). After incubation for 1 h, cellular uptake of *m*-[^18^F]FET amounted to 9.1 ± 2.2% of the applied activity per 2 × 10^5^ cells and was significantly higher than that of the reference tracer [^18^F]FET (7.4 ± 2.6% per 2 × 10^5^ cells, *p* = 0.034). Conversely, cellular uptake of [^18^F]FET-OMe amounted to only 2.6 ± 1.3% of the applied activity per 2 × 10^5^ cells and was significantly lower than that of both [^18^F]FET (*p* < 0.001) and *m*-[^18^F]FET (*p* < 0.001). To exclude that the higher cellular uptake of *m*-[^18^F]FET compared to [^18^F]FET was related to increased protein incorporation, the soluble fractions obtained by lysis of U87 MG cells incubated with *m*-[^18^F]FET were separated with PD10 columns, which demonstrated complete elution of radioactivity in the low-molecular-weight fraction and no coelution with the protein fraction (see Figure S4).

#### 3.3.2 Competitive Inhibition Studies

**Figure 3:**
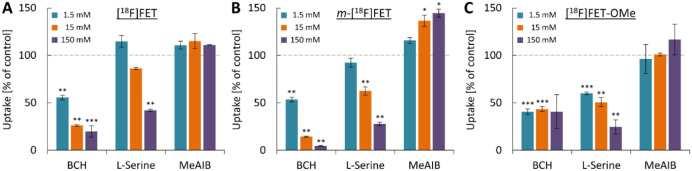
Effect of different amino acid transport inhibitors on uptake of (A) [^18^F]FET, (B) *m*-[^18^F]FET, and (C) [^18^F]FET-OMe by U87 MG glioblastoma cells. Uptake was quantified after incubation of the cells with 150 kBq of the probes for 1 h in the presence of the indicated inhibitor concentrations and is expressed as the percentage of uptake observed in control experiments without inhibitor. The inhibitors used were 2-aminobicyclo[2,2,1]heptane-2-carboxylic acid (BCH) for system L, l-serine for system ASC, and α-(methylamino)isobutyric acid (MeAIB) for system A. Statistically significant differences in uptake compared to control experiments without inhibitors were identified (before normalization) by Welch’s ANOVA with a Games-Howell posthoc test and are indicated by asterisks (*: *p* < 0.05, **: p < 0.01, ***: p < 0.001).

To obtain insight into the amino acid transporters involved in uptake of the different probes, their accumulation in U87 MG cells was also measured in the presence of competitive inhibitors of the main transport systems for neutral amino acids (Figure 3). Inhibition of system L with 2-aminobicyclo[2,2,1]heptane-2-carboxylic acid (BCH) significantly reduced cellular accumulation of [^18^F]FET and *m*-[^18^F]FET in a concentration-dependent manner, with almost complete suppression of *m*-[^18^F]FET uptake at the highest concentration examined (Figure 3A,B). BCH addition also significantly reduced cellular uptake of [^18^F]FET-OMe, but the inhibition showed no evident concentration dependence and did not exceed 60% (Figure 3C). Conversely, inhibition of system ASC with l-serine reduced cellular accumulation of all three probes in a concentration-dependent manner, although the effects of this inhibitor on uptake of [^18^F]FET and *m*-[^18^F]FET were less pronounced than those of BCH and only reached statistical significance at higher inhibitor concentrations. Finally, inhibition of system A with α-(methylamino)isobutyric acid (MeAIB) had no significant effects on the accumulation of [^18^F]FET (Figure 3A) or [^18^F]FET-OMe (Figure 3C), but actually increased cellular uptake of *m*-[^18^F]FET in a concentration-dependent manner (Figure 3B).

#### 3.3.3 *In Vivo* Biodistribution Studies

**Figure 4:**
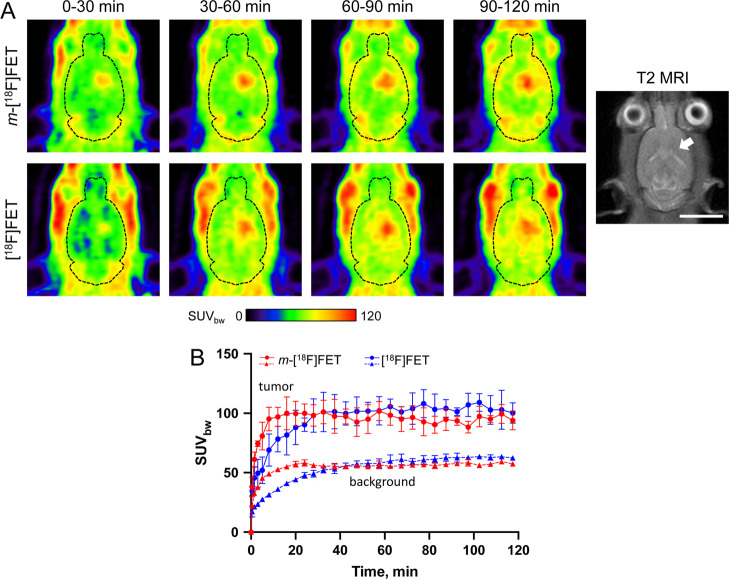
Comparison of *in vivo* tumor uptake of *m*-[^18^F]FET and [^18^F]FET in an orthotopic U87 MG glioblastoma rat model. (A) Representative horizontal PET images (summed over the indicated 30 min time frames) of the same tumor-bearing rat measured (on different days) with *m*-[^18^F]FET (top) or [^18^F]FET (bottom). The outline of the brain is indicated by dashed lines. Also shown is a T2-weigthed MRI of the animal, with the location of the intracerebral tumor indicated by an arrow. Scale bar: 10 mm. (B) Comparison of the mean time-activity curves (*n* = 3) for tumoral and brain (background) accumulation of radioactivity in measurements with *m*-[^18^F]FET (red) and [^18^F]FET (blue).

**Figure 5:**
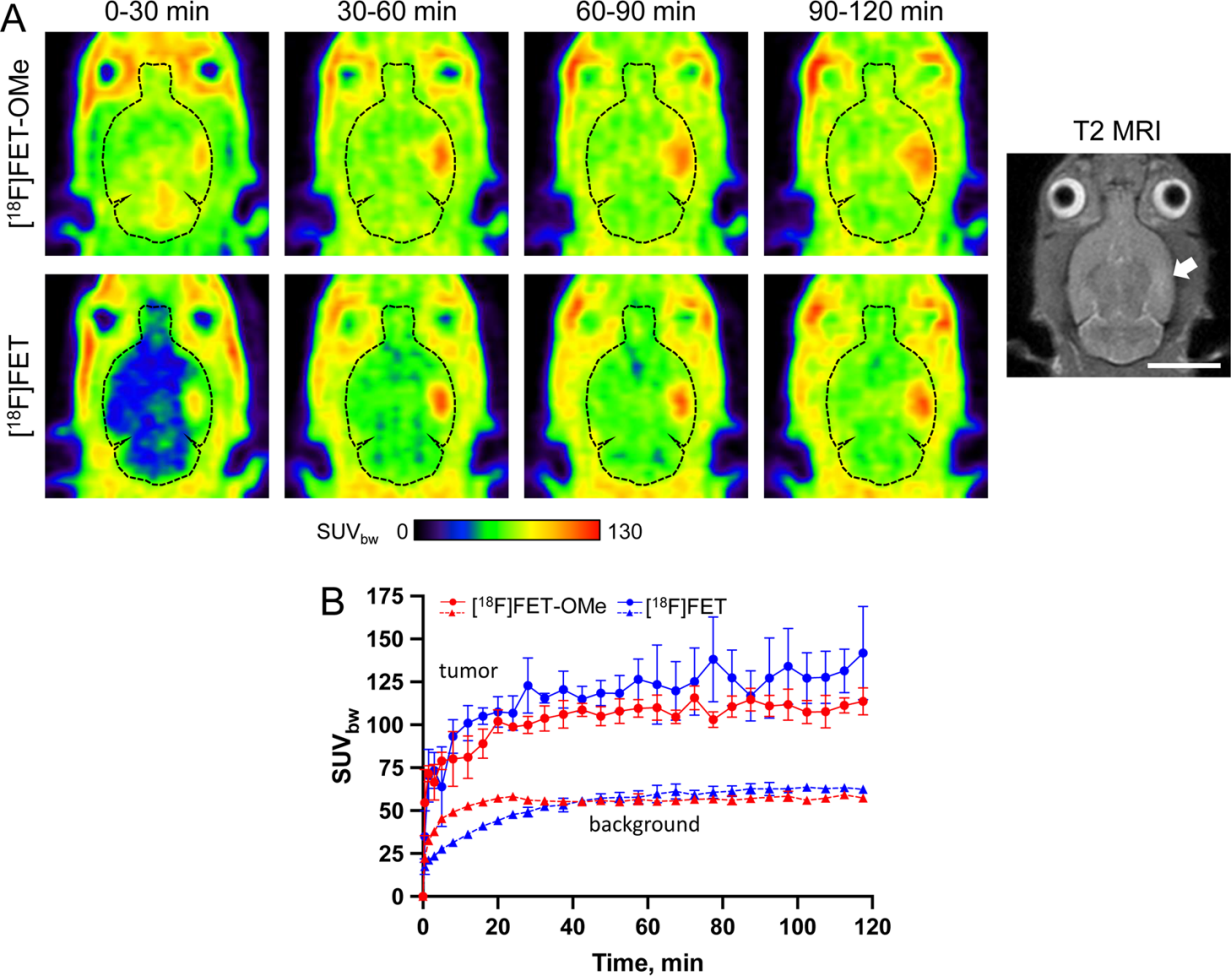
Comparison of *in vivo* tumor uptake of [^18^F]FET-OMe and [^18^F]FET in an orthotopic U87 MG glioblastoma rat model. (A) Representative horizontal PET images (summed over the indicated 30 min time frames) of the same tumor-bearing rat measured (on different days) with [^18^F]FET-OMe (top) or [^18^F]FET (bottom). The outline of the brain is indicated by dashed lines. Also shown is a T2-weigthed MRI of the animal, with the location of the intracerebral tumor indicated by an arrow. Scale bar: 10 mm. (B) Comparison of the mean time-activity curves (*n* = 3) for tumoral and brain (background) accumulation of radioactivity in measurements with [^18^F]FET-OMe (red) and [^18^F]FET (blue).

Next, the *in vivo* imaging properties of the two novel analogs and [^18^F]FET were compared in immunodeficient Rowett nude rats xenotransplanted with intracerebral U87 MG tumors. While the maximal tumor uptake of *m*-[^18^F]FET was similar to that of the reference tracer [^18^F]FET, the *meta*-substituted probe exhibited faster brain and tumor uptake kinetics, which resulted in higher tumoral SUV_bw_ values during the first 30 min p.i. (Figure 4). [^18^F]FET-OMe showed faster brain uptake as well, but the time course of tumoral radioactivity accumulation with this probe was similar to that with [^18^F]FET (Figure 5). In addition, the maximum tumor uptake of radioactivity in measurements with [^18^F]FET-OMe tended to be lower than that in the measurements with [^18^F]FET (Figure 5), although this difference did not reach statistical significance.

## 4 Discussion

In the present work, two novel analogs of the established radiotracer [^18^F]FET were prepared and compared with the parent compound using *in vitro* cellular uptake studies and *in vivo* PET imaging. Both tracers could be prepared in good radiochemical yields (41–56%) within 66–90 min, which is comparable to the radiosynthesis of [^18^F]FET.^51^ Unexpectedly, analysis of enantiomeric purities revealed that radiofluorination of the corresponding *N*-Boc-protected tosylate precursors resulted in almost complete ([^18^F]FET-OMe) or partial (*m*-[^18^F]FET) epimerization of the produced tracers, while preparation of [^18^F]FET from the respective *N*-Trt-protected precursor afforded the enantiomerically pure probe. The latter observation could be rationalized by the assumption that base-induced epimerization of *N*-protected amino esters occurs via abstraction of the α-proton with the formation of a planar carbanion intermediate. In this case, electron-donating arylalkyl *N*-protecting groups like trityl or 5-dibenzosuberyl should suppress racemization by impeding the formation of the carbanion intermediate, possibly in part by steric shielding of the α-CH.^52^ Conversely, electron-withdrawing acyl or urethane *N*-protecting groups like trifluoroacetyl or Boc should stabilize the intermediate and thus favor epimerization. The stronger epimerization of *N*-Boc-protected methyl compared to *tert*-butyl esters could reflect a better accessibility of the α-proton in the former and/or a stronger I^+^ effect of the *tert*-butyl group in the latter compounds.

Noteworthy, the application of *N*-Boc protected precursors for the preparation of [^18^F]FET and its analogs is well documented,^53−57^ but little is known about the enantiomeric purity of the produced tracers. Interestingly, Wang et al.^57^ also observed extensive epimerization of ^18^F-labeled amino acids prepared from *N*-Boc-protected precursors, which could be reduced by replacement of K_2_CO_3_/K2.2.2 with Bu_4_NHCO_3_ to lower the pH during the radiofluorination step. Nevertheless, our own findings indicate that *N*-Boc protection may still not be optimal for the preparation of enantiomerically pure α-amino acids or amino esters via S_n_2 radiofluorination, since even [^18^F]F^–^ elution with the essentially neutral salt Bu_4_NOTs resulted in partial epimerization. As exemplified by our elution experiments with aqueous solutions of Bu_4_NOTs, this can most likely be explained by anion exchange with the carbonate and/or bicarbonate counterions of the anion exchange resin, which results in an increase of the pH that appears to be sufficient for base-induced epimerization.

Importantly, *in vitro* studies with human U87 MG glioblastoma cells revealed significantly higher cellular uptake of *m*-[^18^F]FET compared to [^18^F]FET, despite the lower enantiomeric purity. While our data provide no direct insight into the exact transporters involved in the accumulation of *m*-[^18^F]FET, the uptake of large neutral amino acids by U87 MG cells has been shown to be mainly mediated by LAT1^58^. Moreover, inhibition of system L with BCH significantly reduced cellular uptake of both *m*-[^18^F]FET and (to a lesser extent) [^18^F]FET, which is in line with previous studies demonstrating reduced accumulation of [^18^F]FET after inhibition of system L or knockdown of LAT1.^7,9,13^ Finally, while the binding of amino acids to LAT1 (as measured by *cis*-inhibition assays and *K*_*m*_ values) is typically quite stereoselective for the corresponding (*S*)-enantiomer,^59−62^ LAT1 can also transport (*R*)-amino acids, and the rate of transport (as measured by *trans*-stimulation assays and *V*_max_ values) was found to be much less stereoselective.^60,62^ As such, it seems reasonable to assume that the higher cellular uptake of *m*-[^18^F]FET compared to [^18^F]FET observed in the present study can at least in part be attributed to the fact that *meta*-substituted amino acids are typically better substrates of LAT1 than the corresponding *para*-substituted derivatives.^19−22,24^ However, apart from LAT1, several other amino acid transporters belonging to the systems L, B°, B°^,+^, and ASC, many of which are also present in U87 MG cells,^58^ have been shown or proposed to be (directly or indirectly) involved in tumoral [^18^F]FET uptake.^6−9,13,14^ Indeed, our finding that cellular uptake of both, *m*-[^18^F]FET and [^18^F]FET, was also sensitive to l-serine points to the involvement of system ASC, which is primarily responsible for the transport of small neutral amino acids. While direct transport of the probes by members of this system cannot be excluded, it is also possible that their inhibition indirectly reduced cellular uptake by other systems. Thus, transport of small neutral amino acids by systems like ASC and A has been shown to contribute to the counter-gradients required for uptake of large neutral amino acids by system L, thereby increasing the net accumulation of substrates by transporters like LAT1.^63−65^ Interestingly, coexpression of transporters from systems L and A has been shown to increase the net accumulation of amino acids that are specific system L substrates, while decreasing the net accumulation of amino acids that are substrates for both transport systems.^63^ With this in mind, the paradoxical stimulation of *m*-[^18^F]FET uptake observed after inhibition of system A with MeAIB could indicate that this probe is also a substrate for transport by members of system A.

Another finding of the present study that deserves further investigation is that the maximum tumor uptake of *m*-[^18^F]FET in the orthotopic glioblastoma model was comparable to that of [^18^F]FET, which is in apparent contrast to the higher cellular uptake observed *in vitro*. One possible explanation for this finding could be that LAT1 and other members of system L operate through an exchange mechanism that utilizes the intracellular pool of small neutral amino acids for exchange with large neutral amino acids from the extracellular space. As a consequence, their ability to directly concentrate substrates strongly depends on the counter-gradients of various amino acids across the plasma membrane,^65,66^ which are likely to differ between *in vitro* and *in vivo* conditions. For example, it seems conceivable that the exact concentration gradients present *in vivo* could simply impose an upper limit on the net accumulation of tracers by the tumor cells, resulting in similar tumoral accumulation of [^18^F]FET and *m*-[^18^F]FET despite differences in the transport capacity of both probes. In line with this assumption, tumoral tracer accumulation of *m*-[^18^F]FET during the first 30 min p.i. was indeed faster and more pronounced, suggesting an improved capacity of this analog for transport across the BBB and into the tumor cells. Regardless of the underlying mechanisms, the faster *in vivo* tumor accumulation of *m*-[^18^F]FET compared to [^18^F]FET could have certain practical advantages for clinical PET imaging by, e.g., shortening the necessary scan times in brain tumor patients. Furthermore, given that kinetic analysis of tumoral [^18^F]FET uptake has been shown to provide valuable information for the differential diagnosis and grading of gliomas,^3,67−69^ it remains to be evaluated if the uptake kinetics of *m*-[^18^F]FET are also affected by the exact tumor type or grade. In addition, further studies will be required to evaluate the *in vivo* imaging properties of *m*-[^18^F]FET enantiomers. Indeed, previous results obtained with different enantiomers of [^18^F]FET are contradictory and provide no clear-cut evidence for the superior imaging properties of the (*S*)-enantiomer. For example, compared to (*S*)-[^18^F]FET, brain uptake of (*R*)-[^18^F]FET was reported to be negligible in mice^51^ but 2-fold higher in pigs,^70^ and similar in humans.^70^ Likewise, while (*R*)-[^18^F]FET showed lower tumor accumulation in a subcutaneous tumor model and human brain tumor patients, the tumor-to-background ratios compared to (*S*)-[^18^F]FET were up to 2-fold higher.^70,71^ On the other hand, (*R*)-[^18^F]FET has been reported to exhibit only minor uptake by colon carcinoma or glioma cells *in vitro*(6,57) and (*S*)-FET was a much more effective inhibitor of LAT1 in different expression systems.^70^

While the effects of aromatic substituents on amino acid transport by LAT1 are relatively well established, the consequences of modification of the carboxyl group remain controversial, with some^16,18,25,26^ but not all studies,^24,27,28^ reporting a loss of substrate activity. Our present findings indicate that esterification of the carboxyl group in [^18^F]FET does not completely prevent cellular uptake via the main amino acid transport systems in U87 MG cells *in vitro*. In addition, our results suggest that the contribution of individual amino acid transport systems to *in vitro* uptake of [^18^F]FET-OMe may differ from their contribution to the accumulation of [^18^F]FET. Thus, while the present and previous studies demonstrate that high concentrations of BCH almost completely abolish cellular uptake of [^18^F]FET,^7,9^ uptake of [^18^F]FET-OMe was less sensitive to inhibition of system L with BCH. Given that the protocol applied for the preparation of [^18^F]FET-OMe resulted in almost complete epimerization of the probe, these findings could at least in part reflect the somewhat lower transport rates of (*R*)- compared to (*S*)-amino acids by LAT1. Nevertheless, the reduced but still significant BCH-sensitivity of [^18^F]FET-OMe uptake indicates that LAT1 or other members of system L are still capable of transporting this probe, albeit possibly with a reduced transport capacity. Additionally, the partial sensitivity to l-serine suggests that members of system ASC contribute to the uptake of [^18^F]FET-OMe, either by direct transport or through a functional coupling with other transport systems, as discussed above. In any case, the fact that none of the inhibitors examined completely prevented *in vitro* accumulation of [^18^F]FET-OMe suggests that cellular uptake of this probe either involves multiple transport systems or additional mechanisms, such as passive transfer across the cell membrane. Interestingly, and in contrast to the rather low cellular uptake observed *in vitro*, tumoral uptake of [^18^F]FET-OMe in the orthotopic glioma model was only slightly lower than that of [^18^F]FET. The most likely explanation for this observation is that [^18^F]FET-OMe was rapidly demethylated into [^18^F]FET by endogenous carboxylesterases, which have been shown to catalyze the *in vivo* biotransformation of numerous ester-containing drugs and prodrugs.^72^ However, even though the faster overall brain uptake of radioactivity observed with [^18^F]FET-OMe compared to [^18^F]FET indicates that esterification accelerated brain entry (possibly by enabling rapid passive transfer of the intact tracer across the BBB), there was no beneficial effect on the time-course or magnitude of tumor accumulation. Considering the poor cellular uptake of intact [^18^F]FET-OMe by U87 MG cells *in vitro*, the latter might reflect the low activity of brain esterases, which typically results in much slower ester hydrolysis than in peripheral tissues.^73,74^ Alternatively or in addition, the lower enantiomeric purity of [^18^F]FET-OMe compared to [^18^F]FET might result in reduced tumor uptake through the formation of a mixture of (*S*)- and (*R*)-[^18^F]FET during ester hydrolysis. On the other hand, previous studies indicate that hydrolysis of racemic esters by brain esterases is highly selective for the corresponding (*S*)-enantiomer,^75,76^ which could explain why [^18^F]FET-OMe and [^18^F]FET showed comparable tumor uptake despite a much lower enantiomeric purity of the former probe. Finally, lacking detailed radiometabolite analyses, another but much less likely possibility is that a significant fraction of the tumoral radioactivity accumulation was indeed attributable to intact [^18^F]FET-OMe. However, given that our inhibition studies point to differences in the exact transport systems involved in the uptake of [^18^F]FET-OMe and [^18^F]FET by U87 MG cells, it seems unlikely that both probes would exhibit essentially identical tumoral uptake kinetics. In any case, while firm conclusions regarding the underlying mechanisms would require further studies, our findings indicate that modification of the aromatic side-chain is a more promising strategy for the development of [^18^F]FET analogs with improved transport properties than modification of the carboxylic acid function.

## 5 Conclusion

In summary, two novel [^18^F]FET analogs were prepared with acceptable to good radiochemical yields and subjected to a preclinical evaluation. While esterification of the carboxyl group in [^18^F]FET reduced cellular uptake *in vitro* and provided no advantage for *in vivo* tumor imaging, placement of the [^18^F]fluoroethoxy group in *meta*- instead of *para*-position significantly improved *in vitro* uptake and accelerated tumor accumulation in an orthotopic glioblastoma model. As such, *m*-[^18^F]FET represents a promising alternative to [^18^F]FET for brain tumor imaging and deserves further evaluation with regard to its transport properties and *in vivo* biodistribution.

## Supporting Information Available

^1^H-, ^13^C-, and ^19^F-NMR spectra for all prepared compounds; chiral HPLC chromatograms of racemic and enantiomerically pure *m*-FET, FET, and **8**; HPLC chromatograms of *m*-[^18^F]FET and [^18^F]FET-OMe; details on the determination of carrier amount and molar activity of the probes; results of the elution experiments with Bu_4_NOTs and Bu_4_NOH; results of the protein incorporation studies with *m*-[^18^F]FET (PDF)

## Abbreviations

[^18^F]F^–^: [^18^F]fluoride
[^18^F]FDG: 2-[^18^F]fluoro-2-deoxy-d-glucose
[^18^F]FET: *O*-([^18^F]fluoroethyl)-l-tyrosine
[^18^F]FET-OMe: *O*-([^18^F]fluoroethyl)-l-tyrosine methyl ester
BBB: blood-brain-barrier
BCH: 2-aminobicyclo[2,2,1]heptane-2-carboxylic acid
(CD_3_)_2_SO: dimethyl sulfoxide-d_6_
CDCl_3_: deuterochloroform
D_2_O: deuterium oxide
EBSS: Earle's balanced salt solution
*ee*: enantiomeric excess
HRMS: high resolution mass spectrometry
LRMS: low resolution mass spectrometry
*m*-[^18^F]FET: *O*-([^18^F]fluoroethyl)-l-*meta*-tyrosine
MeAIB: α-(methylamino)isobutyric acid
PBS: phosphate-buffered saline
PET: positron emission tomography
RCP: radiochemical purity
RCY: radiochemical yield
SAR: structure–activity relationship
SUV_bw_: standardized uptake value normalized by body weight
TFA: trifluoroacetic acid
TIS: triisopropylsilane
VOI: volume of interest
