# Supplementary material for: Radiosynthesis and Preclinical Evaluation of m-[18F]FET and [18F]FET-OMe as Novel [18F]FET Analogs for Brain Tumor Imaging
Source: Mol Pharm. 2024 May 15;21(6):2795–812. doi: 10.1021/acs.molpharmaceut.3c01215 (PMC11151201; doi:10.1021/acs.molpharmaceut.3c01215)
Supplement: Supplementary file 1 — mp3c01215_si_001.pdf [file mp3c01215_si_001.pdf]

Supporting Information for

Radiosynthesis and preclinical evaluation of *m*-[^{18}F]FET and [^{18}F]FET-OMe as novel [^{18}F]FET analogs for brain tumor imaging

Benedikt Gröner[1,2§], Chris Hoffmann[1,2§], Heike Endepols[1,2,3], Elizaveta A. Urusova[1,2], Melanie Brugger[1], Felix Neumaier[1,2], Marco Timmer[4], Bernd Neumaier[1,2], Boris D. Zlatopolskiy[1,2]*

[1] *Forschungszentrum Jülich GmbH, Institute of Neuroscience and Medicine, Nuclear Chemistry (INM-5), Wilhelm-Johnen-Straße, 52428 Jülich, Germany.*

[2] *University of Cologne, Faculty of Medicine and University Hospital Cologne, Institute of Radiochemistry and Experimental Molecular Imaging, Kerpener Straße 62, 50937 Cologne, Germany.*

[3] *University of Cologne, Faculty of Medicine and University Hospital Cologne, Department of Nuclear Medicine, Kerpener Straße 62, 50937 Cologne, Germany.*

[4] *University of Cologne, Faculty of Medicine and University Hospital Cologne, Center for Neurosurgery, Department of General Neurosurgery, Kerpener Straße 62, 50937 Cologne, Germany.*

§ B.G. and C.H. contributed equally.

[*] *Corresponding author; e-mail: b.neumaier@fz-juelich.de*

Table of contents

1 NMR spectra S2
  1.1 Compound **2** S2
  1.2 Compound **3** S3
  1.3 Compound **4** S4
  1.4 *m*-FET S6
  1.5 Boc-(*RS*)-*m*-FET-OMe S8
  1.6 Boc-(*RS*)-*m*-FET-OH S10
  1.7 HCl·(*RS*)-*m*-FET S12
  1.8 Compound **5** S14
  1.9 Compound **6** S16
  1.10 Compound **7** S17
  1.11 Compound (*RS*)-**8** S18
  1.12 Compound **9** S19
  1.13 HCl·FET-OMe S20
  1.14 (*RS*)-FET S22
2 HPLC chromatograms S24
  2.1 Quality control HPLC chromatograms of the reference compounds S24
  2.2 Chiral HPLC chromatograms of racemic and enantiomerically pure *m*-FET S29
  2.3 Chiral HPLC chromatograms of racemic and enantiomerically pure FET S30
  2.4 Chiral HPLC chromatogram of **8** S31
  2.5 HPLC chromatograms of *m*-[^{18}F]FET S32
  2.6 HPLC chromatograms of [^{18}F]FET-OMe S35
3 Determination of molar activity of *m*-[^{18}F]FET and [^{18}F]FET-OMe S38
4 Elution experiments with Bu_4NOTs and Bu_4NOH S40
5 Protein incorporation S41

1 NMR spectra

1.1 Compound **2**

^{1}H-NMR spectrum of **2**

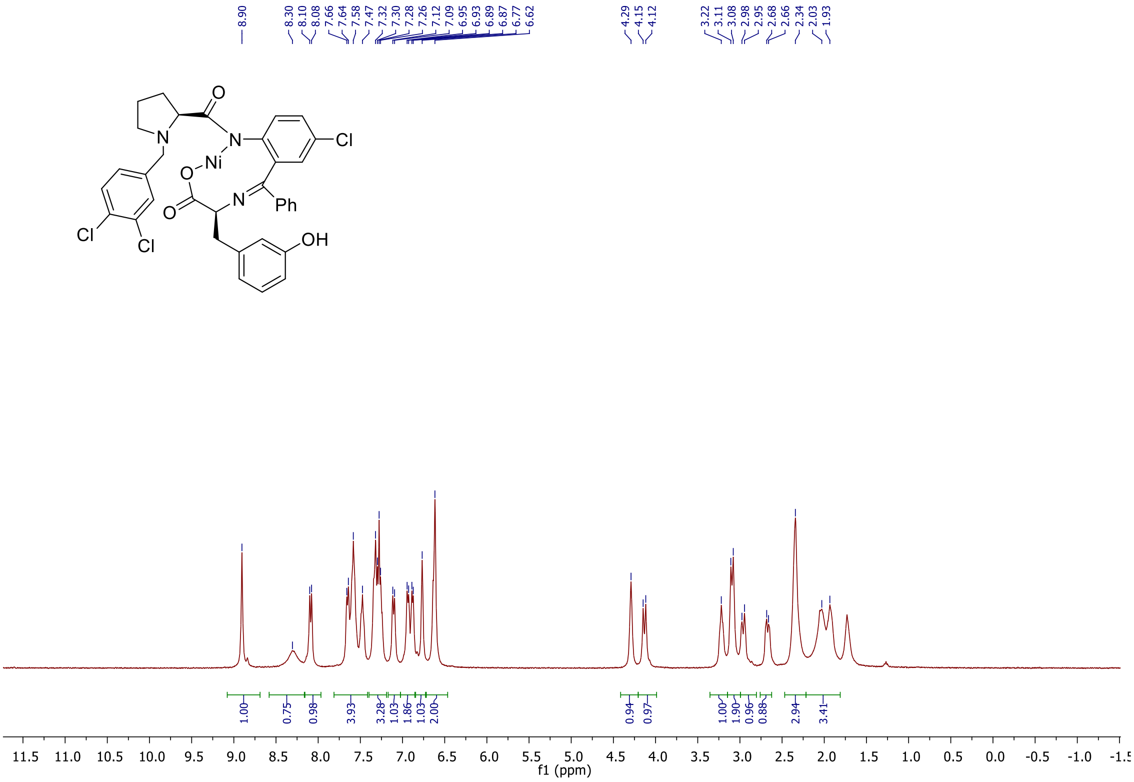

^{13}C-NMR spectrum of **2**

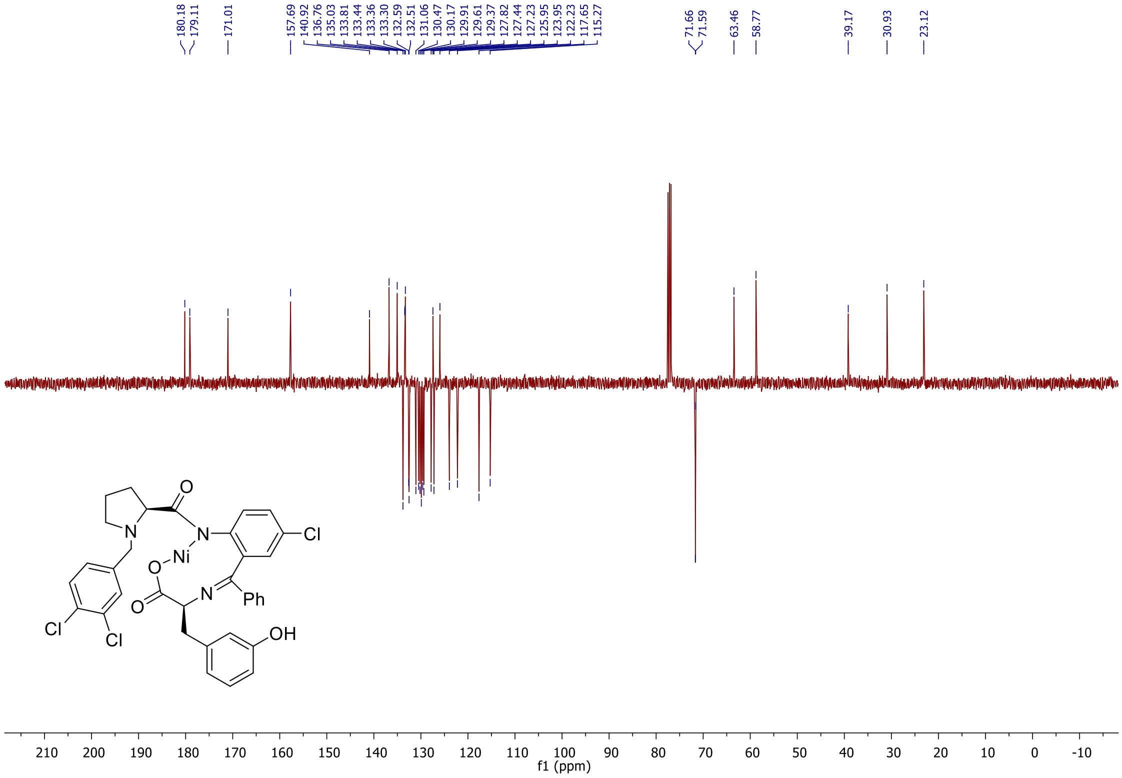

1.2 Compound **3**

^{1}H-NMR spectrum of **3**

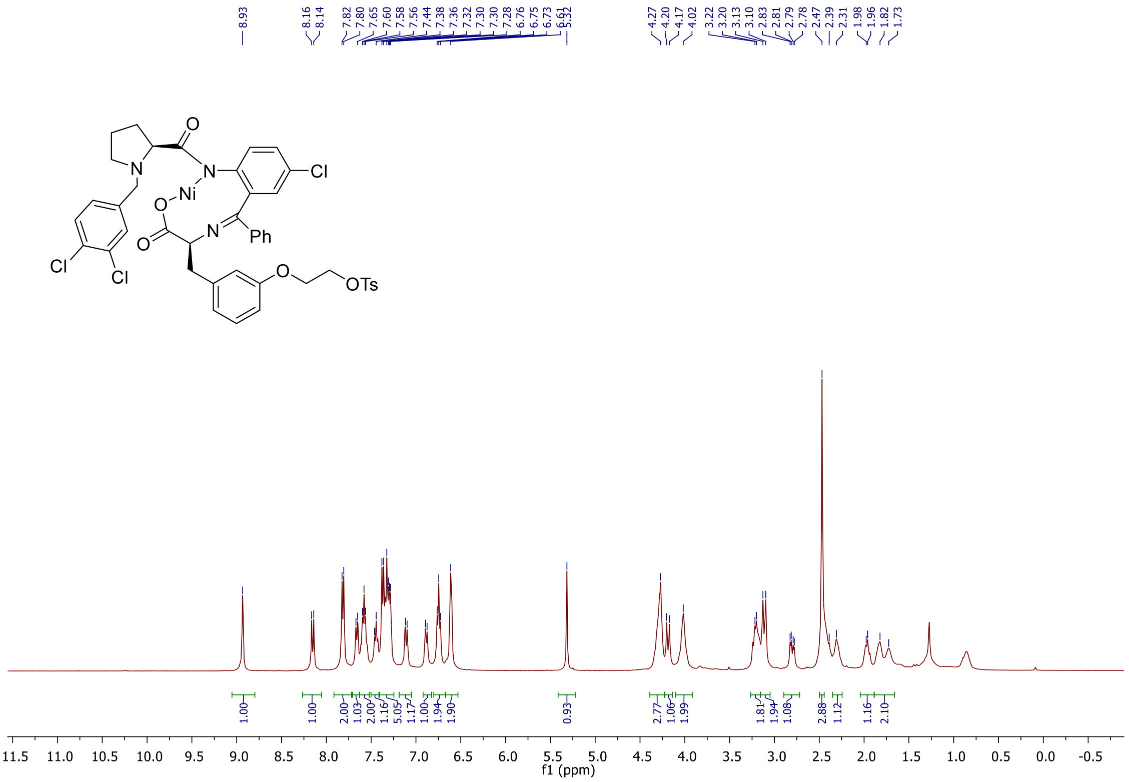

^{13}C-NMR spectrum of **3**

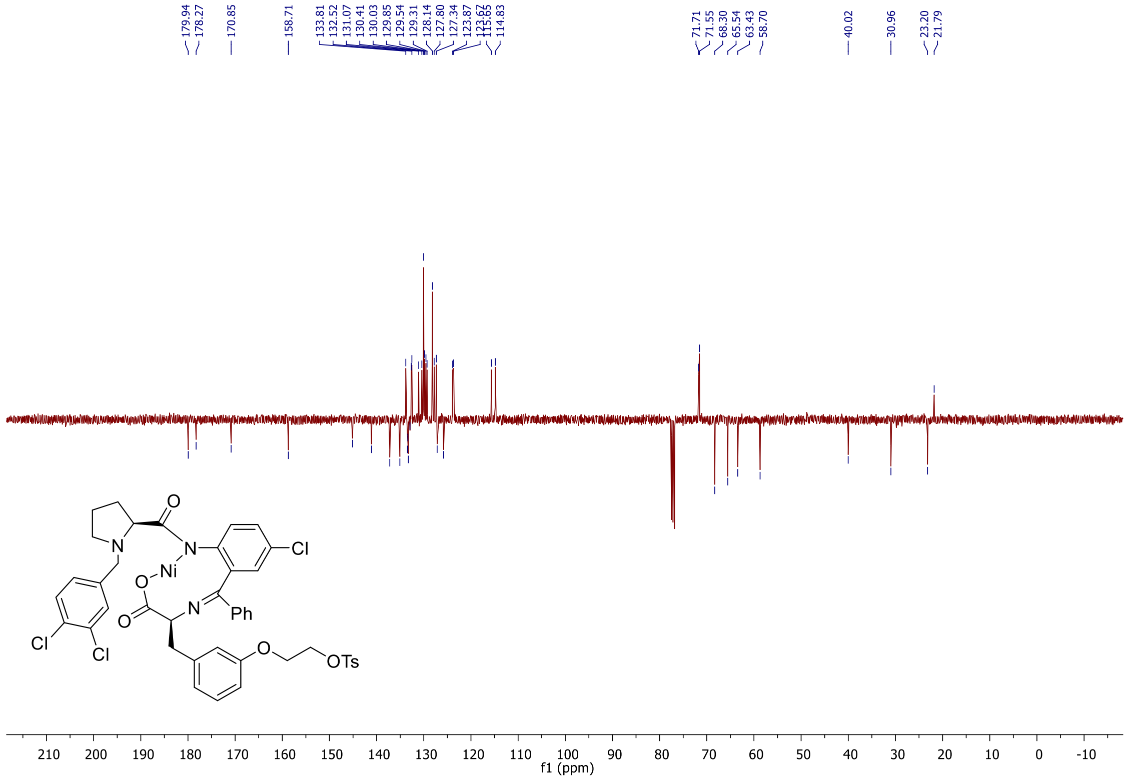

1.3 Compound **4**

^{1}H-NMR spectrum of **4**

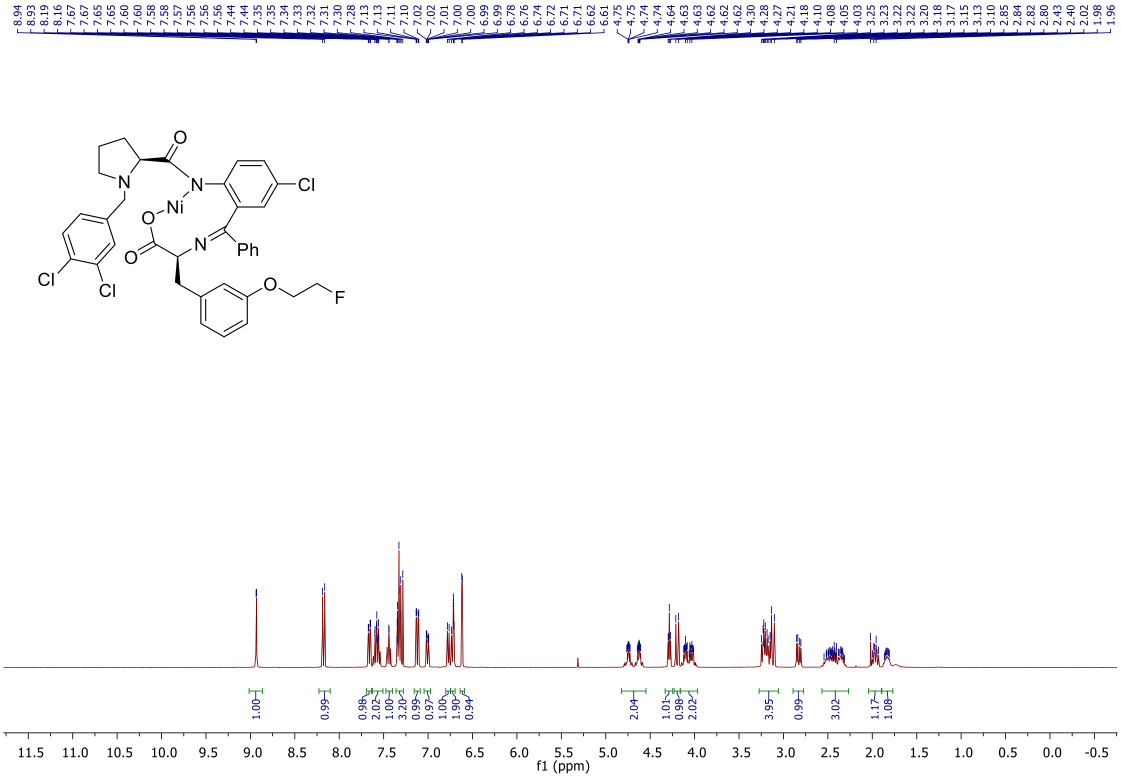

^{13}C-NMR spectrum of **4**

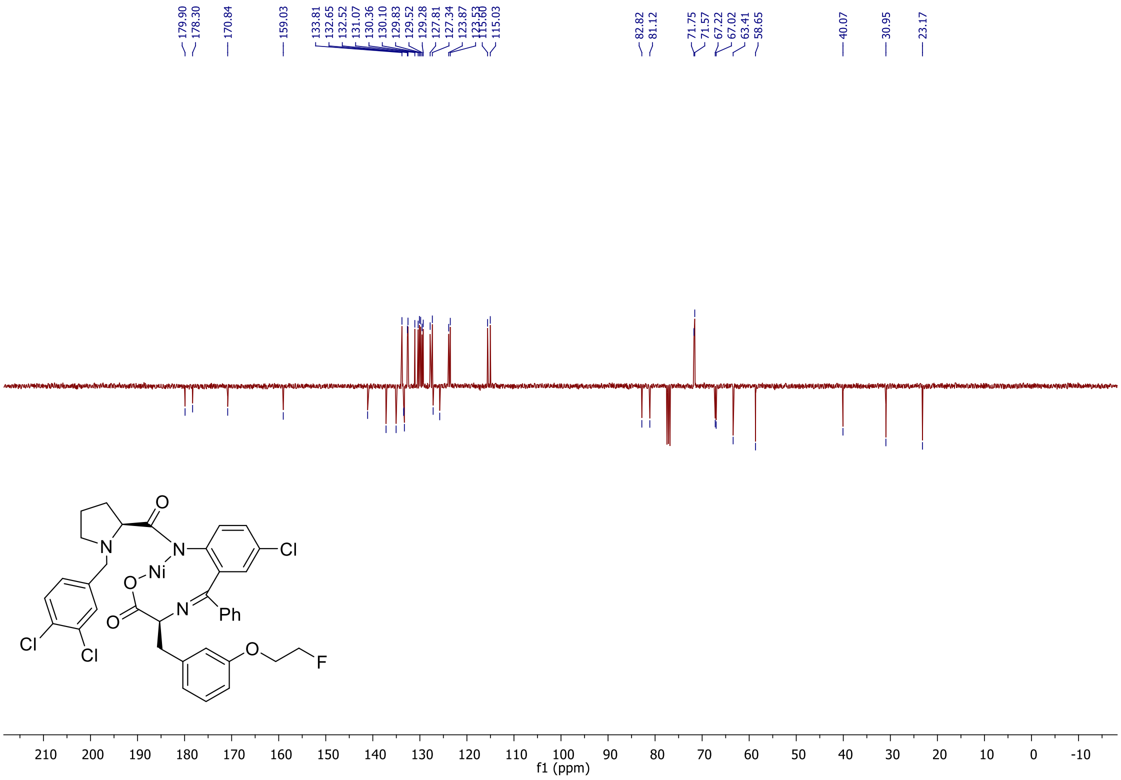

^{19}F-NMR spectrum of **4**

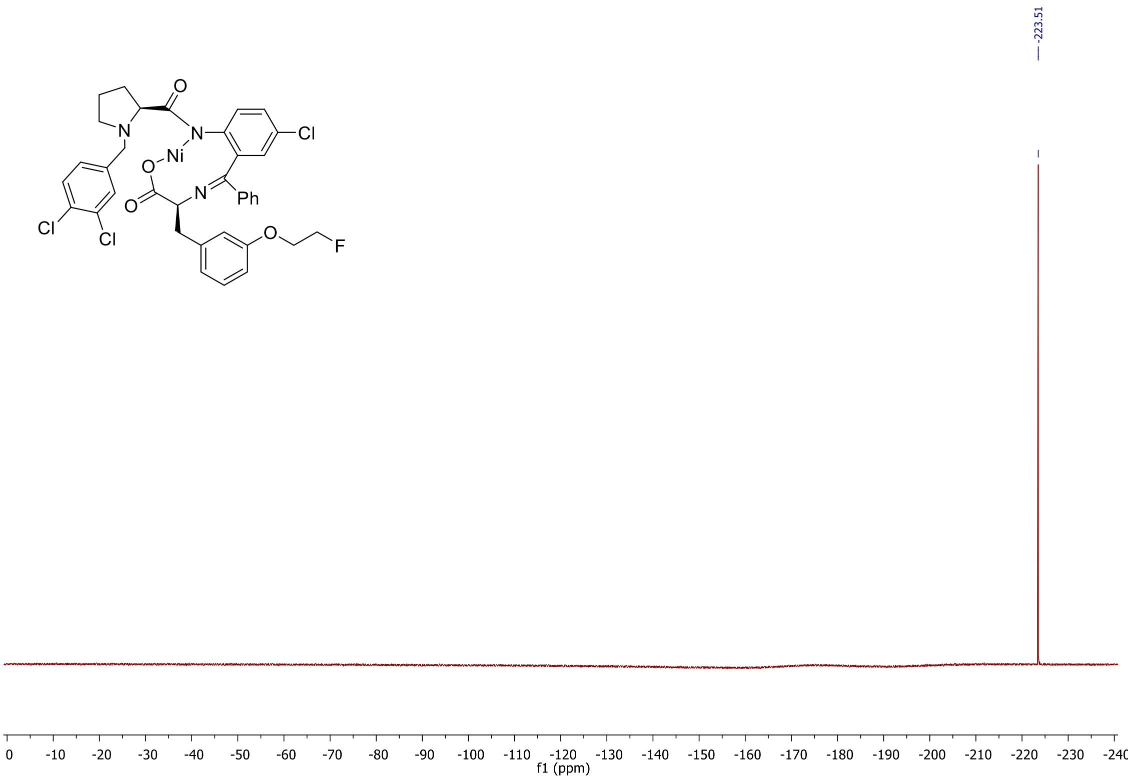

1.4 *m*-FET

^{1}H-NMR spectrum of *m*-FET

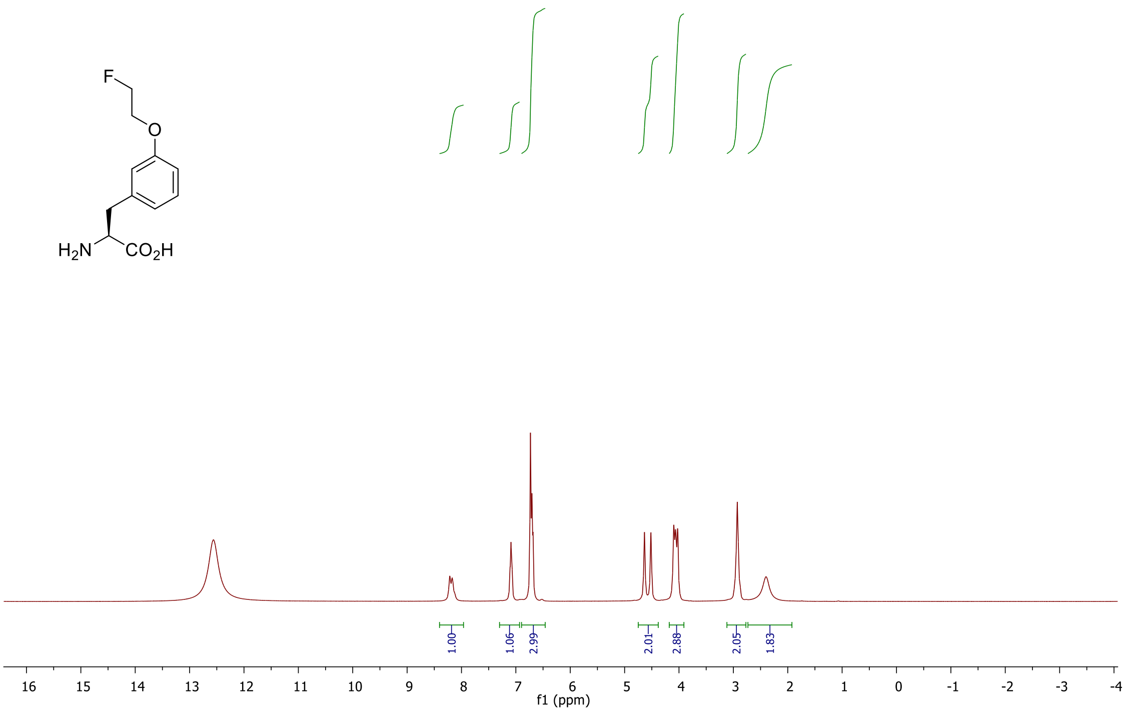

^{13}C-NMR spectrum of *m*-FET

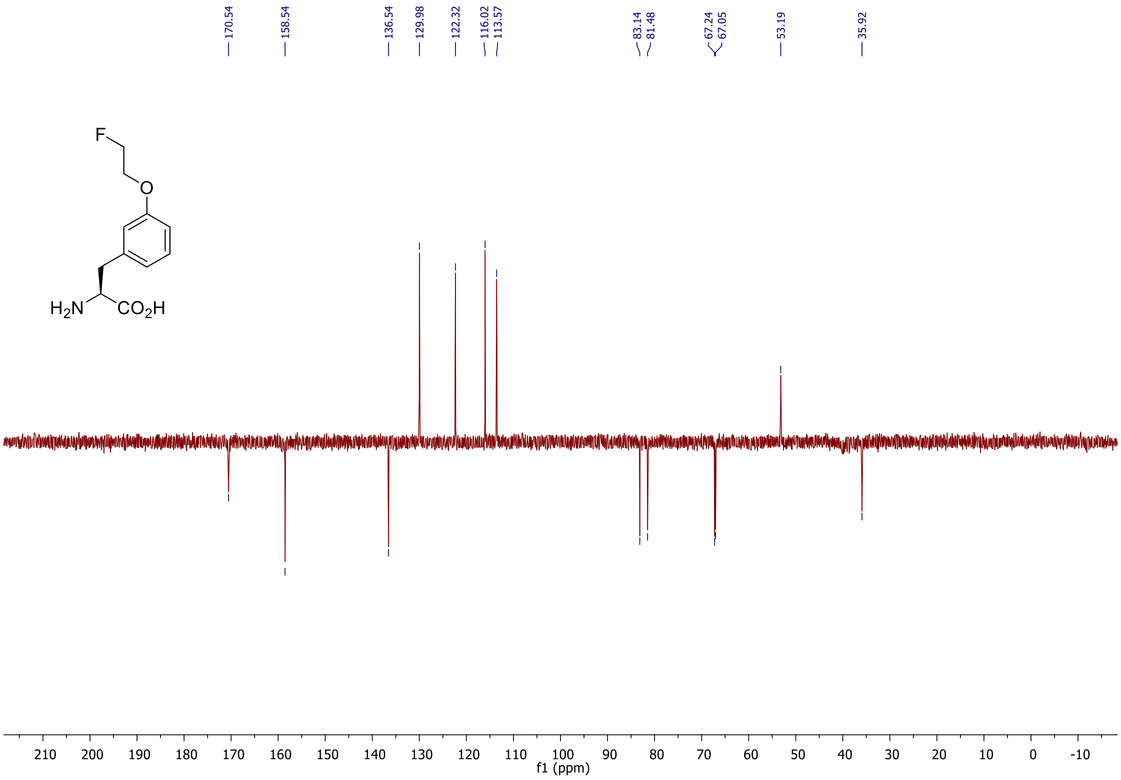

^{19}F-NMR spectrum of *m*-FET

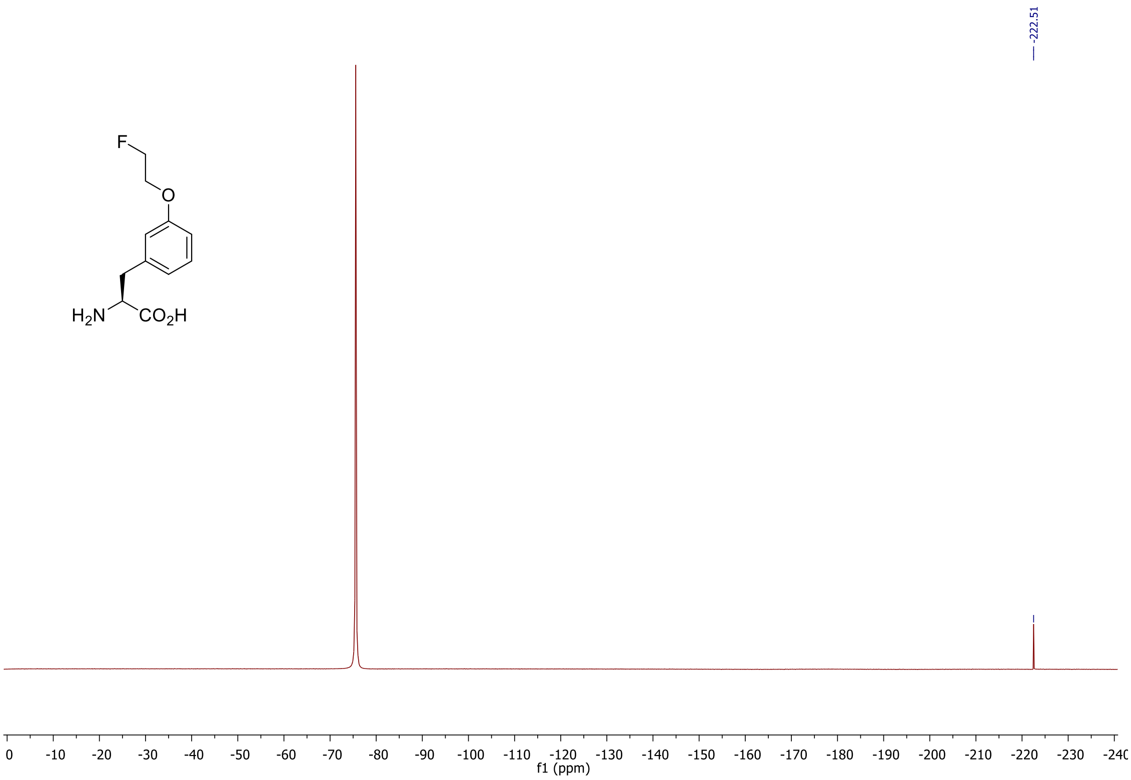

1.5 Boc-(*RS*)-*m*-FET-OMe

<u>^{1}H-NMR spectrum of Boc-(*RS*)-*m*-FET-OMe</u>

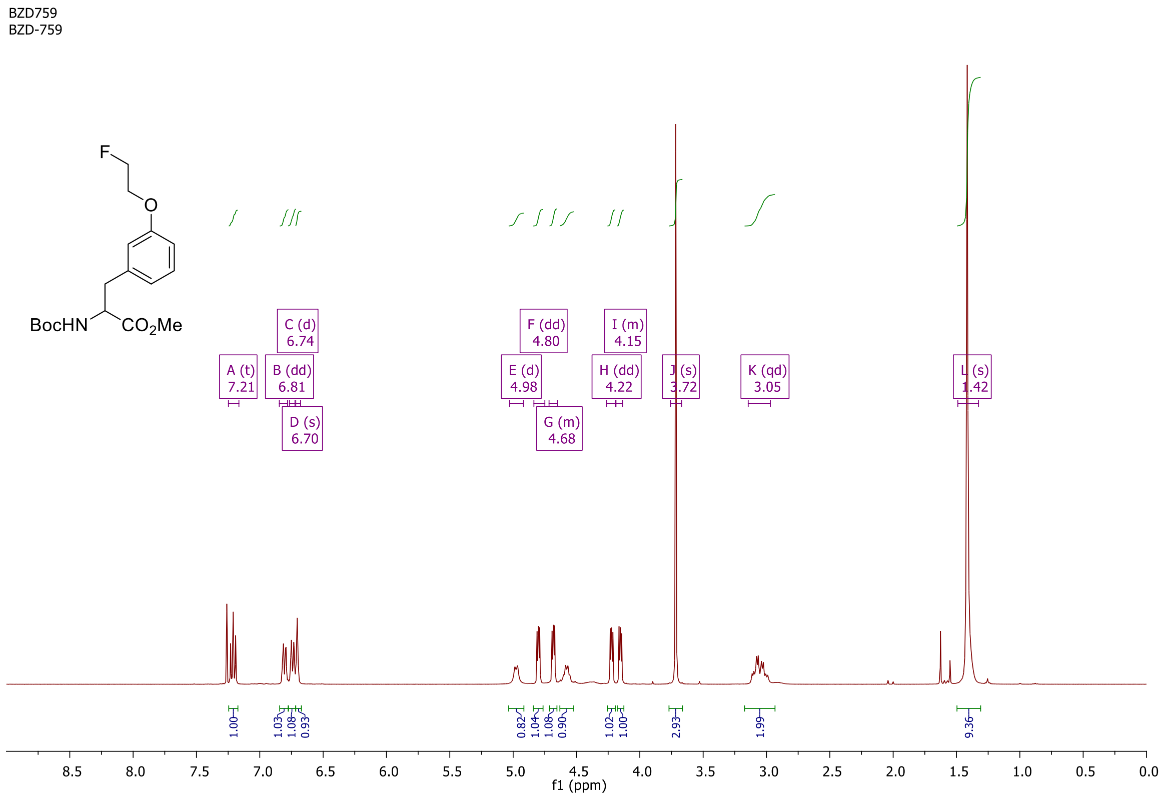

<u>^{13}C-NMR spectrum of Boc-(*RS*)-*m*-FET-OMe</u>

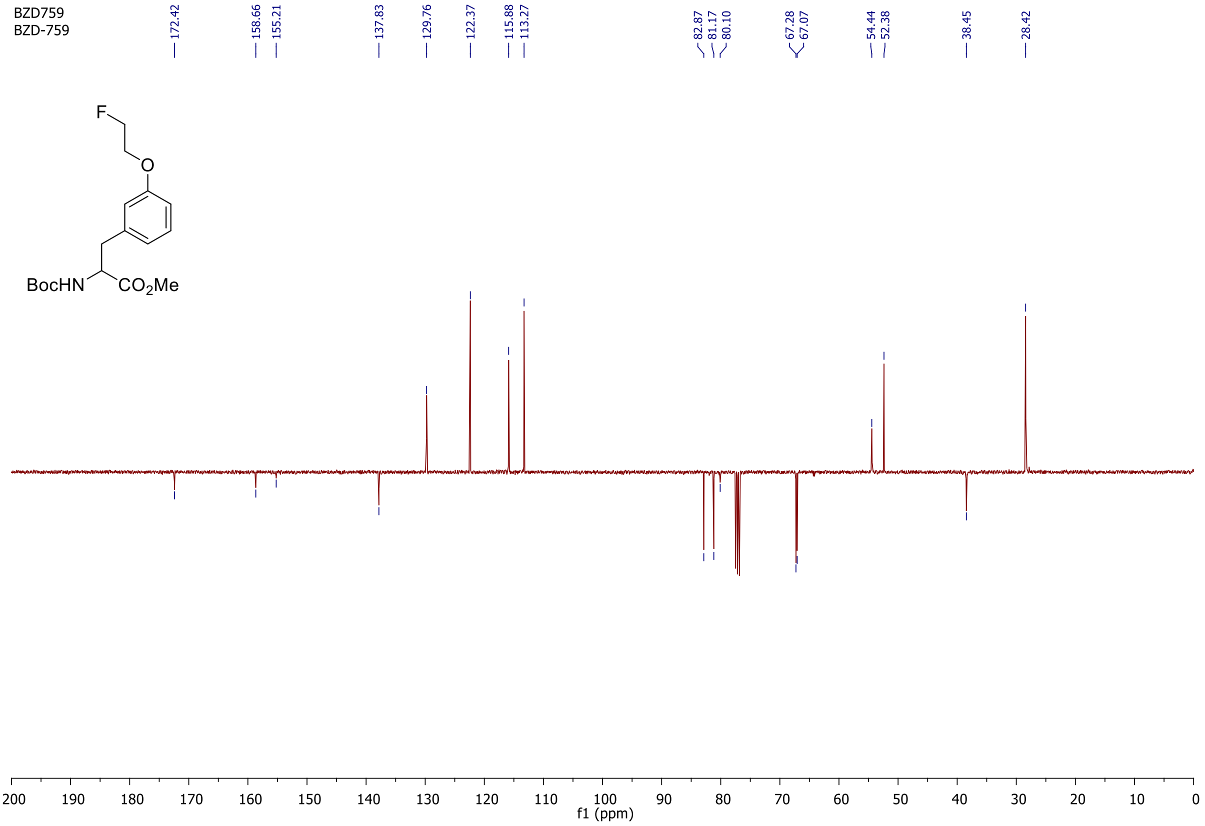

^{19}F-NMR spectrum of Boc-(*RS*)-*m*-FET-OMe

BZD759
BZD-759

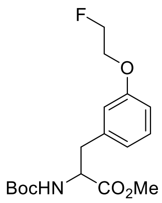

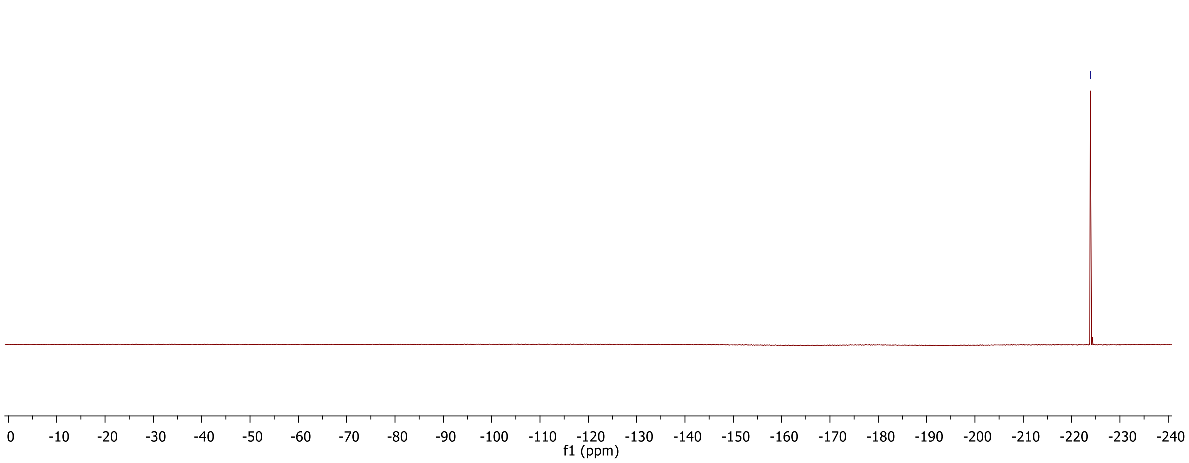

1.6 Boc-(*RS*)-*m*-FET-OH

<ins>^{1}H-NMR spectrum of Boc-(*RS*)-*m*-FET-OH</ins>

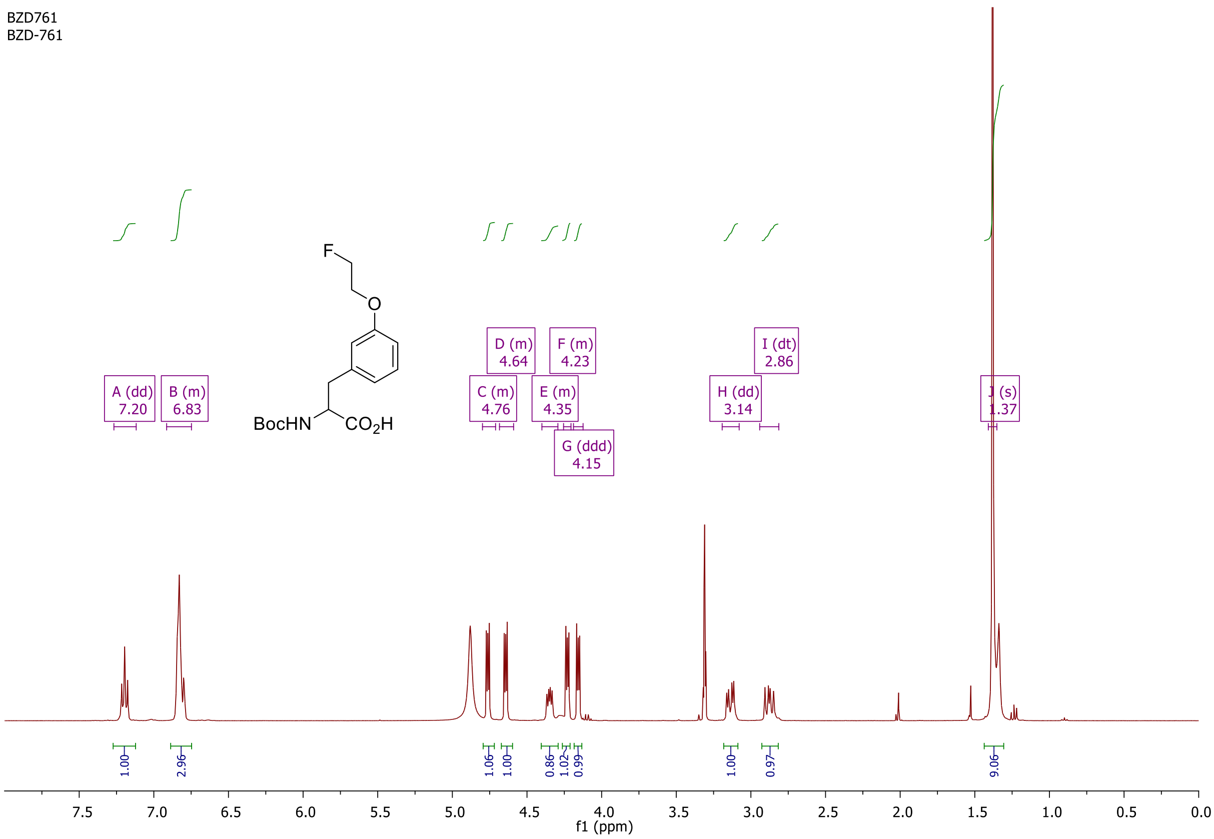

<ins>^{13}C-NMR spectrum of Boc-(*RS*)-*m*-FET-OH</ins>

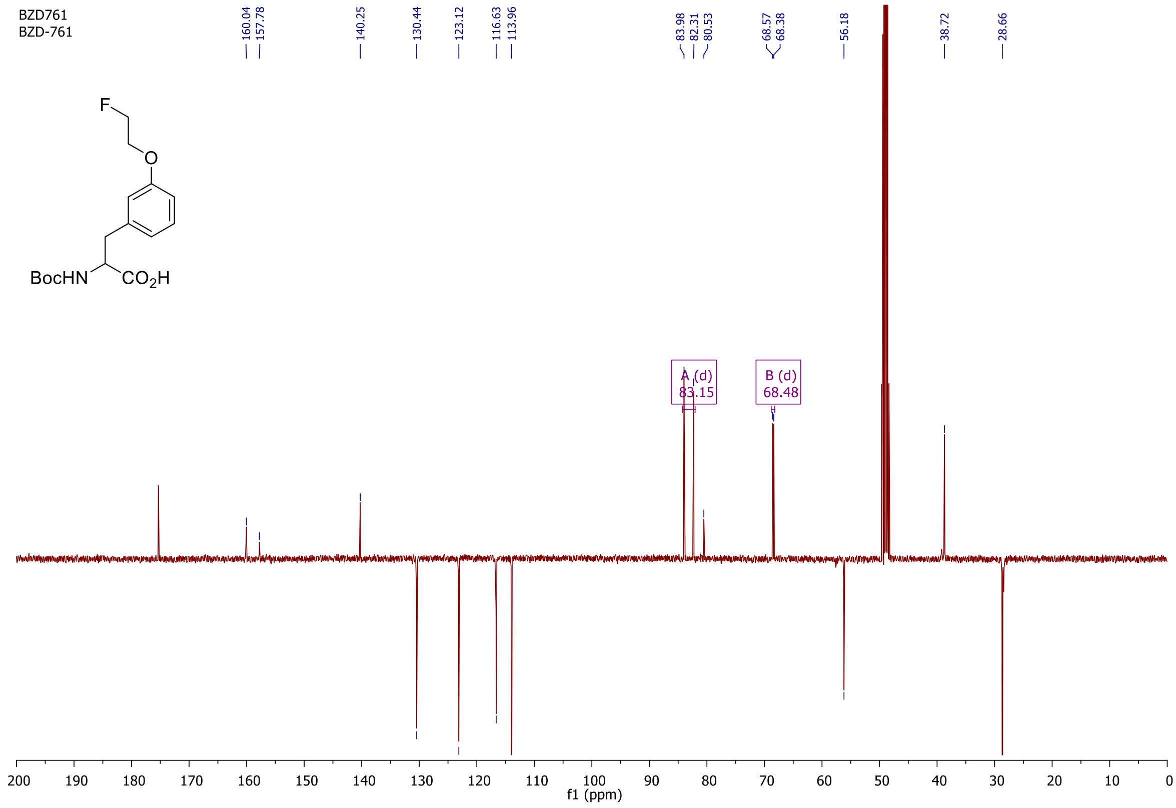

^{19}F-NMR spectrum of Boc-(*RS*)-*m*-FET-OH

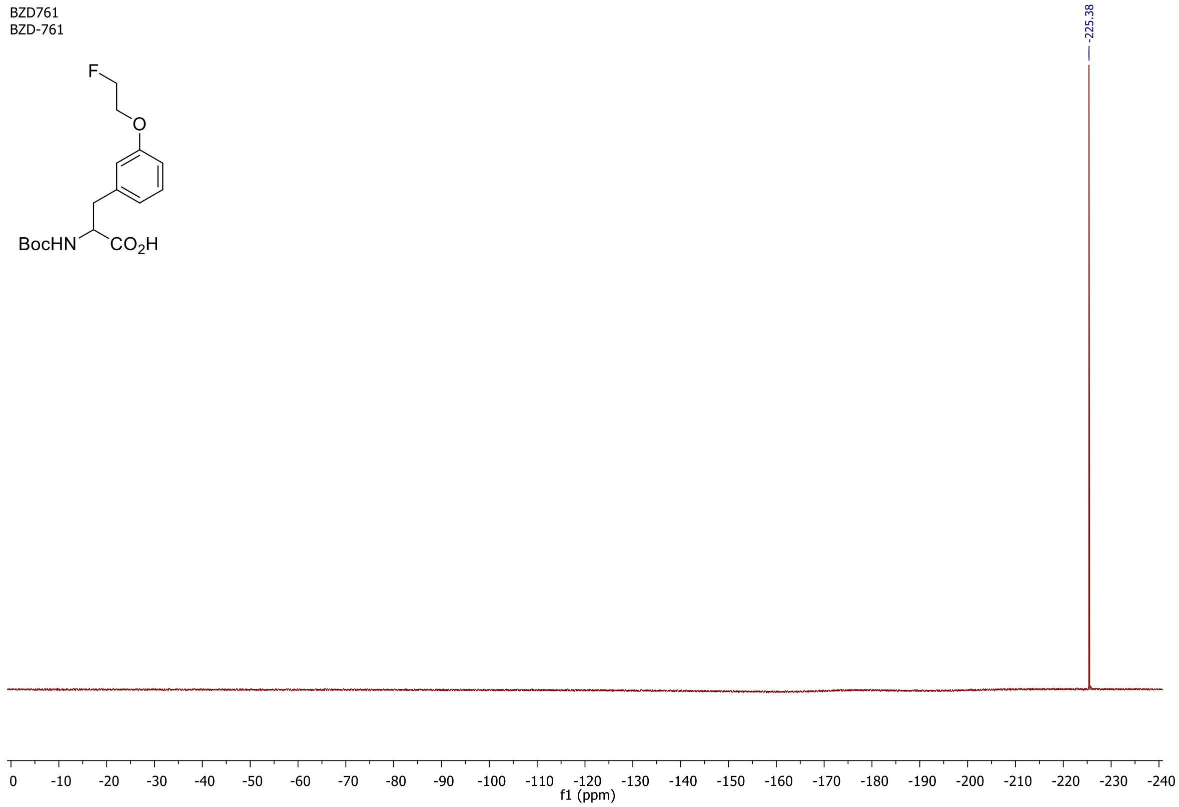

1.7 HCl·(*RS*)-*m*-FET

^{1}H-NMR spectrum of HCl·(*RS*)-*m*-FET

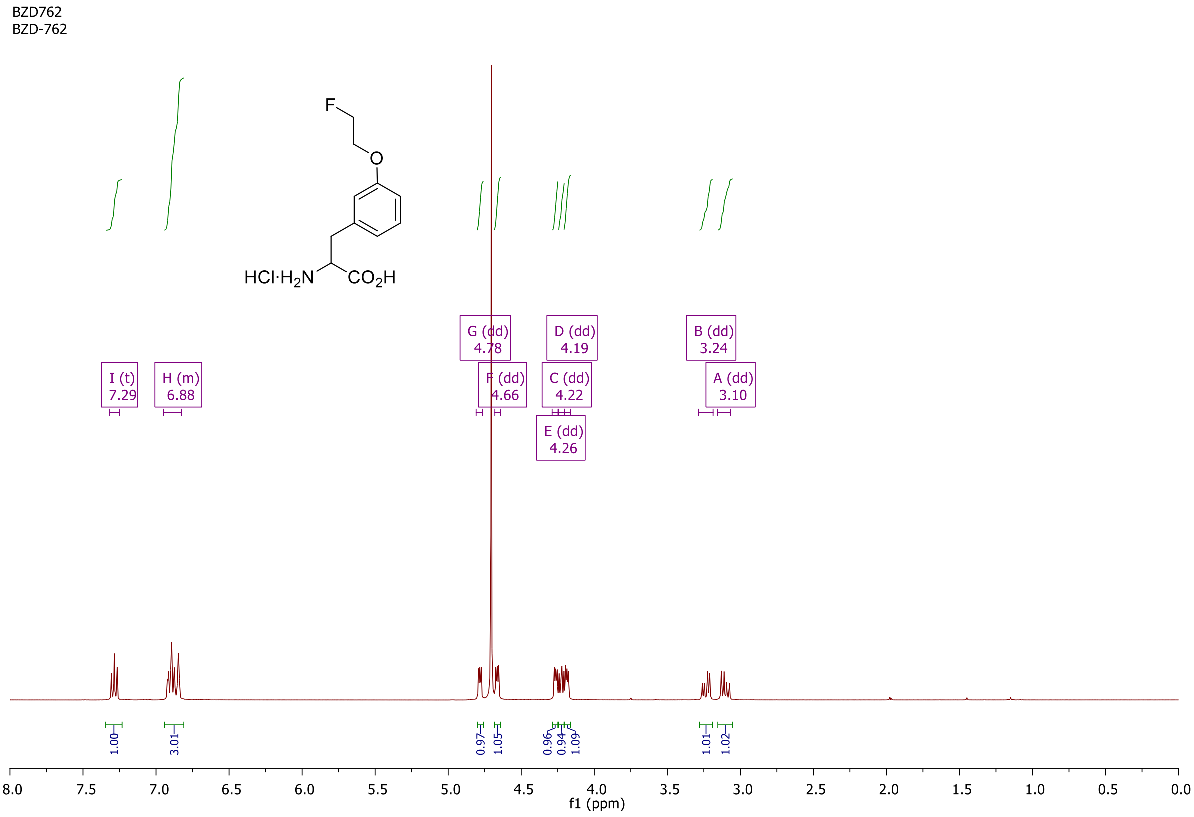

^{13}C-NMR spectrum of HCl·(*RS*)-*m*-FET

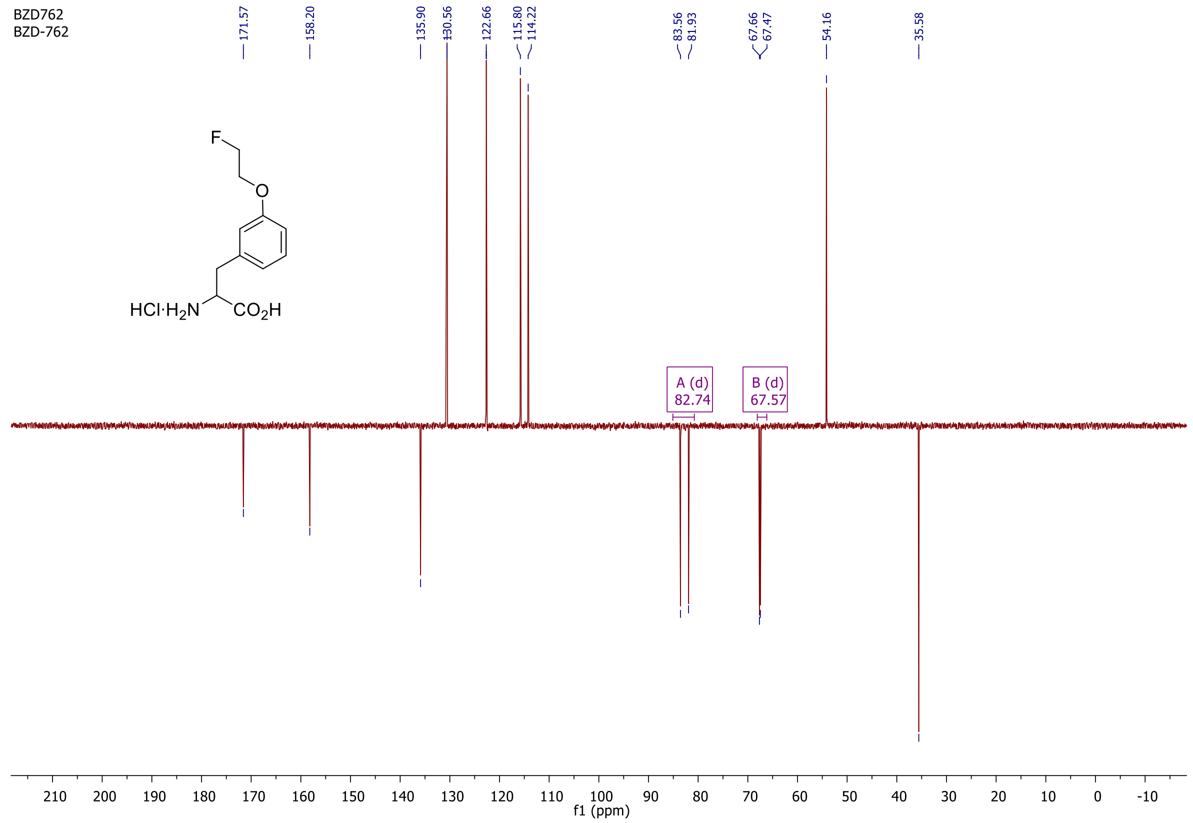

<u>^{19}F-NMR spectrum of HCl·(*RS*)-*m*-FET</u>

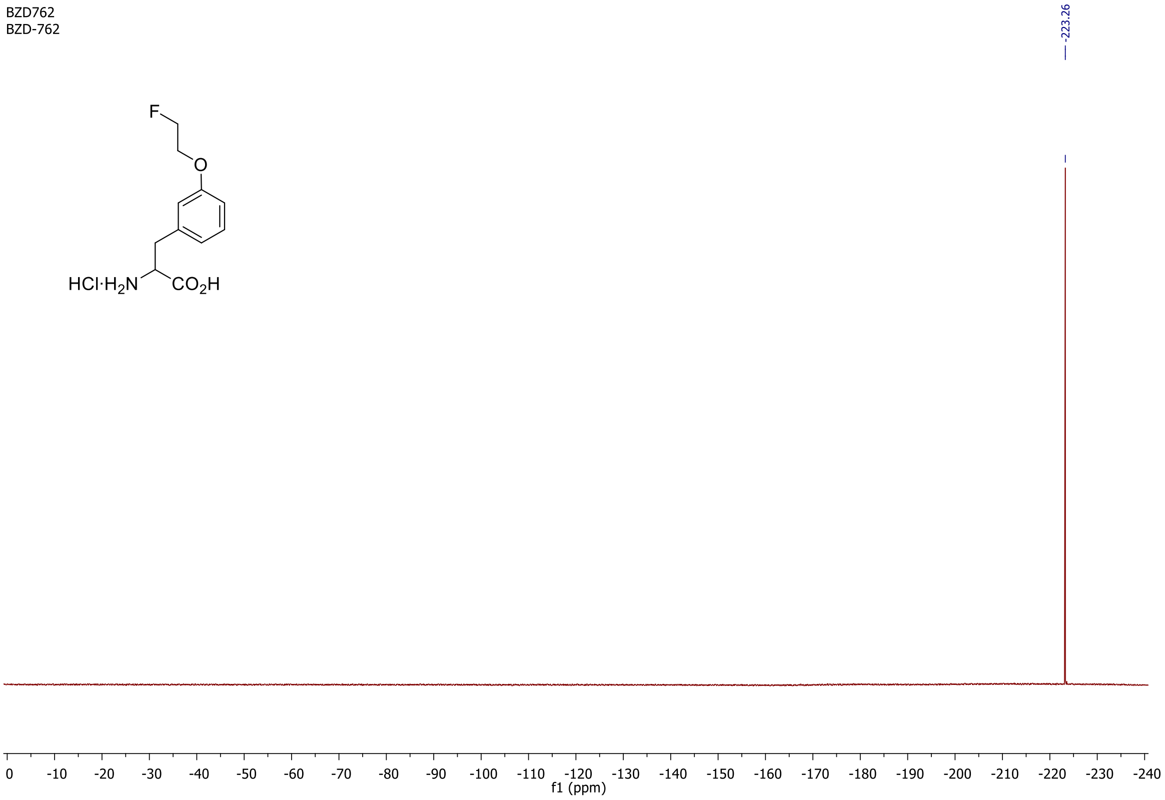

1.8 Compound **5**

^{1}H-NMR spectrum of **5**

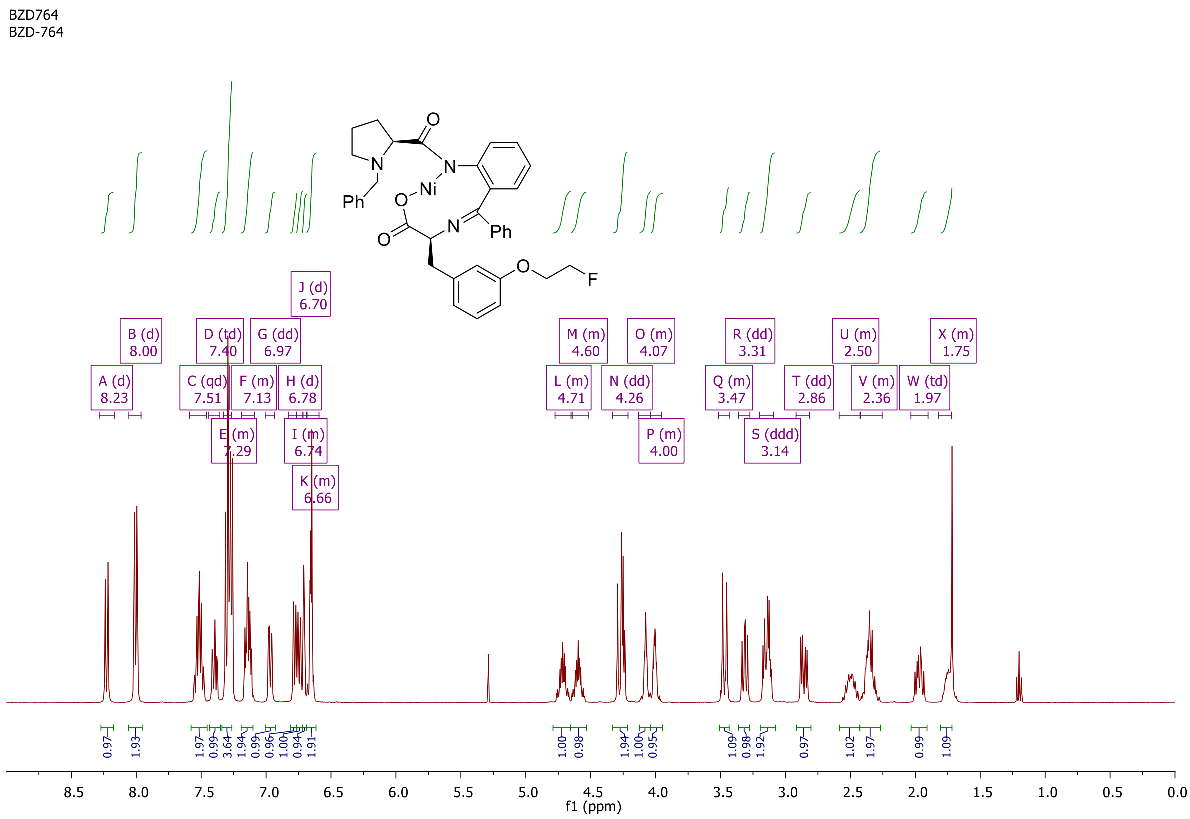

^{13}C-NMR spectrum of **5**

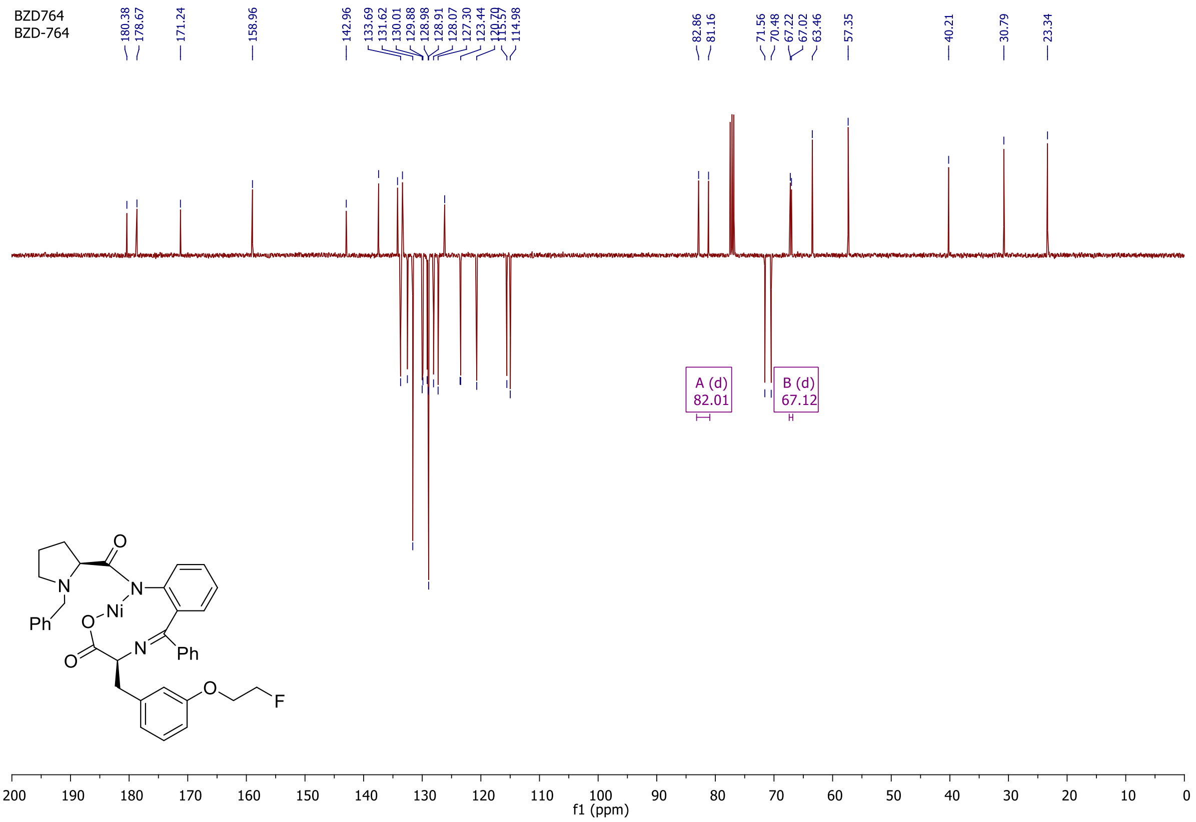

^{19}F-NMR spectrum of **5**

BZD764
BZD-764

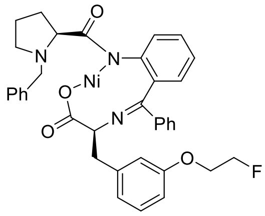

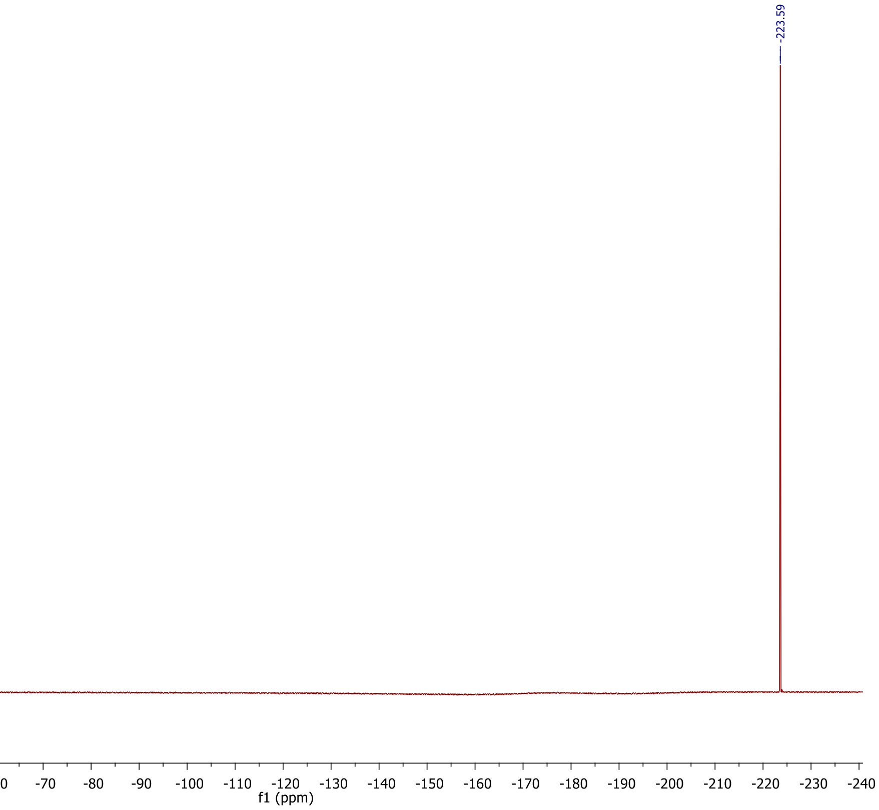

1.9 Compound **6**

<u>^{1}H-NMR spectrum of **6**</u>

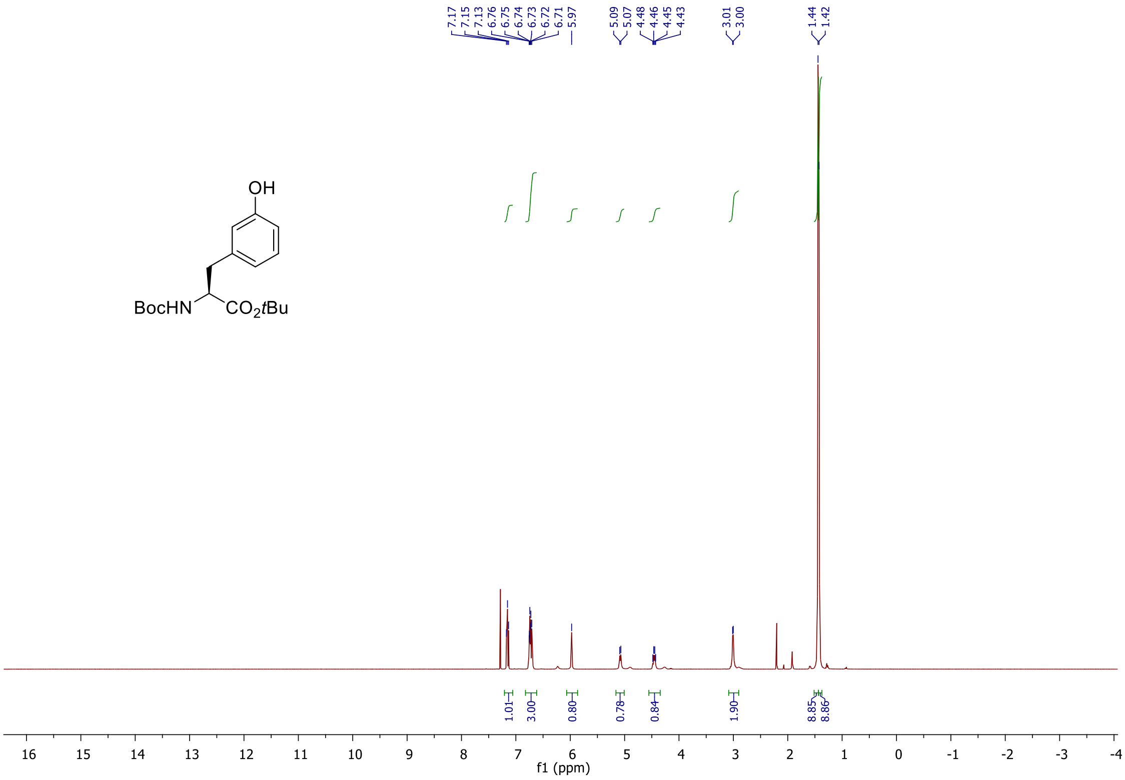

<u>^{13}C-NMR spectrum of **6**</u>

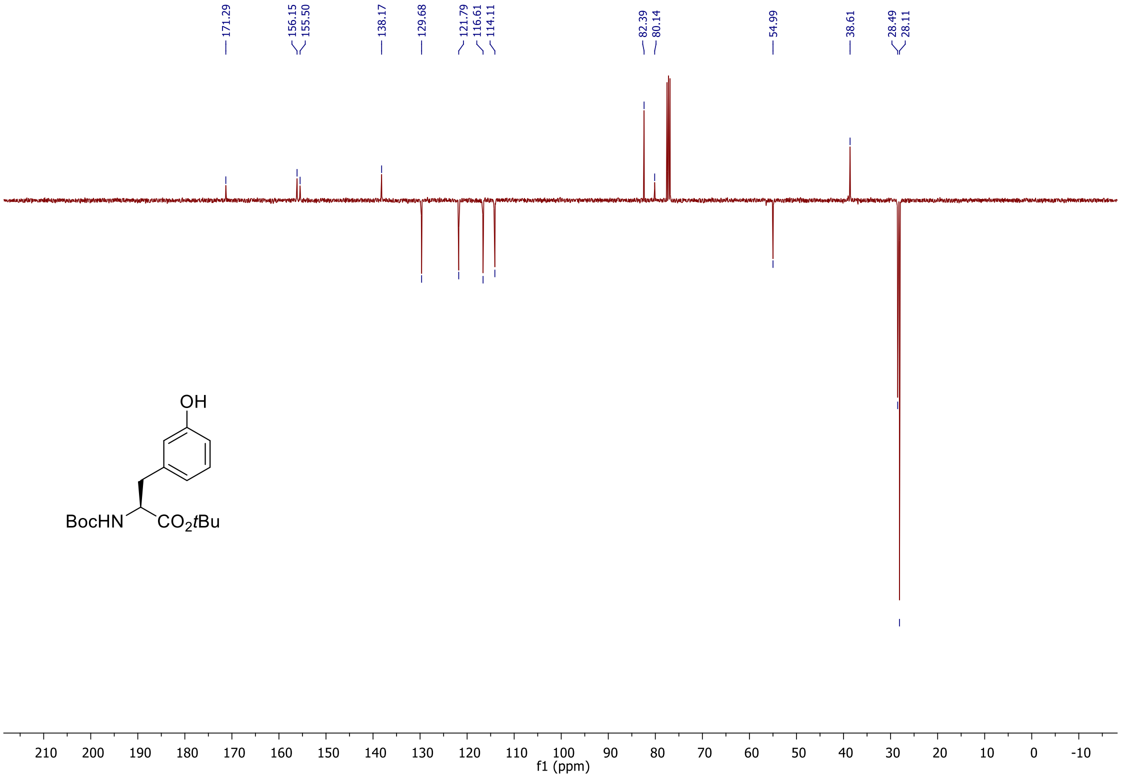

1.10 Compound **7**

^{1}H-NMR spectrum of **7**

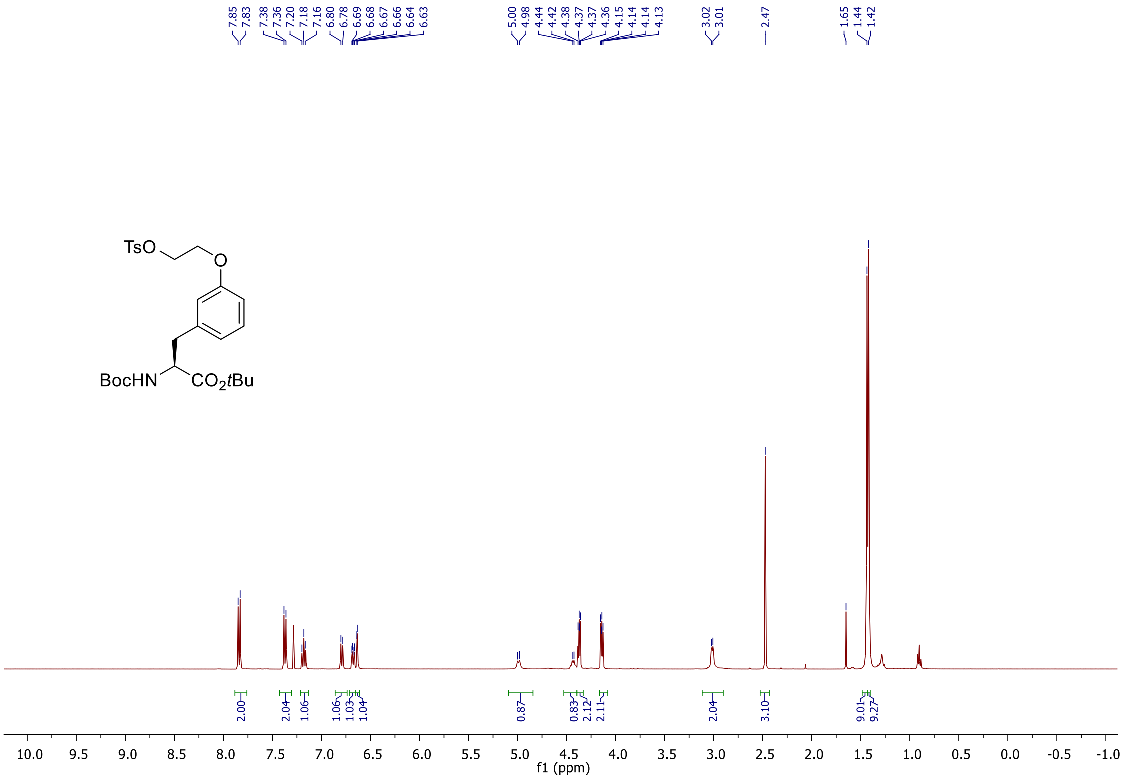

^{13}C-NMR spectrum of **7**

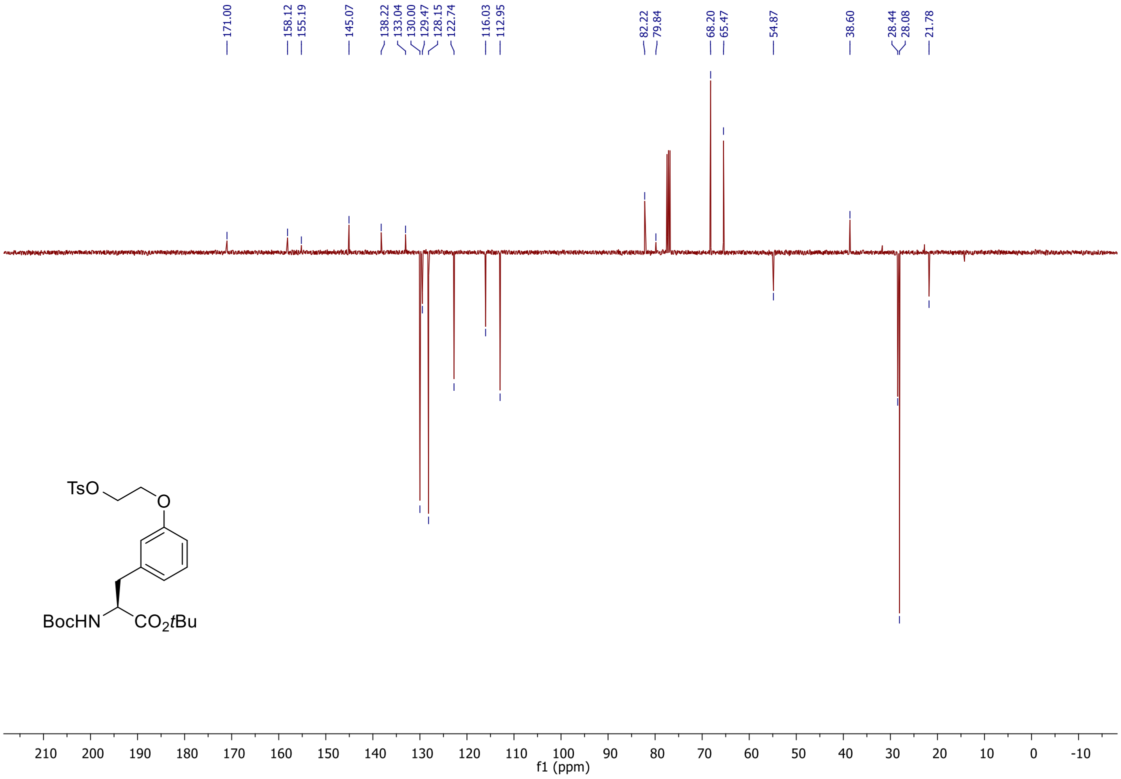

1.11 Compound (*RS*)-**8**

^{1}H-NMR spectrum of (*RS*)-**8**

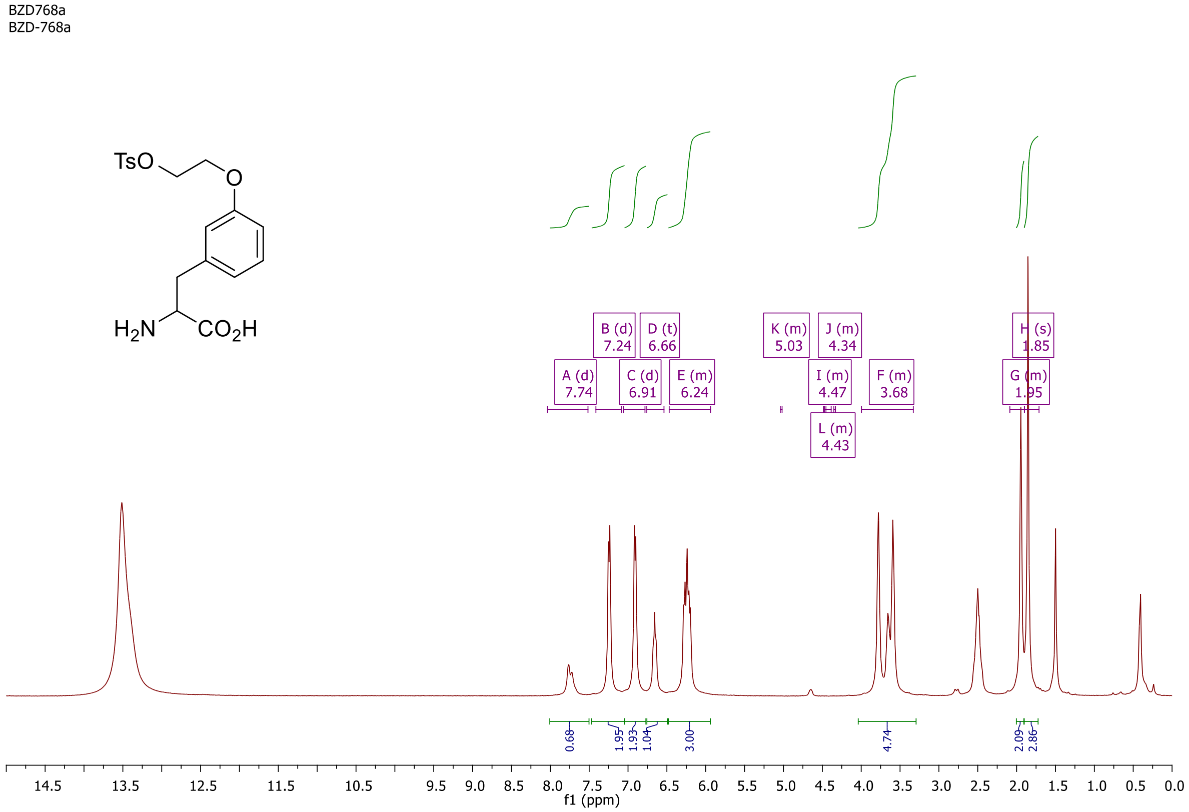

^{13}C-NMR spectrum of (*RS*)-**8**

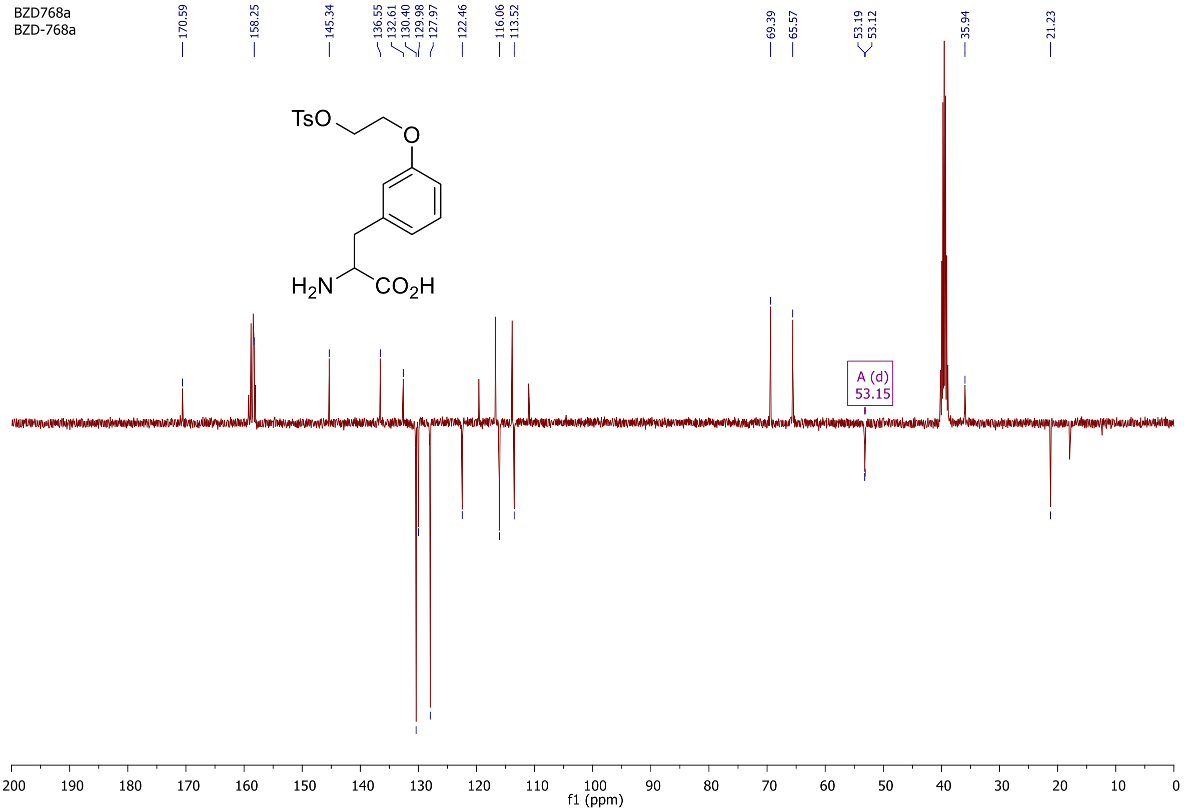

1.12 Compound **9**

^{1}H-NMR spectrum of **9**

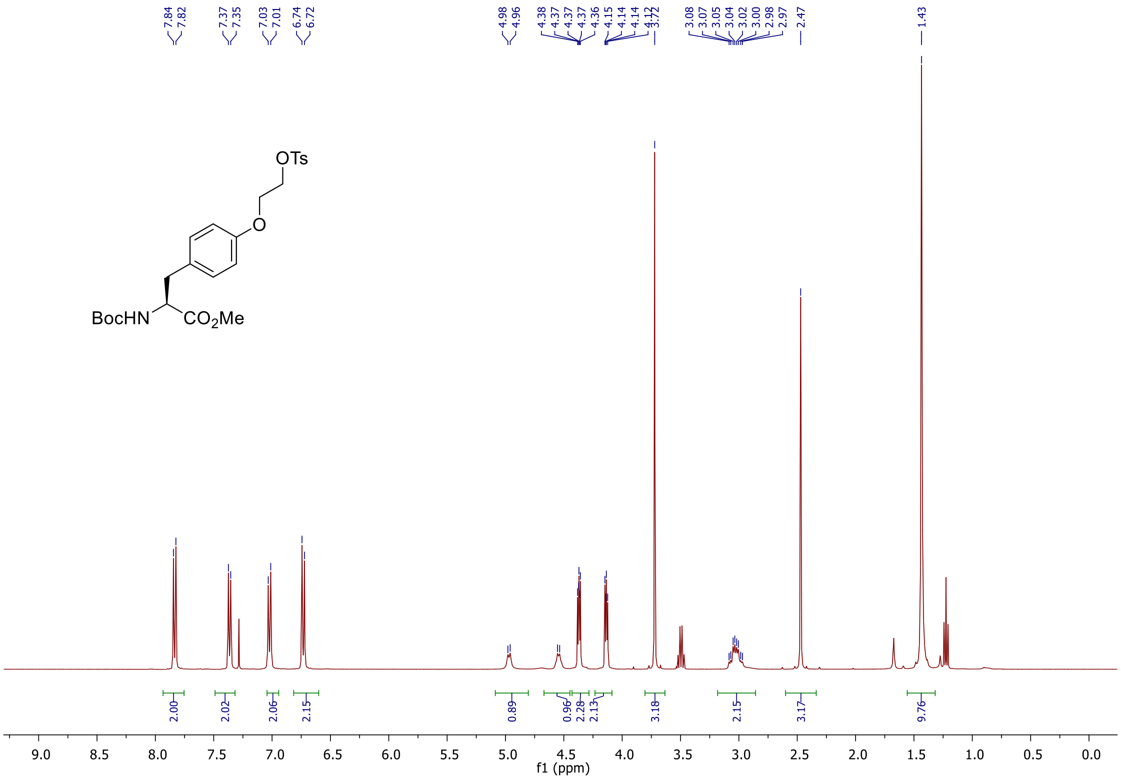

^{13}C-NMR spectrum of **9**

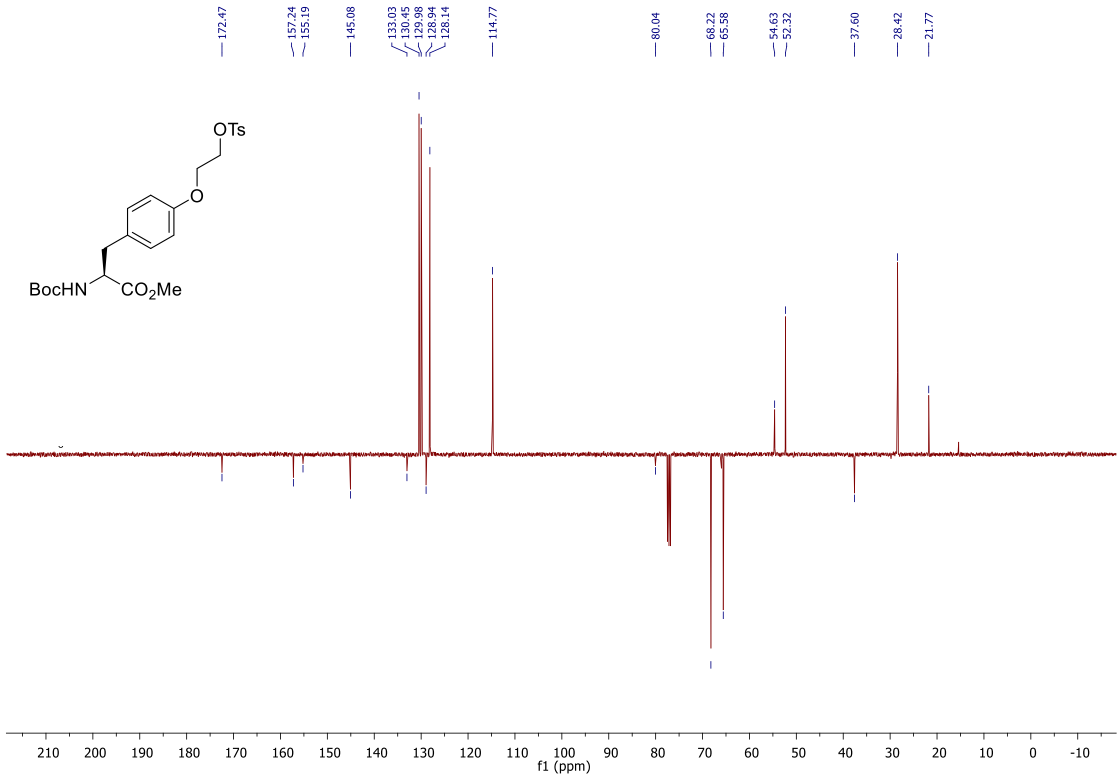

1.13 HCl·FET-OMe

^{1}H-NMR spectrum of HCl·FET-OMe

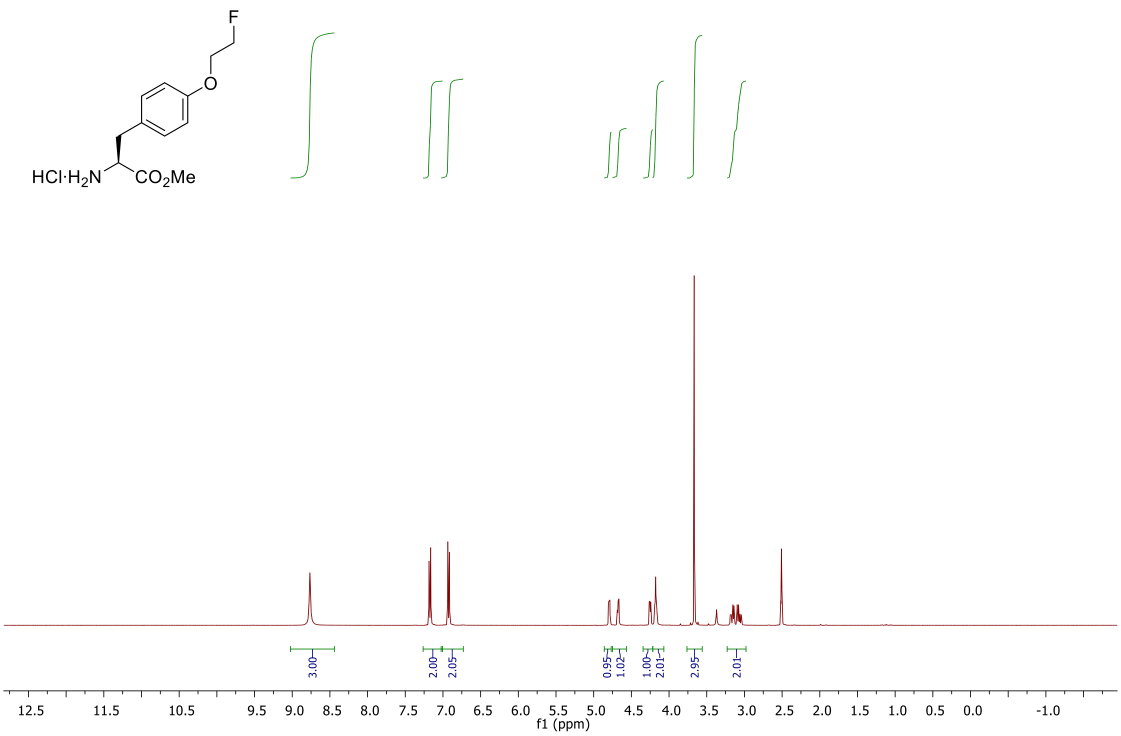

^{13}C-NMR spectrum of HCl·FET-OMe

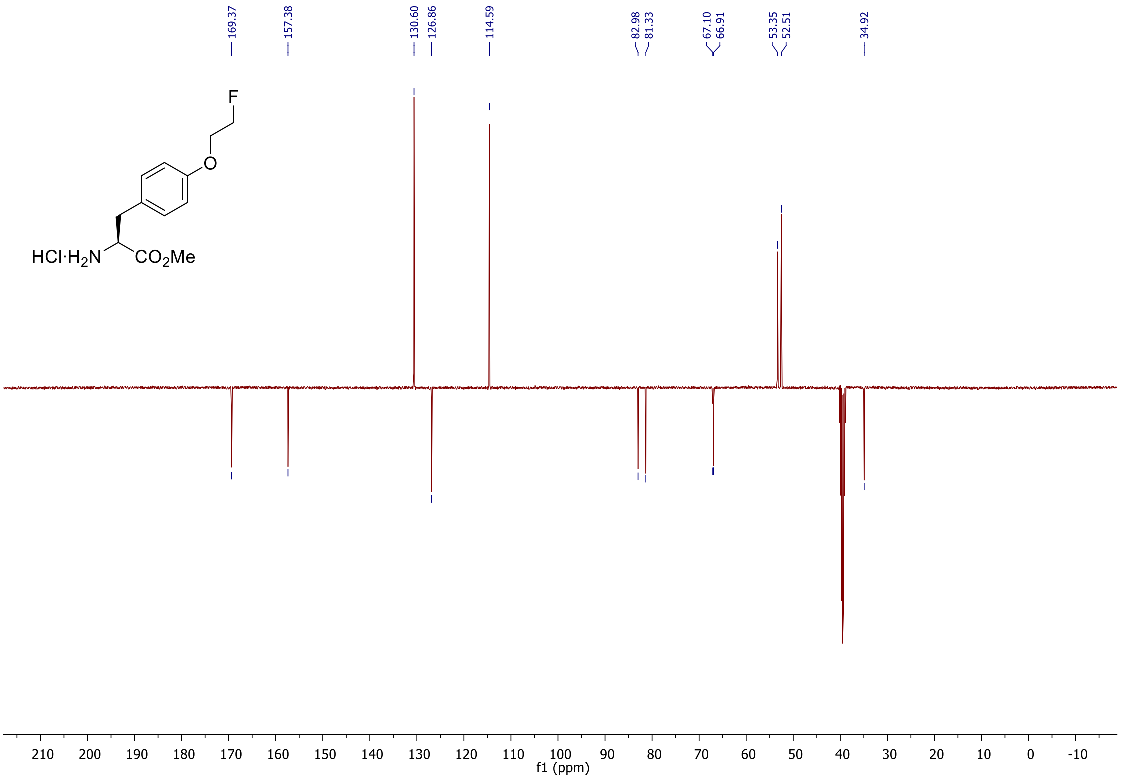

^{19}F-NMR spectrum of HCl·FET-OMe

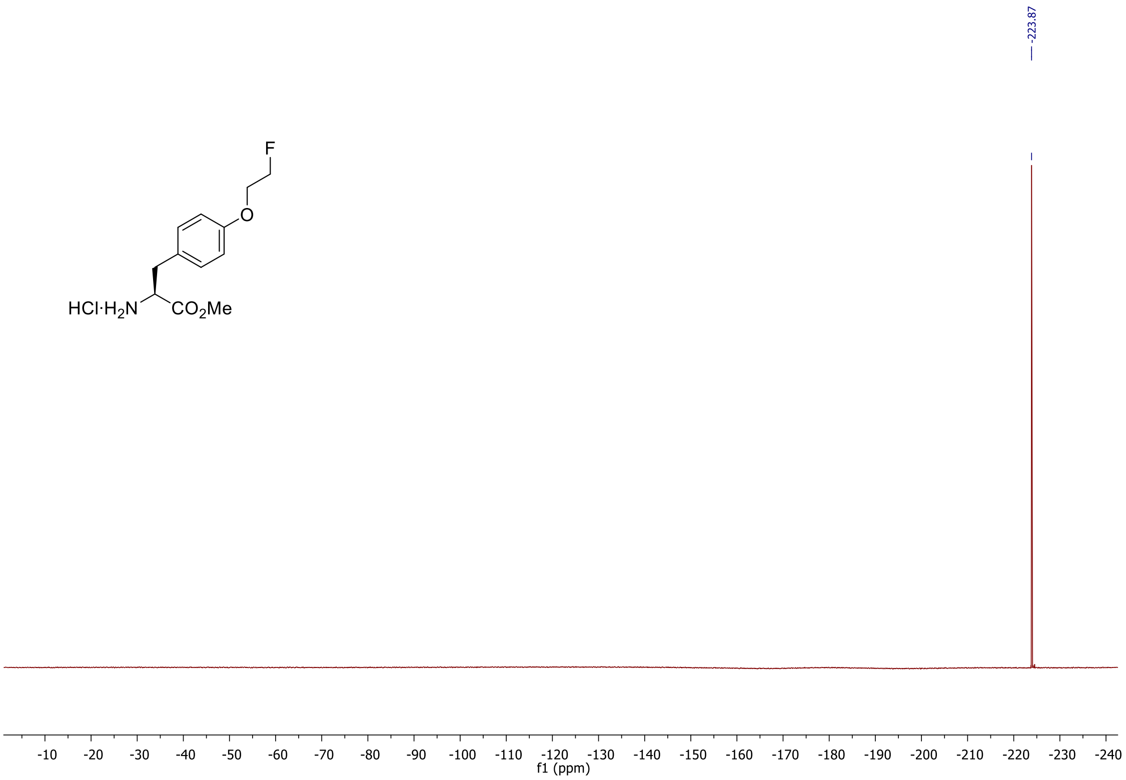

1.14 (*RS*)-FET

^{1}H-NMR spectrum of (*RS*)-FET

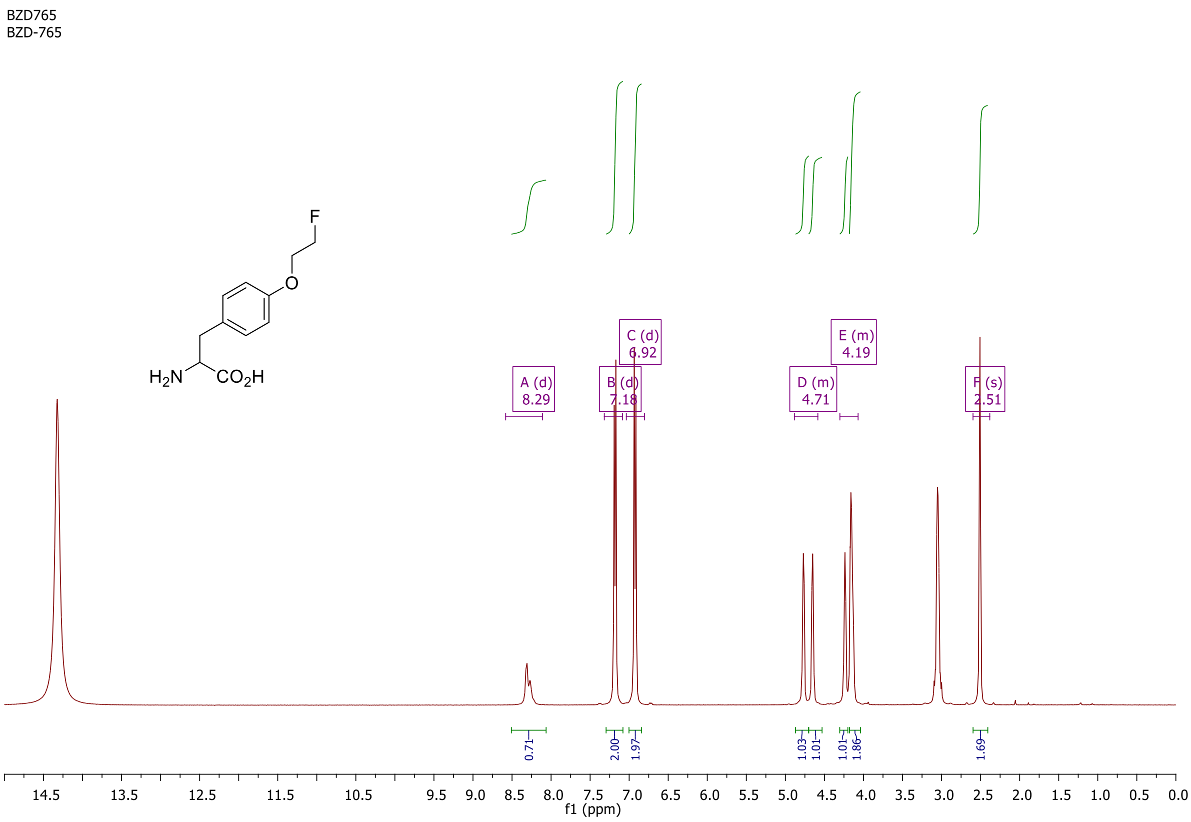

^{13}C-NMR spectrum of (*RS*)-FET

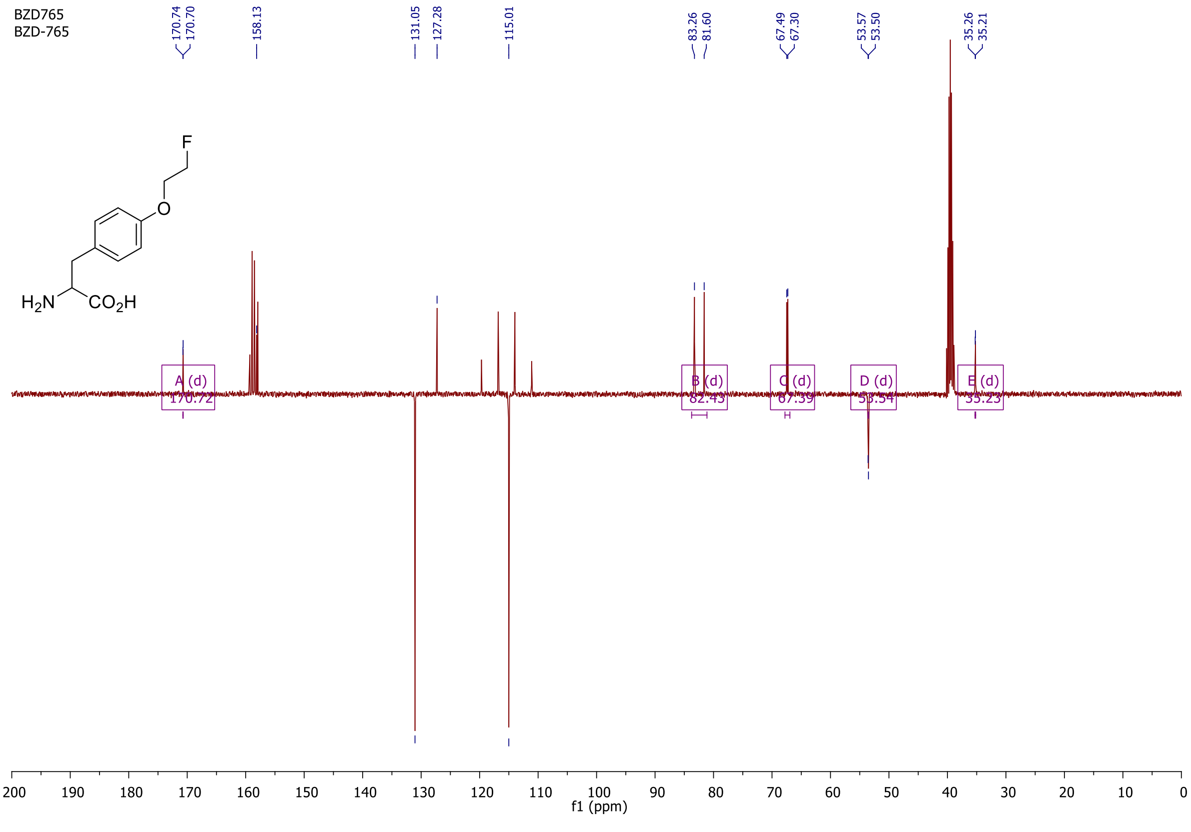

^{19}F-NMR spectrum of (*RS*)-FET

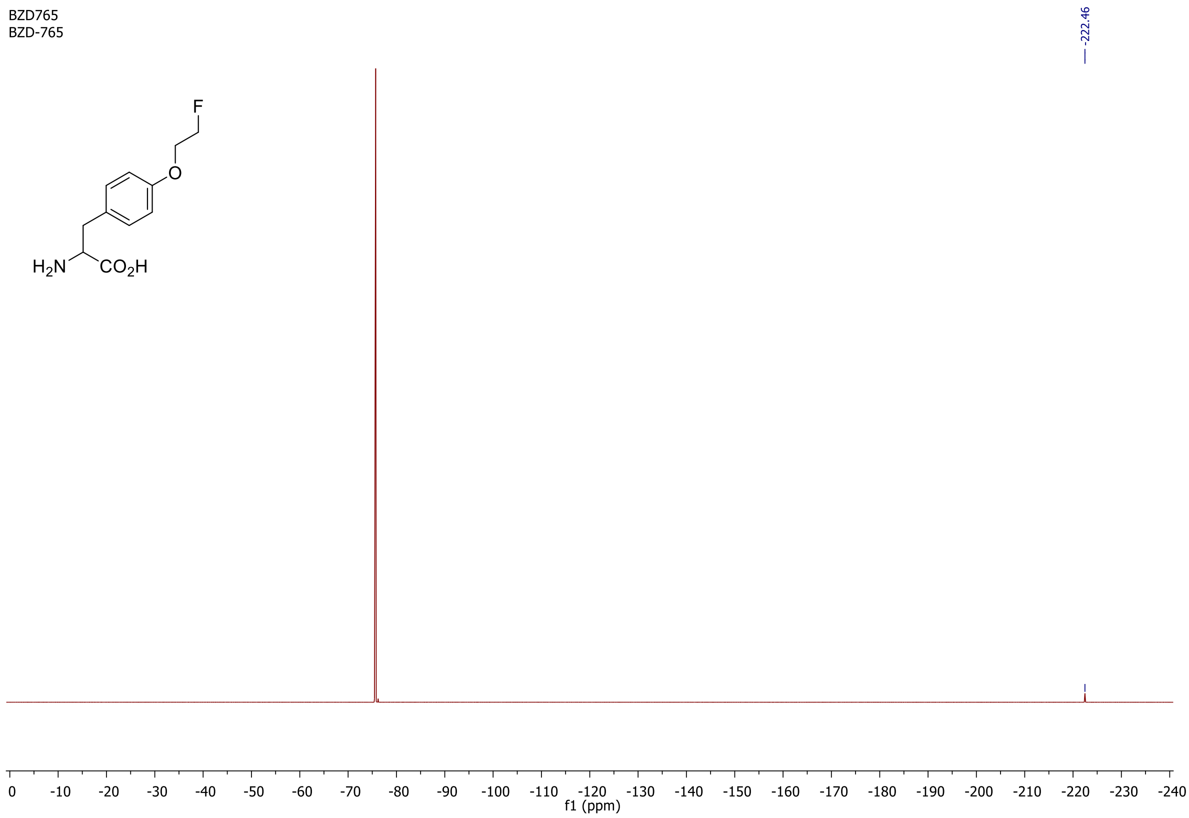

2 HPLC chromatograms

2.1 Quality control HPLC chromatograms of the reference compounds

HPLC chromatogram of (*S*)-*m*-FET

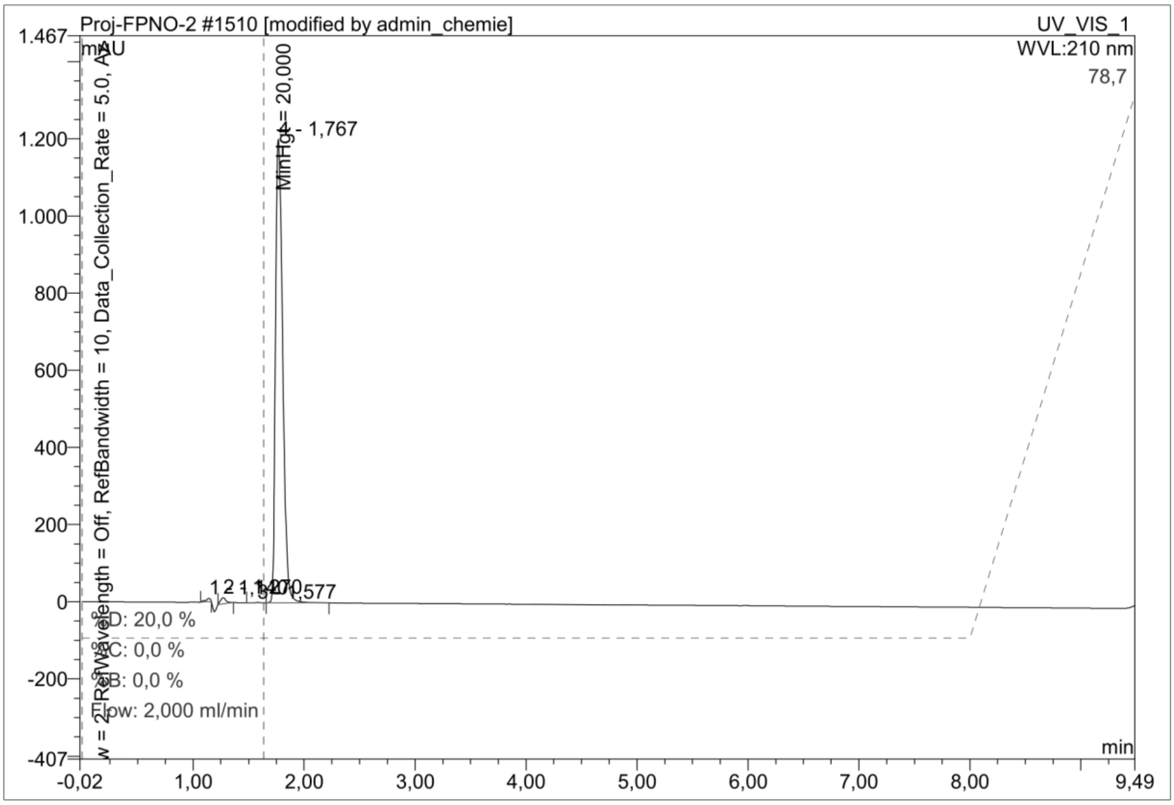

No.	Ret.Time min	Peak Name	Height mAU	Area mAU*min	Rel.Area %	Amount	Type
1	1,14	n.a.	8,666	0,419	0,45	n.a.	BMB*
2	1,27	n.a.	15,691	0,884	0,96	n.a.	BMB*
3	1,58	n.a.	0,496	0,023	0,03	n.a.	BMb*
4	1,77	n.a.	1200,663	90,903	98,56	n.a.	bMB
**Total:**			1225,515	92,229	100,00	0,000	

HPLC trace of (*S*)-*m*-FET (UV, λ = 210 nm). Column: Chromolith HighResolution RP-18e, 150×4.6 mm; eluent: 20% MeCN (0.1% TFA); flow rate: 2.0 mL/min.

HPLC chromatogram of (*RS*)-*m*-FET

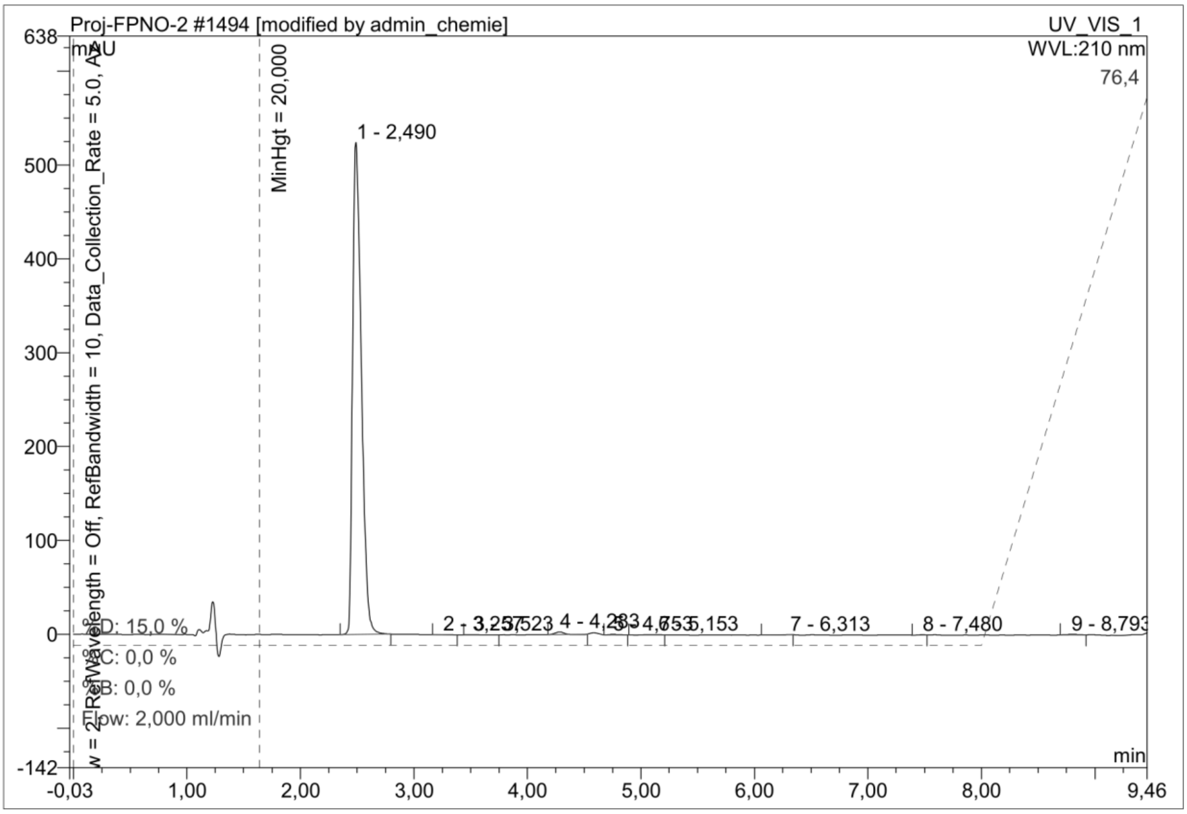

No.	Ret.Time min	Peak Name	Height mAU	Area mAU*min	Rel.Area %	Amount	Type
1	2,49	n.a.	524,101	46,851	99,13	n.a.	BMB*
2	3,26	n.a.	0,315	0,037	0,08	n.a.	BMB*
3	3,52	n.a.	0,338	0,041	0,09	n.a.	BMB*
4	4,28	n.a.	2,667	0,180	0,38	n.a.	BMB*
5	4,75	n.a.	0,523	0,044	0,09	n.a.	BMB*
6	5,15	n.a.	0,278	0,020	0,04	n.a.	BMB*
7	6,31	n.a.	0,118	0,010	0,02	n.a.	BMB*
8	7,48	n.a.	0,294	0,015	0,03	n.a.	BMB*
9	8,79	n.a.	0,473	0,064	0,13	n.a.	BMB*
**Total:**			529,106	47,261	100,00	0,000	

HPLC trace of (*RS*)-*m*-FET (UV, λ = 210 nm). Column: Chromolith HighResolution RP-18e, 150×4.6 mm; eluent: 15% MeCN (0.1% TFA); flow rate: 2.0 mL/min.

<u>HPLC chromatogram of (*S*)-FET-OMe</u>

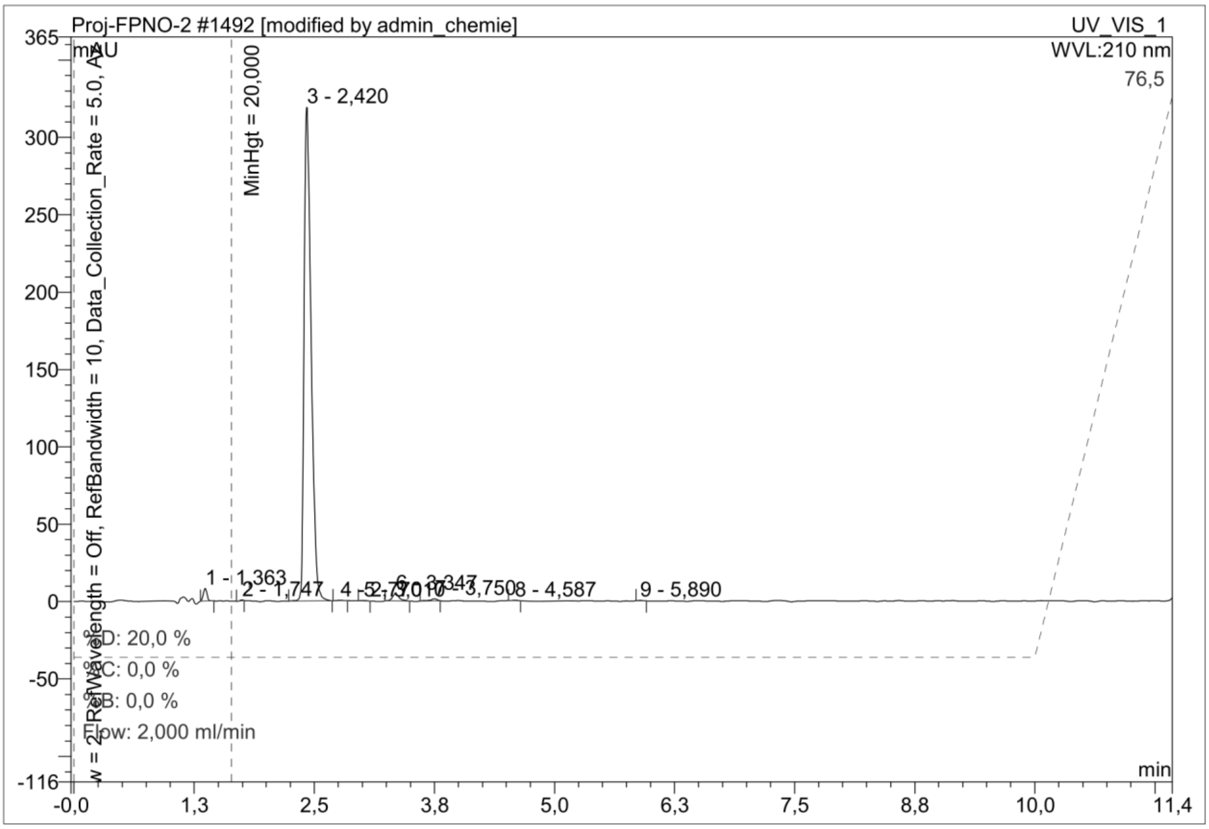

No.	Ret.Time min	Peak Name	Height mAU	Area mAU*min	Rel.Area %	Amount	Type
1	1,36	n.a.	8,182	0,359	1,28	n.a.	BMB*
2	1,75	n.a.	0,525	0,017	0,06	n.a.	BMB*
3	2,42	n.a.	319,043	27,027	96,50	n.a.	BMB
4	2,77	n.a.	0,249	0,021	0,07	n.a.	BMB*
5	3,01	n.a.	0,335	0,023	0,08	n.a.	BMB*
6	3,35	n.a.	4,634	0,399	1,43	n.a.	BMB*
7	3,75	n.a.	1,294	0,118	0,42	n.a.	BMB*
8	4,59	n.a.	0,313	0,022	0,08	n.a.	BMB*
9	5,89	n.a.	0,324	0,021	0,08	n.a.	BMB*
**Total:**			334,900	28,008	100,00	0,000	

HPLC trace of (*S*)-FET-OMe (UV, λ = 210 nm). Column: Chromolith HighResolution RP-18e, 150×4.6 mm; eluent: 20% MeCN (0.1% TFA); flow rate: 2.0 mL/min.

HPLC chromatogram of (*RS*)-FET-OMe

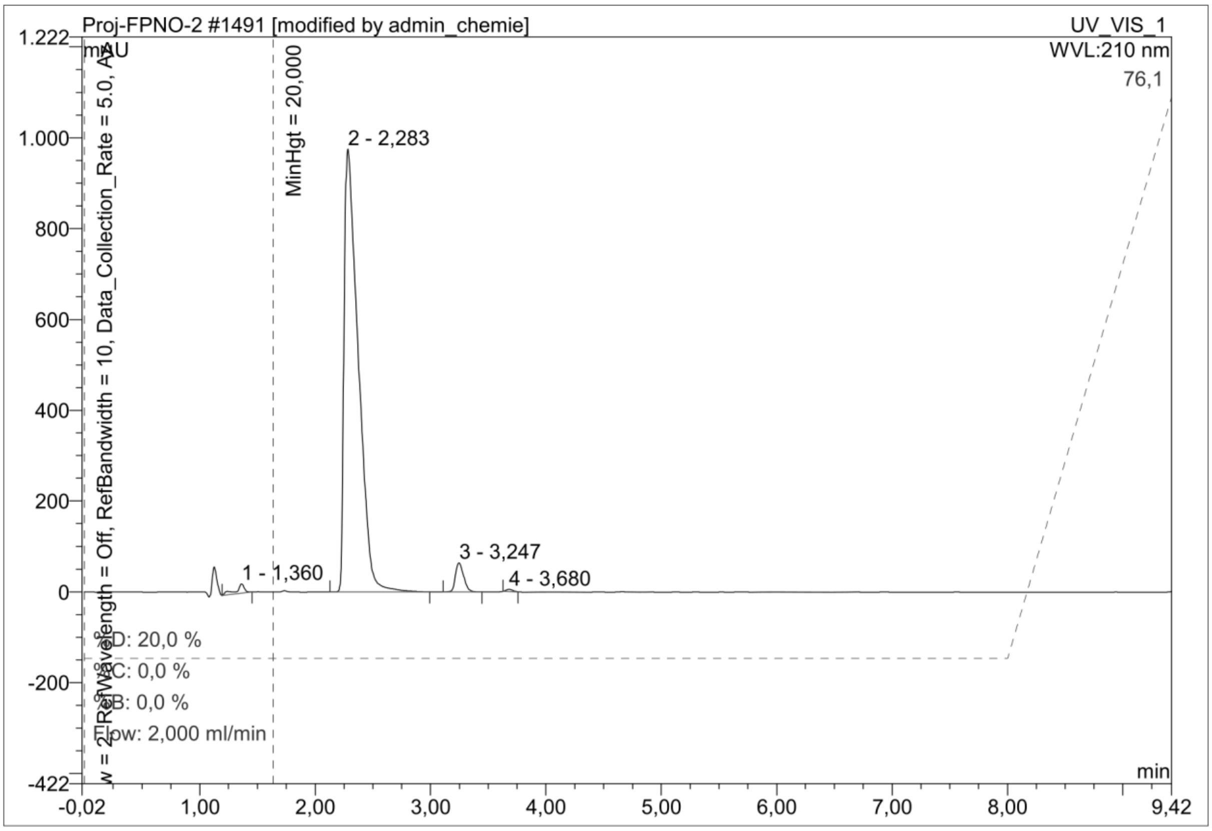

No.	Ret.Time min	Peak Name	Height mAU	Area mAU*min	Rel.Area %	Amount	Type
1	1,36	n.a.	20,540	1,722	1,18	n.a.	BMB*
2	2,28	n.a.	975,364	138,829	94,99	n.a.	BMB
3	3,25	n.a.	64,040	5,280	3,61	n.a.	BMB
4	3,68	n.a.	4,706	0,317	0,22	n.a.	BMB*
**Total:**			1064,650	146,149	100,00	0,000	

HPLC trace of (*RS*)-FET-OMe (UV, λ = 210 nm). Column: Chromolith HighResolution RP-18e, 150×4.6 mm; eluent: 20% MeCN (0.1% TFA); flow rate: 2.0 mL/min.

HPLC chromatogram of (*RS*)-FET

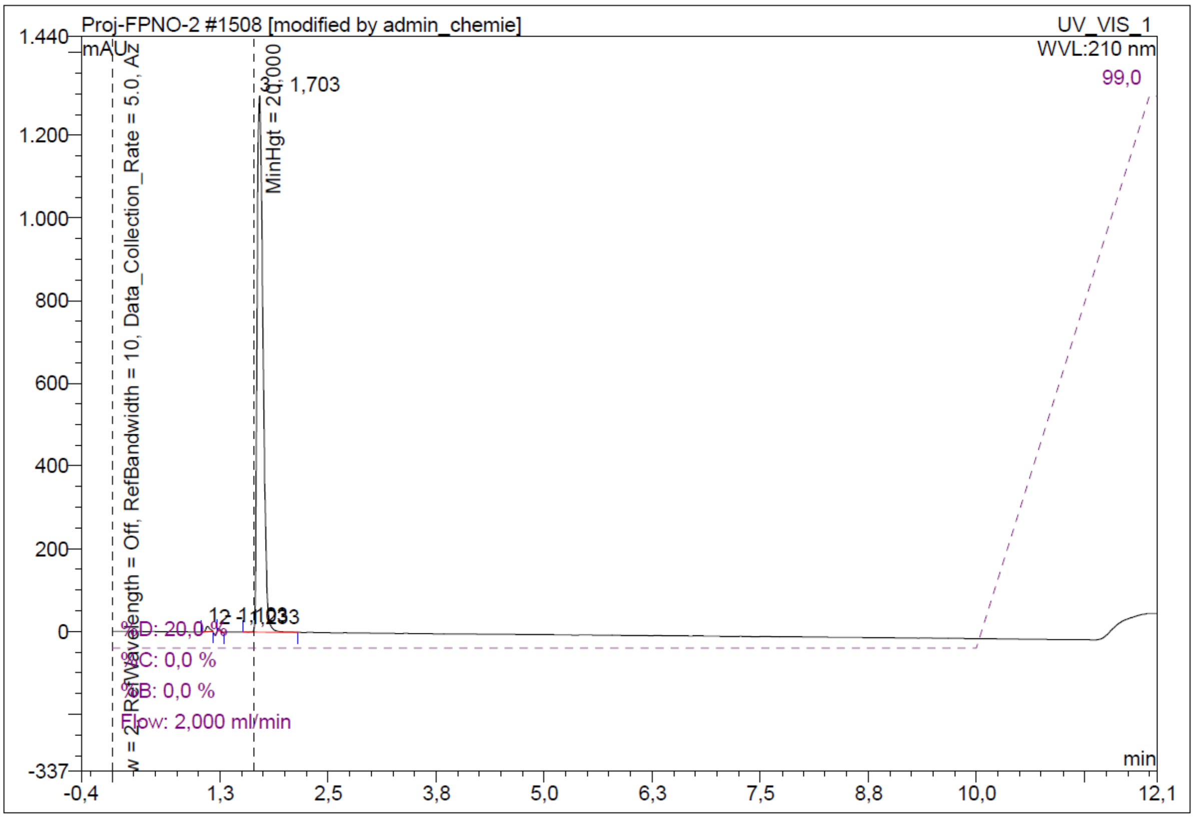

No.	Ret.Time min	Peak Name	Height mAU	Area mAU*min	Rel.Area %	Amount	Type
1	1,10	n.a.	12,475	0,632	0,61	n.a.	BMB*
2	1,23	n.a.	5,942	0,085	0,08	n.a.	BMB*
3	1,70	n.a.	1297,007	102,624	99,31	n.a.	BMB*
**Total:**			1315,424	103,340	100,00	0,000	

HPLC trace of (*RS*)-FET (UV, λ = 210 nm). Column: Chromolith HighResolution RP-18e, 150×4.6 mm; eluent: 20% MeCN (0.1% TFA); flow rate: 2.0 mL/min.

2.2 Chiral HPLC chromatograms of racemic and enantiomerically pure *m*-FET

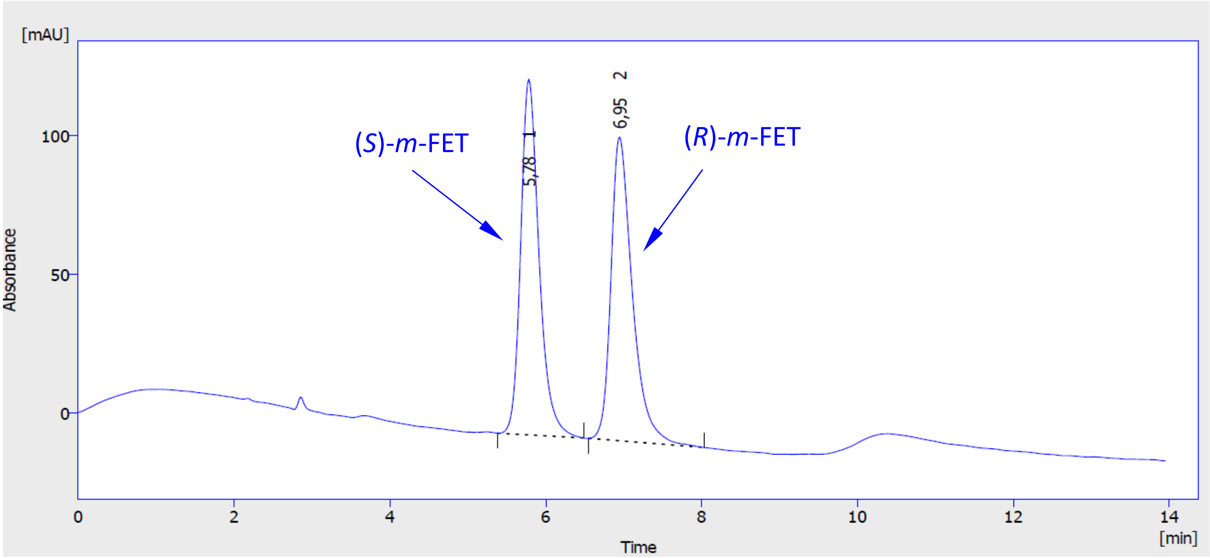

Result Table (Uncal - Data\ee_2024_03_08_14_BZD762_mFET_rac_chirobiotic_T - UV: Channel 1)

	Reten. Time [min]	Area [mAU.s]	Height [mAU]	Area [%]
1	5,783	2099,464	128,447	50,0
2	6,950	2096,656	109,627	50,0
	Total	4196,120	238,074	100,0

Chiral HPLC trace of (*RS*)-*m*-FET (UV, λ = 254 nm). Column: Astec Chirobiotic T, 250×4.6 mm; eluent: 55% MeOH (0.02% HCO_2H); flow rate: 1.0 mL/min; (*S*)-*m*-FET: t_R = 5.78 min; (*R*)-*m*-FET: t_R = 6.95 min.

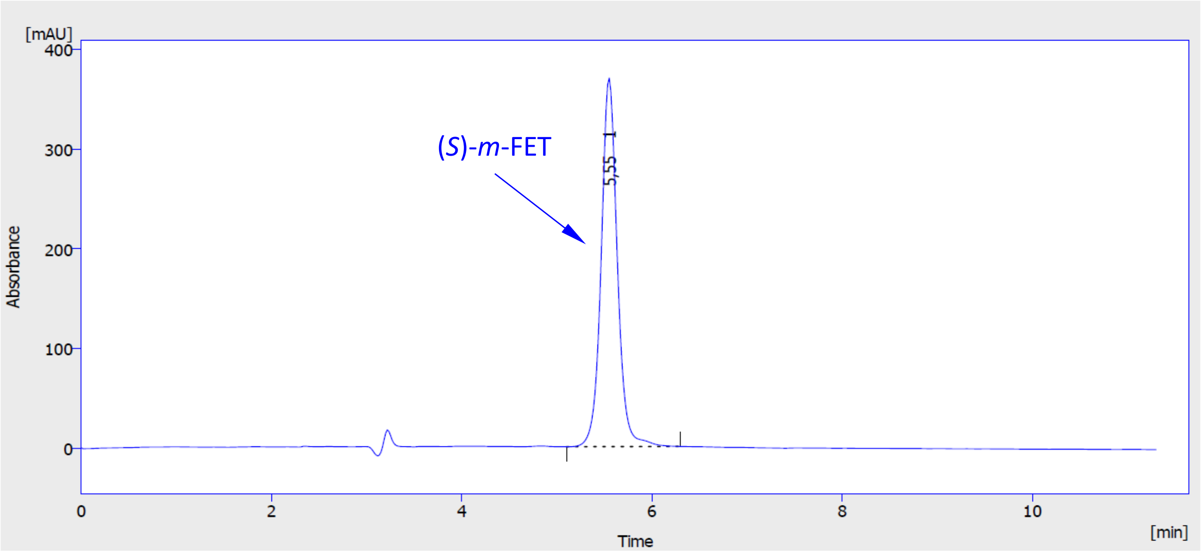

Result Table (Uncal - Data\ee_2024_03_19_37_(S)-m-FET_P1_chirobiotic_T - UV: Channel 1)

	Reten. Time [min]	Area [mAU.s]	Height [mAU]	Area [%]
1	5,550	4354,987	368,977	100,0
	Total	4354,987	368,977	100,0

Chiral HPLC trace of (*S*)-*m*-FET (UV, λ = 254 nm). Column: Astec Chirobiotic T, 250×4.6 mm; eluent: 55% MeOH (0.02% HCO_2H); flow rate: 1.0 mL/min; (*S*)-*m*-FET: t_R = 5.55 min.

2.3 Chiral HPLC chromatograms of racemic and enantiomerically pure FET

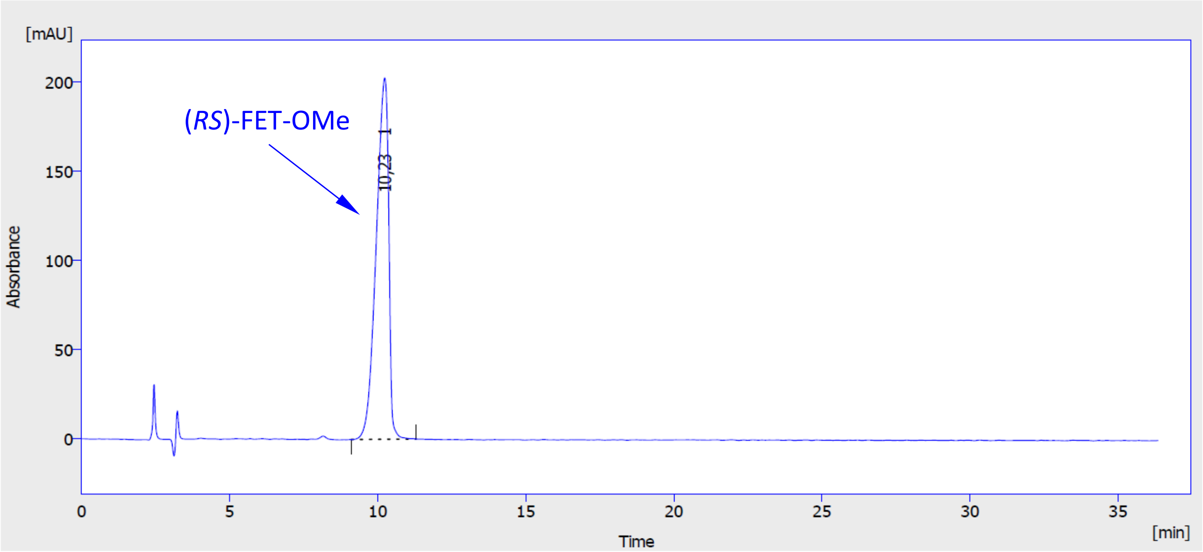

Result Table (Uncal - Data\ee_2024_03_11_27_BZD760_FET_CO2Me_rac_chirobiotic_T - UV: Channel 1)

	Reten. Time [min]	Area [mAU.s]	Height [mAU]	Area [%]
1	10,233	5957,145	202,741	100,0
	Total	5957,145	202,741	100,0

Chiral HPLC trace of (*RS*)-FET-OMe (UV, λ = 254 nm). Note that there was no separation of enantiomers. Column: Astec Chirobiotic T, 250×4.6 mm; eluent: 55% MeOH (0.02% HCO_2H); flow rate: 1.0 mL/min; (*RS*)-FET-OMe: t_R = 10.23 min.

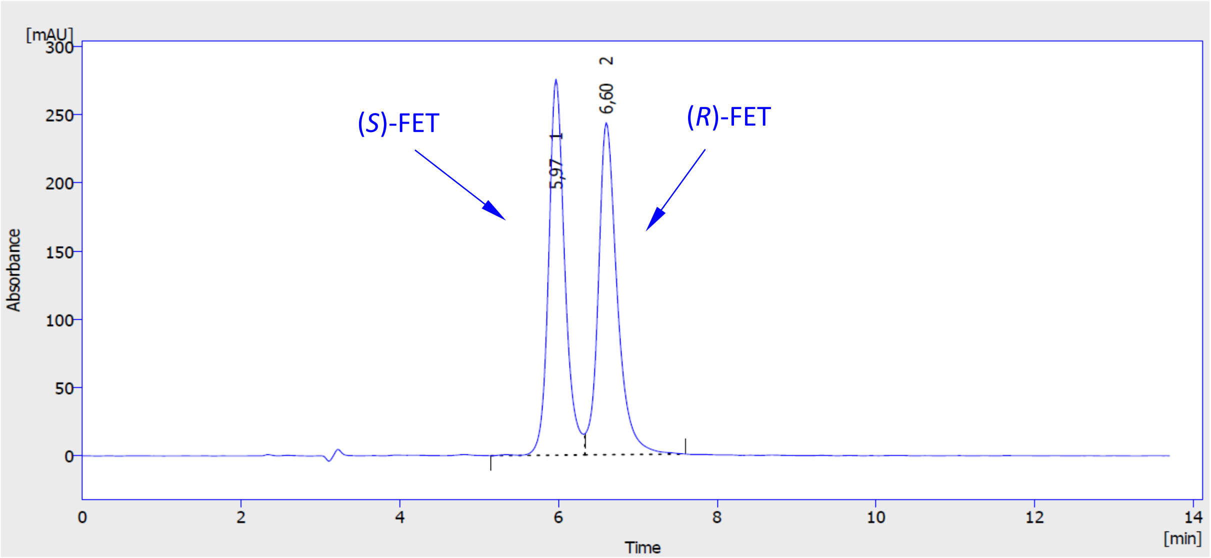

Result Table (Uncal - Data\ee_2024_03_14_31_BZD765_p-FET-rac_chirobiotic_T - UV: Channel 1)

	Reten. Time [min]	Area [mAU.s]	Height [mAU]	Area [%]
1	5,967	3898,063	275,439	49,1
2	6,600	4033,263	243,330	50,9
	Total	7931,326	518,769	100,0

Chiral HPLC trace of (*RS*)-FET prepared by hydrolysis of (*RS*)-FET-OMe with HCl (UV, λ = 254 nm). Column: Astec Chirobiotic T, 250×4.6 mm; eluent: 55% MeOH (0.02% HCO_2H); flow rate: 1.0 mL/min; (*S*)-FET: t_R = 5.97 min; (*R*)-FET: t_R = 6.60 min.

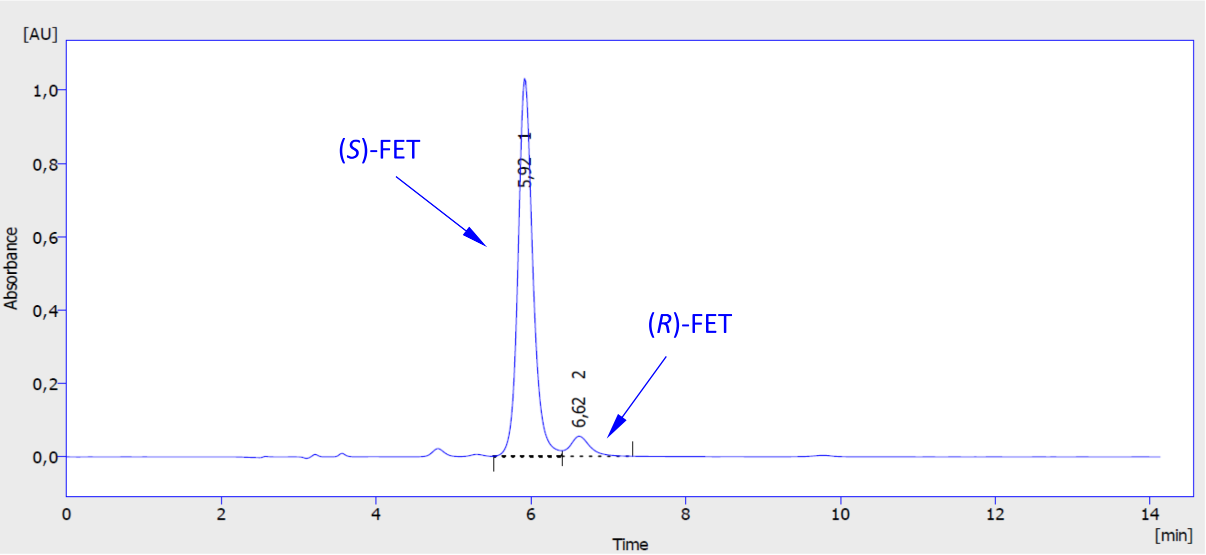

Result Table (Uncal - Data\ee_2024_03_14_33_CH123_p-FET_chirobiotic_T - UV: Channel 1)

	Reten. Time [min]	Area [mAU.s]	Height [mAU]	Area [%]
1	5,917	13755,948	1030,394	93,3
2	6,617	986,291	54,391	6,7
	Total	14742,239	1084,785	100,0

Chiral HPLC trace of (*S*)-FET prepared by hydrolysis of (*S*)-FET-OMe with HCl (UV, λ = 254 nm). Column: Astec Chirobiotic T, 250×4.6 mm; eluent: 55% MeOH (0.02% HCO_2H); flow rate: 1.0 mL/min; (*S*)-FET: t_R = 5.92 min; (*R*)-FET: t_R = 6.62 min.

2.4 Chiral HPLC chromatogram of **8**

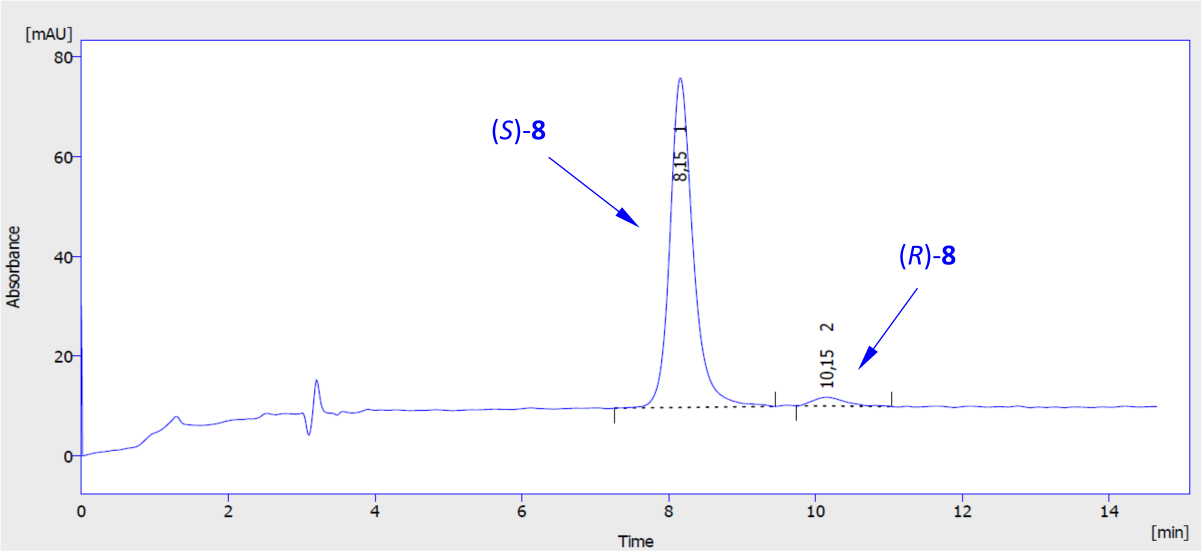

Result Table (Uncal - Data\ee_2024_03_21_41_BZD768a_mFET_Prec_hydro_chirobiotic_T - UV: Channel 1)

	Reten. Time [min]	Area [mAU.s]	Height [mAU]	Area [%]
1	8,150	1454,859	66,013	96,3
2	10,150	55,474	1,768	3,7
	Total	1510,333	67,781	100,0

Chiral HPLC trace of **8** (UV, λ = 254 nm). Column: Astec Chirobiotic T, 250×4.6 mm; eluent: 55% MeOH (0.02% HCO_2H); flow rate: 1.0 mL/min; (*S*)-**8**: t_R = 8.15 min; (*R*)-**8**: t_R = 10.15 min.

2.5 HPLC chromatograms of *m*-[^{18}F]FET

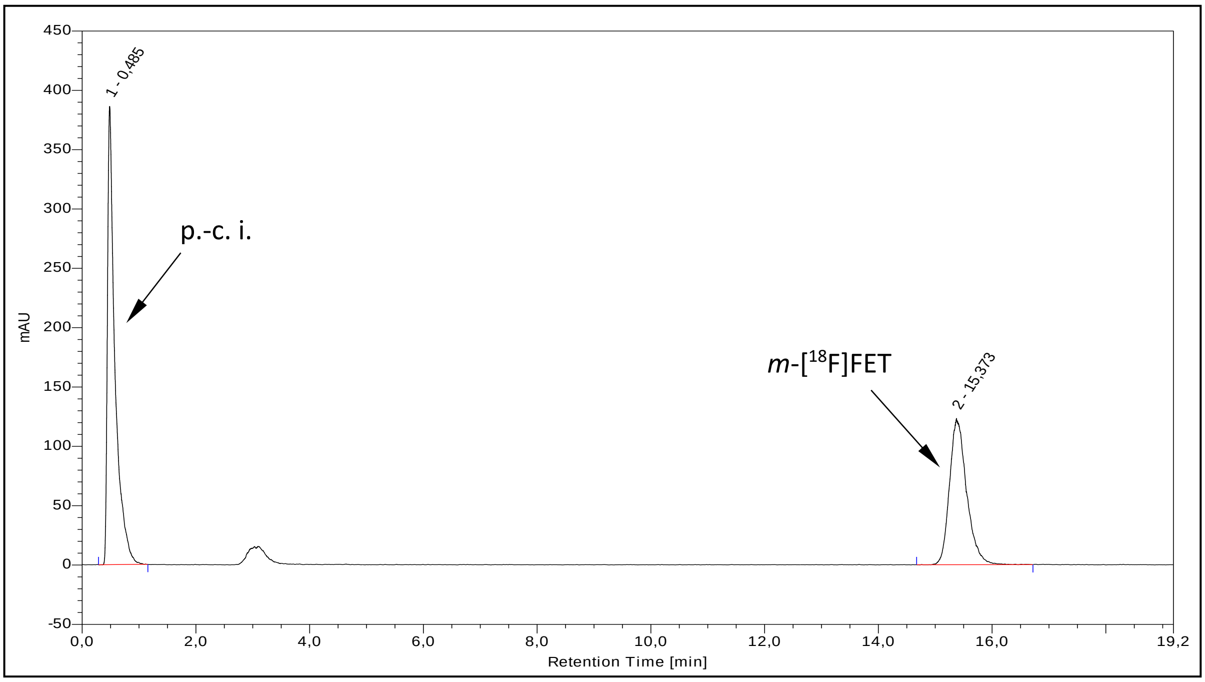

HPLC trace of crude *m*-[^{18}F]FET [eluent: 10% MeCN (0.1% TFA); flow rate: 1 mL/min]. Abbreviation: p.-c.i. – post column injection.

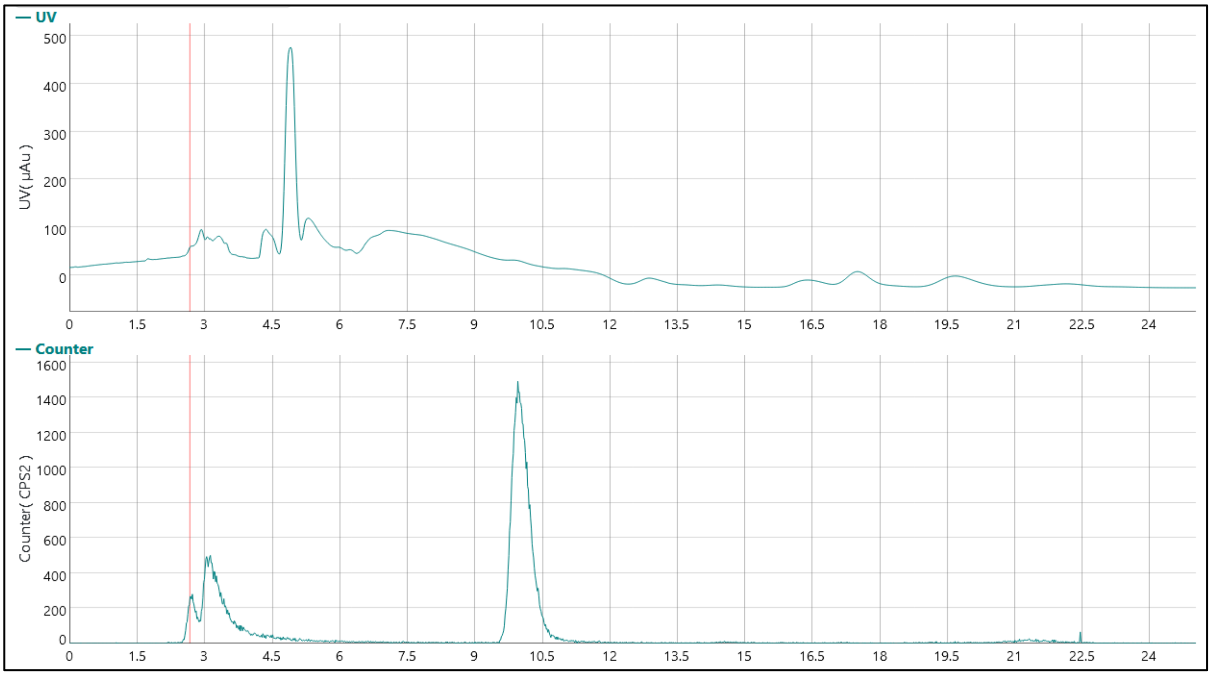

HPLC traces for purification of *m*-[^{18}F]FET by semi-preparative HPLC (top trace: UV, λ = 254 nm; bottom trace: radioactivity; column: Hydro-RP, 250×10 mm; eluent: 10% EtOH [300 mg/L NH_4OAc]; flow rate: 4.7 mL/min; t_R = 10.1 min).

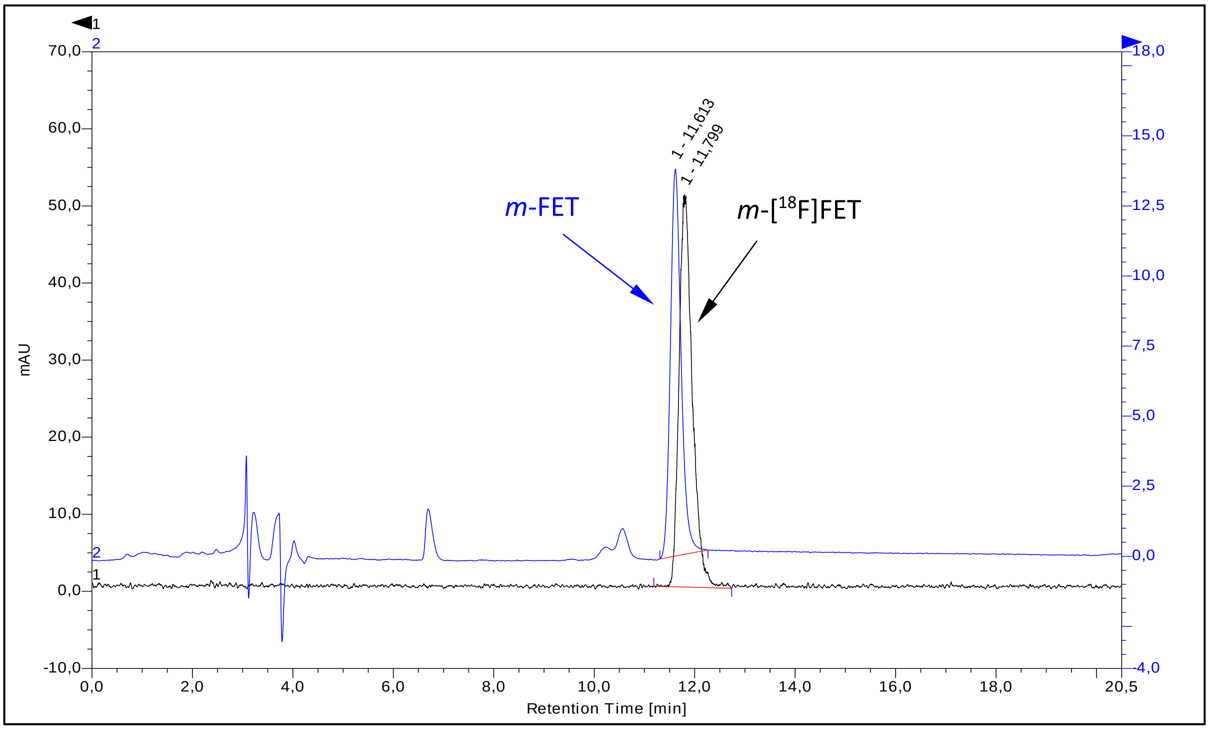

HPLC traces of purified *m*-[^{18}F]FET spiked with the non-radioactive reference compound *m*-FET (eluent: 10% EtOH [300 mg/L NH_4OAc]; flow rate: 1 mL/min). Blue trace: UV, λ = 254 nm; black trace: radioactivity.

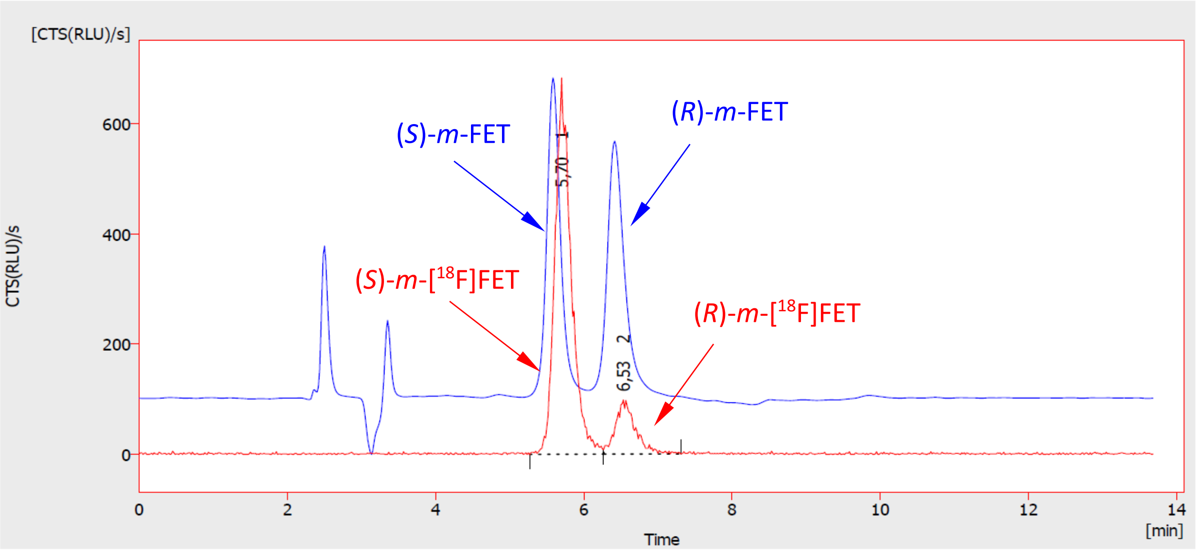

Result Table (Uncal - Data|18_03_2024_16_[18F]m-FET_ee_determination_Bene_Prec - HERM)

	Reten. Time [min]	Area [CTS(RLU)/s.s]	Height [CTS(RLU)/s]	Area [%]
1	5,700	10357,366	682,789	85,0
2	6,533	1822,134	98,382	15,0
	Total	12179,500	781,171	100,0

Chiral HPLC traces of purified *m*-[^{18}F]FET (prepared using $Bu_4NOH·30\ H_2O$ in MeCN for [^{18}F]F^- elution) spiked with the non-radioactive reference compound (*RS*)-*m*-FET. Blue trace: UV, λ = 254 nm; red trace: radioactivity. Column: Astec Chirobiotic T, 250×4.6 mm; eluent: 55% MeOH (0.02% HCO_2H); flow rate: 1.0 mL/min; (*S*)-*m*-[^{18}F]FET: t_R = 5.70 min; (*R*)-*m*-[^{18}F]FET: t_R = 6.53 min.

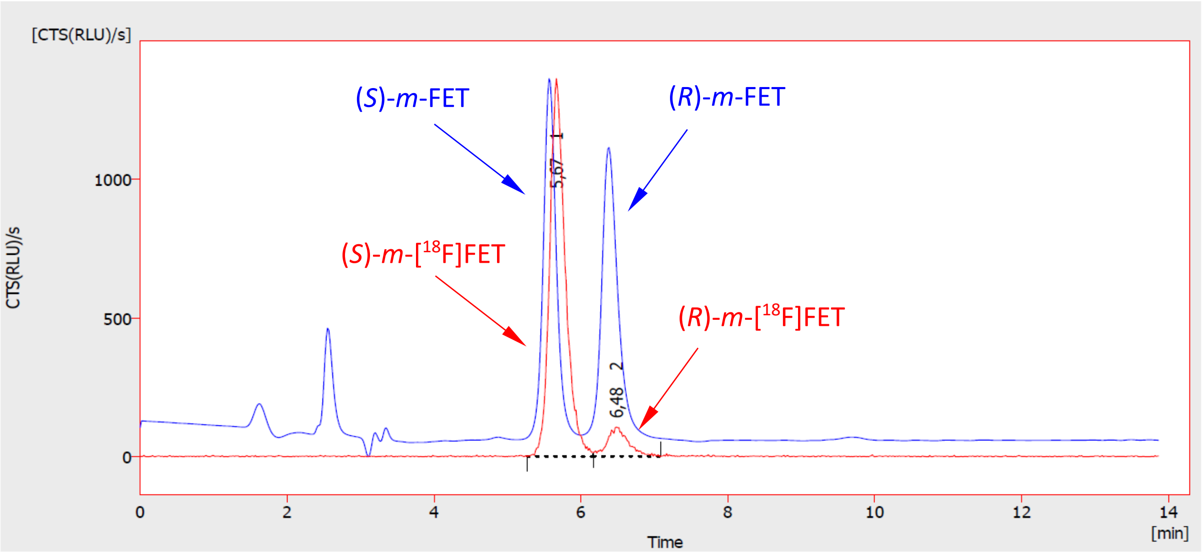

Result Table (Uncal - Data\21_03_2024_21_[18F]mFET_ee_determination_Bene_Prec - HERM)

	Reten. Time [min]	Area [CTS(RLU)/s.s]	Height [CTS(RLU)/s]	Area [%]
1	5,667	20072,000	1361,000	90,5
2	6,483	2113,000	106,000	9,5
	Total	22185,000	1467,000	100,0

Chiral HPLC traces of purified *m*-[^{18}F]FET (prepared using Bu_4NOTs in MeOH for [^{18}F]F^- elution) spiked with the non-radioactive reference compound (*RS*)-*m*-FET. Blue trace: UV, λ = 254 nm; red trace: radioactivity. Column: Astec Chirobiotic T, 250×4.6 mm; eluent: 55% MeOH (0.02% HCO_2H); flow rate: 1.0 mL/min; (*S*)-*m*-[^{18}F]FET: t_R = 5.67 min; (*R*)-*m*-[^{18}F]FET: t_R = 6.48 min.

2.6 HPLC chromatograms of [^{18}F]FET-OMe

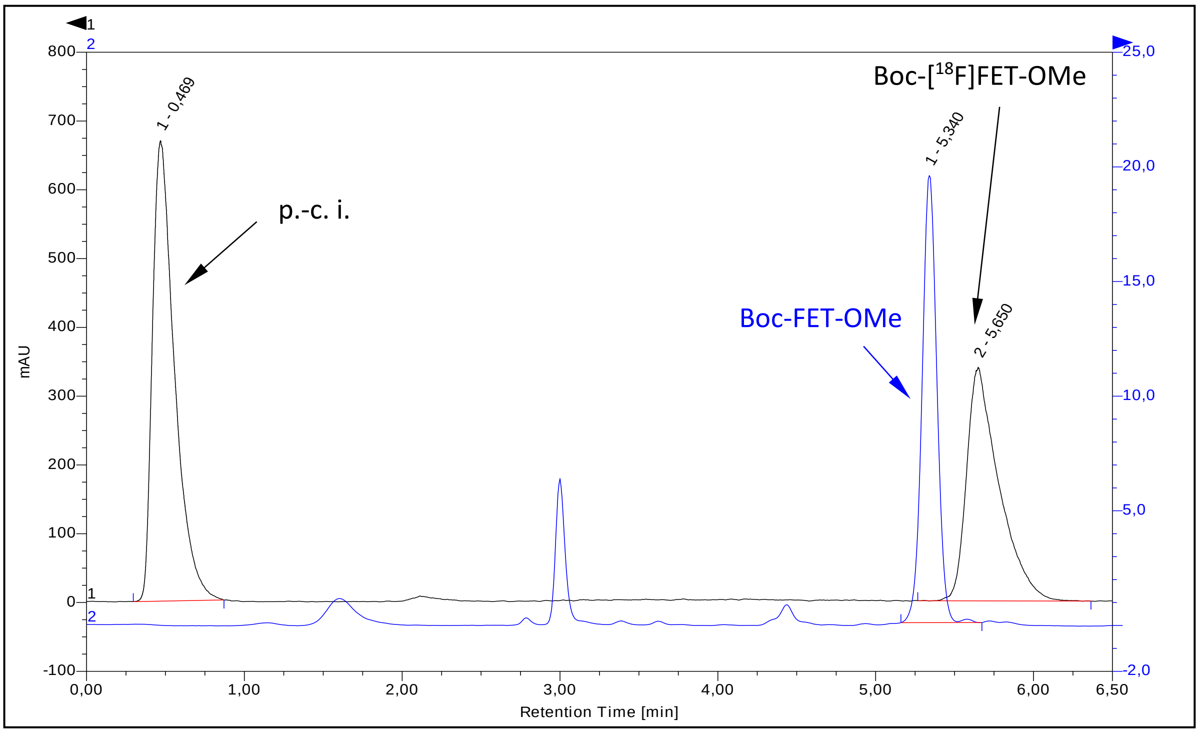

HPLC traces of Boc-[^{18}F]FET-OMe spiked with the non-radioactive reference compound Boc-FET-OMe (eluent: 65% MeCN; flow rate: 1 mL/min). Blue trace: UV, λ = 254 nm; black trace: radioactivity. Abbreviation: p.-c. i. – post column injection.

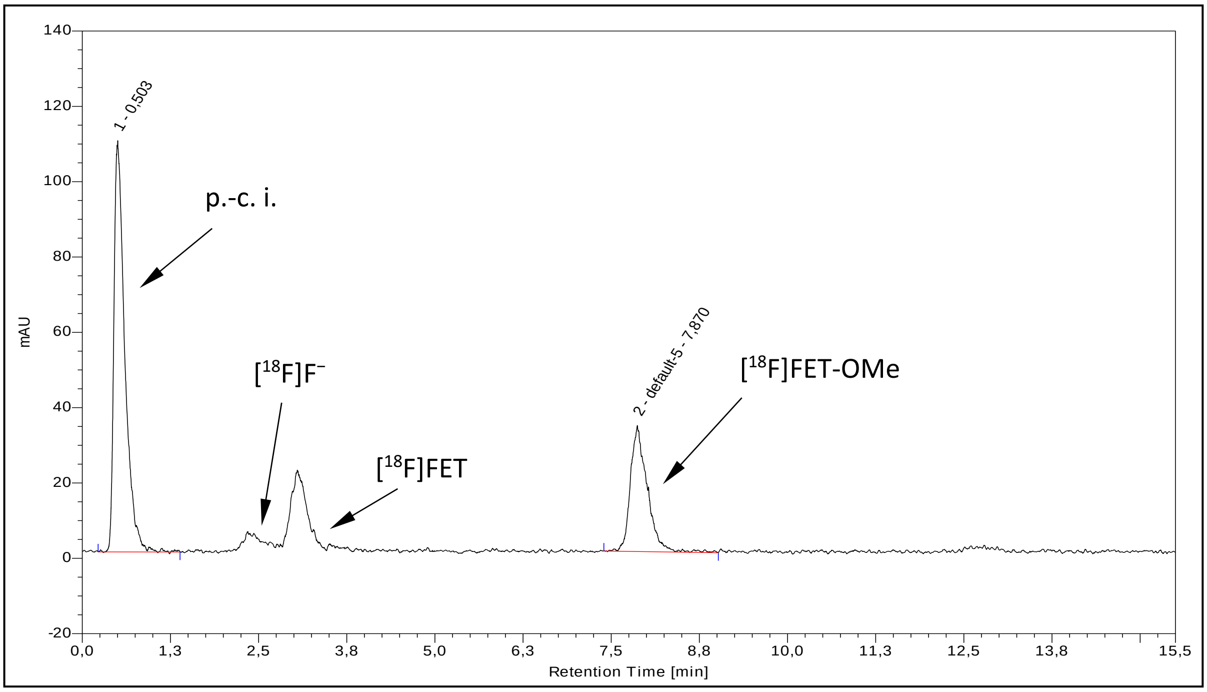

HPLC trace of [^{18}F]FET-OMe [eluent: 15% MeCN (0.1% TFA); flow rate: 1 mL/min]. Abbreviation: p.-c.i. – post column injection.

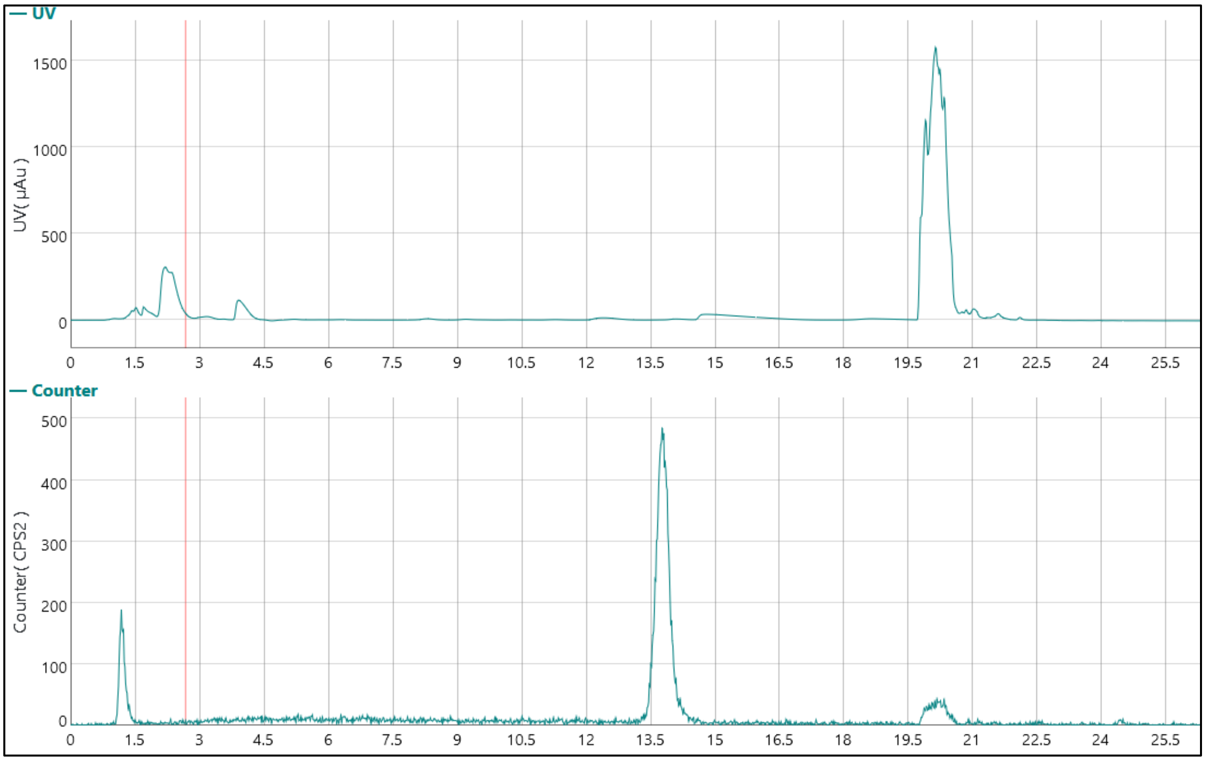

HPLC traces for purification of [^{18}F]FET-OMe by semi-preparative HPLC (top trace: UV, λ = 254 nm; bottom trace: radioactivity; column: Hydro-RP, 250×10 mm; eluent: 15% MeCN (0.1% TFA); flow rate: 4.7 mL/min; t_R = 13.8 min).

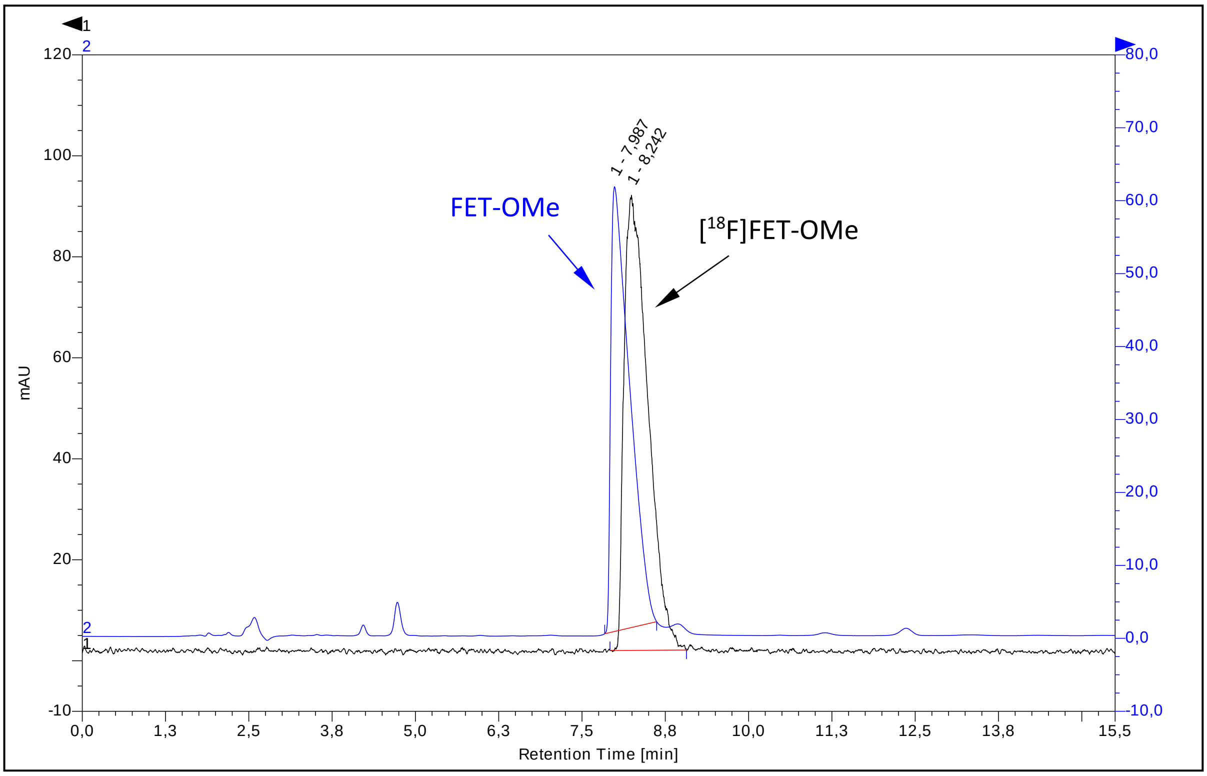

HPLC traces of purified [^{18}F]FET-OMe spiked with the non-radioactive reference compound FET-OMe [eluent: 15% MeCN (0.1% TFA); flow rate: 1 mL/min]. Blue trace: UV, λ = 254 nm; black trace: radioactivity.

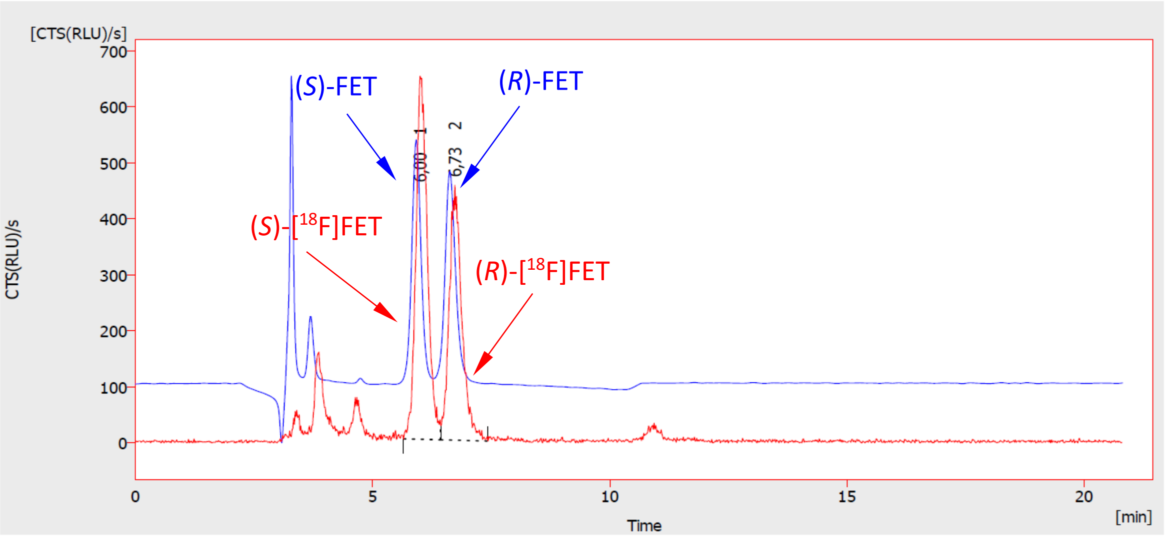

Result Table (Uncal - Data\18_03_2024_20_[18F]p-FET_ee_determination_Bene_Prec - HERM)

	Reten. Time [min]	Area [CTS(RLU)/s.s]	Height [CTS(RLU)/s]	Area [%]
1	6,000	10934,290	648,785	56,6
2	6,733	8388,710	455,430	43,4
	Total	19323,000	1104,215	100,0

Chiral HPLC traces of [^{18}F]FET (obtained by hydrolysis of purified [^{18}F]FET-OMe with 12 M HCl) spiked with the non-radioactive reference compound (*RS*)-FET. [^{18}F]FET-OMe was prepared using Bu_4NOH·30 H_2O in MeCN for [^{18}F]F$^-$ elution. Blue trace: UV, λ = 254 nm; red trace: radioactivity. Column: Astec Chirobiotic T, 250×4.6 mm; eluent: 55% MeOH (0.02% HCO_2H); flow rate: 1.0 mL/min; (*S*)-[^{18}F]FET: t_R = 6.00 min; (*R*)-[^{18}F]FET: t_R = 6.73 min.

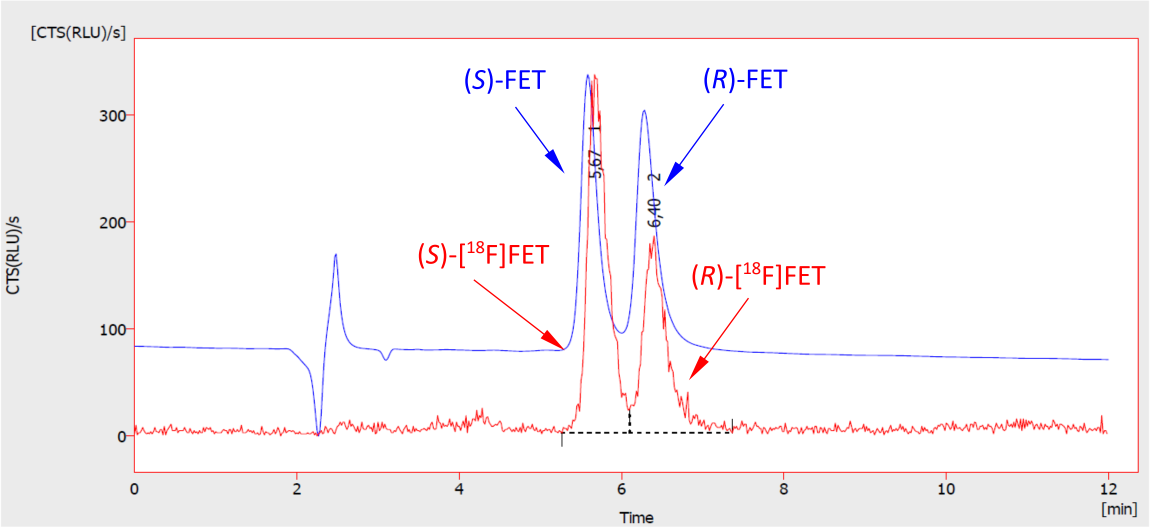

Result Table (Uncal - Data\22_03_2024_22_p-[18F]FET-CO2Me_ee_determination_Bene_Prec - HERM)

	Reten. Time [min]	Area [CTS(RLU)/s.s]	Height [CTS(RLU)/s]	Area [%]
1	5,667	5828,000	335,000	60,3
2	6,400	3836,000	184,000	39,7
	Total	9664,000	519,000	100,0

Chiral HPLC traces of [^{18}F]FET (obtained by hydrolysis of purified [^{18}F]FET-OMe with 12 M HCl) spiked with the non-radioactive reference compound (*RS*)-FET. [^{18}F]FET-OMe was prepared using Bu_4NOTs in MeOH for [^{18}F]F$^-$ elution. Blue trace: UV, λ = 254 nm; red trace: radioactivity. Column: Astec Chirobiotic T, 250×4.6 mm; eluent: 55% MeOH (0.02% HCO_2H); flow rate: 1.0 mL/min; (*S*)-[^{18}F]FET: t_R = 5.67 min; (*R*)-[^{18}F]FET: t_R = 6.40 min.

3 Determination of molar activity of *m*-[^{18}F]FET and [^{18}F]FET-OMe

A sample of the purified tracer solution (20 µL) was analysed by analytical HPLC and the carrier amount was determined from the peak area according to the respective calibration curve. The molar activity (A_M) was calculated according to following formula:

$$A_M = \frac{(GBq)}{(\mu mol)}$$

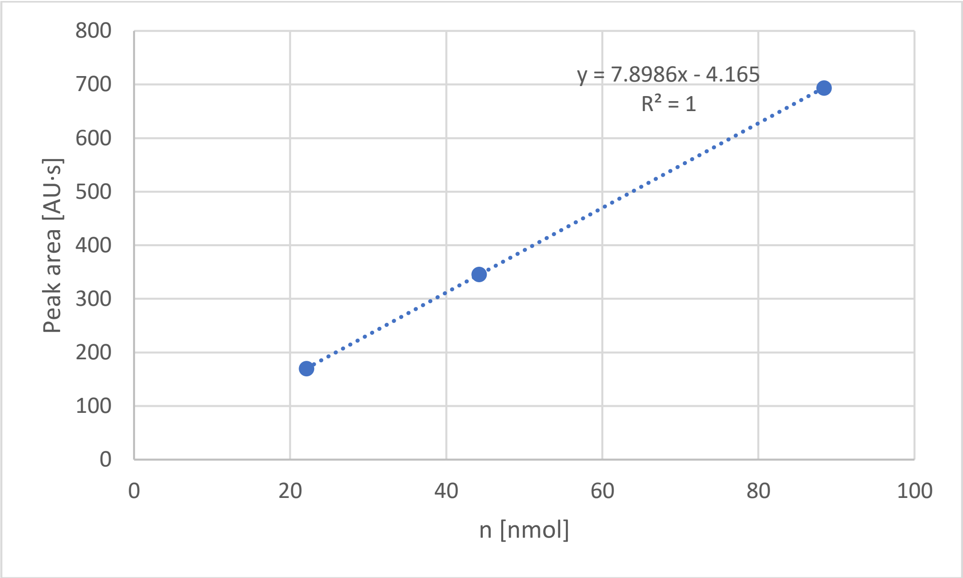

Figure S1: Calibration curve for determination of carrier amount of *m*-[^{18}F]FET. Raw data are shown in Table S1.

Table S1: Calibration data (measured at 254 nm) for determination of carrier amount of *m*-[^{18}F]FET.

Concentration [µM]	n [nmol]	Peak area [AU·s]
**1**	88.4	693.83
**0.5**	44.2	345.72
**0.25**	22.1	169.89

Table S2: Calculation of molar activity of *m*-[^{18}F]FET.

Activity in 20 µL [GBq]	6.36×10^{-4}
Peak area [AU·s]	0.053
Carrier amount [nmol]	6.71×10^{-4}
Molar activity [GBq/µmol]	94.8

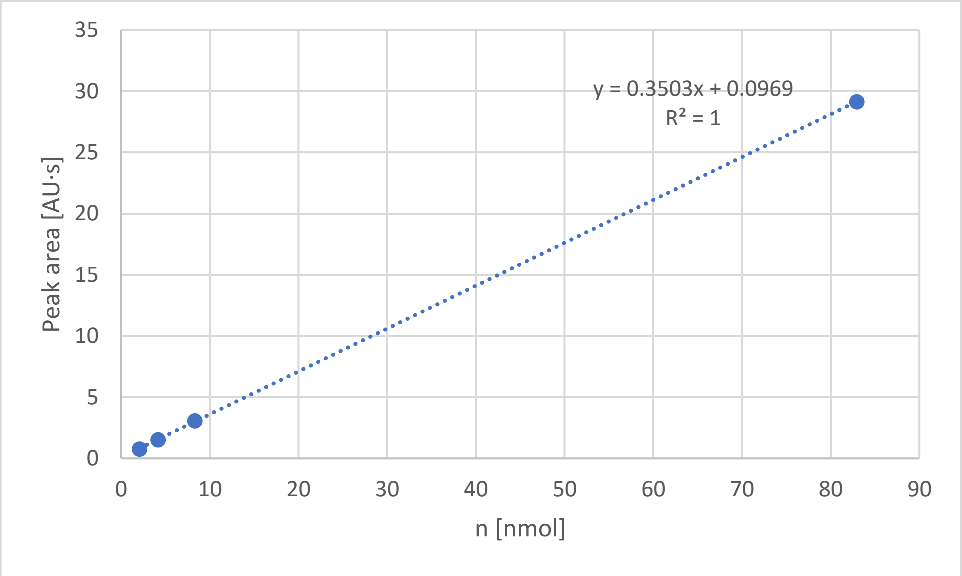

Figure S2: Calibration curve for determination of carrier amount of [^{18}F]FET-OMe. Raw data are shown in Table S3.

Table S3: Calibration data (measured at 254 nm) for determination of carrier amount of [^{18}F]FET-OMe.

Concentration [µM]	n [nmol]	Peak area [AU·s]
**1**	82.9	29.15
**0.1**	8.3	3.06
**0.05**	4.1	1.54
**0.025**	2.1	0.78

Table S4: Calculation of molar activity of [^{18}F]FET-OMe.

Activity in 20 µL [GBq]	1.17×10^{-3}
Peak area [AU·s]	0.0026
Carrier amount [nmol]	7.42×10^{-4}
Molar activity [GBq/µmol]	157.4

4 Elution experiments with Bu_4NOTs and Bu_4NOH

Table S5: pH value of $Bu_4NOH·30\ H_2O$ and Bu_4NOTs solutions before and after elution of QMA cartridges (pre-conditioned with H_2O) and after heating at 85 °C for 5 min.

Elution salt	Sample	pH		
		Before elution	After elution	After heating
$Bu_4NOH·30\ H_2O$	1	12.66	11.44	11.68
	2	12.66	11.60	11.37
	3	12.67	11.17	11.10
Bu_4NOTs	1	5.24	8.89	9.04
	2	5.46	8.69	8.89
	3	5.43	8.71	8.99

Table S6: pH value of Bu_4NOTs solutions before and after elution of QMA cartridges (pre-conditioned with 0.05 M $NaHCO_3$ and H_2O) and after heating at 85 °C for 5 min.

Elution salt	Sample	pH		
		Before elution	After elution	After heating
Bu_4NOTs	1	5.32	9.54	9.35
	2	5.07	9.56	9.48
	3	5.06	9.54	9.35

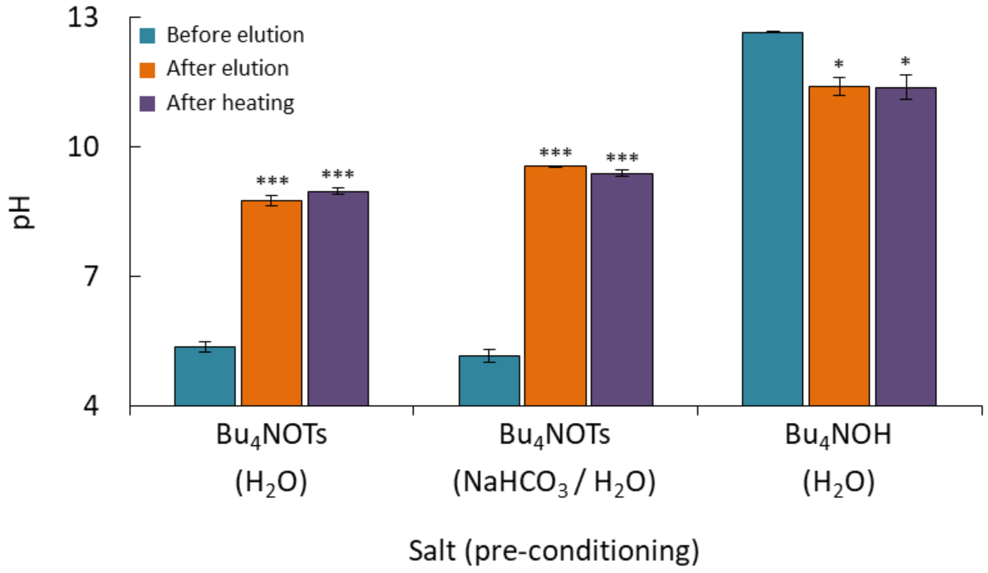

Figure S3: pH-values of aqueous Bu_4NOTs or Bu_4NOH solutions determined before and after elution of QMA anion exchange cartridges (pre-conditioned with 2 mL H_2O or with 10 mL 0.05 M $NaHCO_3$ followed by 10 mL H_2O as indicated) and after heating of the eluates thus obtained at 85 °C for 5 min (n=3 per experimental condition). Statistically significant differences compared to the corresponding pH values before elution were identified by Welch's ANOVA with Games-Howell post-hoc test and are indicated by asterisks (*: $p<0.05$, ***: $p\leq0.001$).

5 Protein incorporation

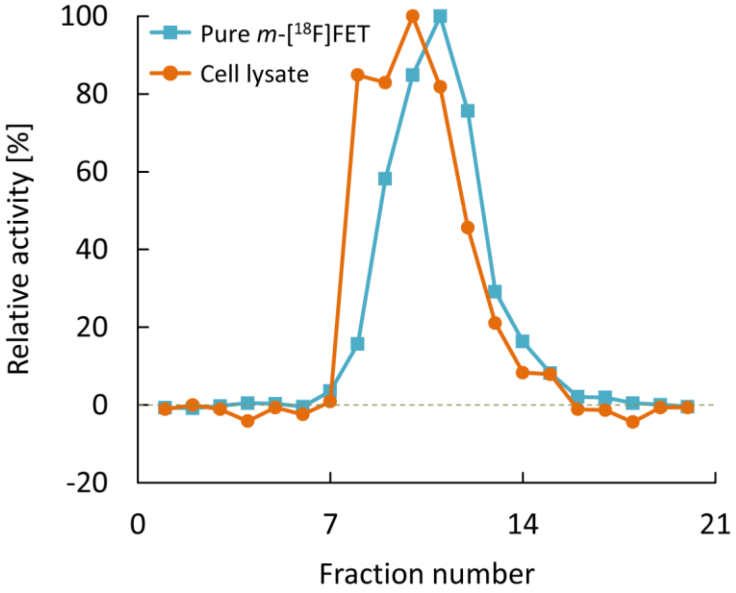

Figure S4: Elution profile of radioactivity from PD 10 cartridges loaded with the soluble fraction of homogenized U87 MG cells incubated with *m*-[^{18}F]FET for 1 h (orange) or with pure *m*-[^{18}F]FET (control experiments without incubation, turquoise). The cartridges were eluted with 20 × 1 mL of Earle's balanced salt solution (EBSS) and the radioactivity in each fraction was measured with a gamma counter, corrected for background activity and normalized by the maximum activity value. Note that the apparently negative values observed in some cases are an artefact introduced by the background subtraction that can be attributed to slight variations in background radioactivity.
